# A Two-Component Regulatory System Impacts Extracellular Membrane-Derived Vesicle Production in Group A Streptococcus

**DOI:** 10.1128/mBio.00207-16

**Published:** 2016-11-01

**Authors:** Ulrike Resch, James Anthony Tsatsaronis, Anaïs Le Rhun, Gerald Stübiger, Manfred Rohde, Sergo Kasvandik, Susanne Holzmeister, Philip Tinnefeld, Sun Nyunt Wai, Emmanuelle Charpentier

**Affiliations:** a The Laboratory for Molecular Infection Medicine Sweden (MIMS), Department of Molecular Biology, Umeå University, Umeå, Sweden; b Department of Regulation in Infection Biology, Helmholtz Centre for Infection Research, Braunschweig, Germany; c Department of Regulation in Infection Biology, Max Planck Institute for Infection Biology, Berlin, Germany; d Department of Biomedical Imaging and Image-Guided Therapy, Medical University, Vienna, Vienna, Austria; e Central Facility for Microscopy, Helmholtz Centre for Infection Research, Braunschweig, Germany; f University of Tartu, Institute of Technology, Tartu, Estonia; g NanoBioScience Group, Institute for Physical and Theoretical Chemistry, TU, Braunschweig, Braunschweig, Germany; h Hannover Medical School, Hannover, Germany

## Abstract

Export of macromolecules via extracellular membrane-derived vesicles (MVs) plays an important role in the biology of Gram-negative bacteria. Gram-positive bacteria have also recently been reported to produce MVs; however, the composition and mechanisms governing vesiculogenesis in Gram-positive bacteria remain undefined. Here, we describe MV production in the Gram-positive human pathogen group A streptococcus (GAS), the etiological agent of necrotizing fasciitis and streptococcal toxic shock syndrome. M1 serotype GAS isolates in culture exhibit MV structures both on the cell wall surface and in the near vicinity of bacterial cells. A comprehensive analysis of MV proteins identified both virulence-associated protein substrates of the general secretory pathway in addition to “anchorless surface proteins.” Characteristic differences in the contents, distributions, and fatty acid compositions of specific lipids between MVs and GAS cell membrane were also observed. Furthermore, deep RNA sequencing of vesicular RNAs revealed that GAS MVs contained differentially abundant RNA species relative to bacterial cellular RNA. MV production by GAS strains varied in a manner dependent on an intact two-component system, CovRS, with MV production negatively regulated by the system. Modulation of MV production through CovRS was found to be independent of both GAS cysteine protease SpeB and capsule biosynthesis. Our data provide an explanation for GAS secretion of macromolecules, including RNAs, lipids, and proteins, and illustrate a regulatory mechanism coordinating this secretory response.

## IMPORTANCE

Group A streptococcus (GAS) is a Gram-positive bacterial pathogen responsible for more than 500,000 deaths annually. Establishment of GAS infection is dependent on a suite of proteins exported via the general secretory pathway. Here, we show that GAS naturally produces extracellular vesicles with a unique lipid composition that are laden with proteins and RNAs. Interestingly, both virulence-associated proteins and RNA species were found to be differentially abundant in vesicles relative to the bacteria. Furthermore, we show that genetic disruption of the virulence-associated two-component regulator CovRS leads to an increase in vesicle production. This study comprehensively describes the protein, RNA, and lipid composition of GAS-secreted MVs and alludes to a regulatory system impacting this process.

## INTRODUCTION

Infections by the Gram-positive human pathogen *Streptococcus pyogenes*, also known as group A streptococcus (GAS), are responsible for global mortality and high societal costs in both developed and developing countries. Conservative estimates of the global mortality burden of GAS disease broach 500,000 deaths per year (1). Noninvasive GAS disease also represents a significant financial burden, with pediatric cases of pharyngitis in the United States alone inflicting a societal cost of $539 million per year (2). Despite concerted effort, no vaccines are currently licensed for commercial use against GAS infections (3).

Both mild and severe forms of GAS diseases are mediated by a suite of secreted and membrane-associated virulence factors that interact with the human host (3, 4). Trafficking and secretion of GAS proteins have long been assumed to be solely dependent on the canonical general secretory (Sec) translocation pathway. In GAS, the Sec pathway is associated with the ExPortal, a cell membrane microdomain enriched for proteins of the pathway (5). Interestingly, previous studies illustrate the presence of multiple factors present in the GAS secretome that lack the N-terminal secretion signal peptide or C-terminal cell wall anchor motif required for Sec-mediated translocation (6). These factors, dubbed “anchorless surface proteins,” comprise many important GAS virulence determinants and vaccine candidates; however, the mechanisms responsible for transport of these factors across the cell membrane remain unexplained (7).

In Gram-negative bacteria, outer membrane vesicles (OMVs) are actively formed through the budding and release of the outer membrane and contain numerous virulence-associated and immunomodulatory factors (8). OMVs have been implicated in many aspects of Gram-negative bacterial pathogenesis, including delivery of protein toxins, triggering host inflammatory responses and transfer of nucleic acids and providing protection against phages and antimicrobial peptides (9). Recent seminal works describe production of extracellular membrane-derived vesicles (MVs) by several clinically relevant species of Gram-positive bacteria, such as *Staphylococcus aureus*, *Bacillus anthracis*, and *Streptococcus pneumoniae* (10–12). Two recent reports indicate that GAS also releases MVs and that this process increases following sublethal penicillin or LL-37 treatment (13, 14). *Mycobacterium tuberculosis* and other mycobacterial species also actively produce MVs, which are able to modulate host inflammatory responses (15). These studies hint at a more broadly conserved export strategy utilized by both Gram-negative and Gram-positive bacteria (16). In the present work, we provide a comprehensive analysis of naturally produced GAS MVs and assess the involvement of genetic factors influencing MV production in GAS.

## RESULTS

### GAS exhibits active MV production during *in vitro* growth.

**FIG 1:**
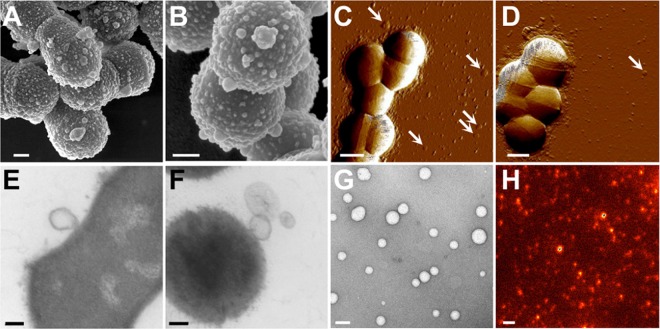
GAS cells exhibit MV protrusions during *in vitro* growth. Cellular morphologies of ISS3348 (A, C, and E) and SF370 (B, D, and F) were examined by SEM (A and B), AFM (C and D), and TEM (E and F). Isolated MVs from ISS3348 were visualized using negative-stain TEM (G) and staining for M1 protein and STED microscopy (H). White arrowheads indicate vesicle-like structures. Scale bars are drawn to 200 nm (A and B and E to G) or 500 nm (C, D, and H).

We first undertook morphological examination of GAS clinical isolate ISS3348 and reference strain SF370 (both M1 serotype) axenic cultures using several high-resolution microscopy approaches. Both strains displayed multiple spherical structures protruding from their cell membranes, with diameters ranging between 10 and 272 nm (Fig. 1A and B). These vesicle-like structures appeared liable to be released from the membrane, as similarly sized vesicular structures could also be discerned both on the cell surface and in the vicinity of the streptococci (Fig. 1C to F). To determine whether these structures were indeed freely released from the membrane, GAS culture supernatants were filtered twice through 0.22-µm-pore membranes prior to ultracentrifugation. Pellets obtained from ISS3348 cultures were examined by negative-staining transmission electron microscopy (TEM) and exhibited a heterogeneously sized population of circular structures suggestive of extracellular membrane-derived vesicles (MVs) (Fig. 1G; see Fig. S1A in the supplemental material). To determine whether MV production is an active process, MVs from late-logarithmic-phase GAS culture supernatants and the same cultures incubated in fresh media following heat inactivation were quantified by flow cytometry (see Fig. S1B). MV production of both cultures following heat inactivation was reduced to the level of flow cytometric events recorded with buffer alone; interestingly, ISS3348 demonstrated significantly higher MV production than SF370 (see Fig. S1B). These data support the assumption that production of MVs is an active process requiring live, growing bacteria. MVs were also isolated from GAS treated with a sublethal concentration of lysozyme, leading to an altered MV protein profile in lysozyme-treated bacteria (see Fig. S1C). An altered MV protein profile in lysozyme-treated bacteria indicates that weakening of the cell wall via lysozyme treatment releases membrane fragments distinct from natively isolated MVs as has been previously noted (17), refuting the possibility that MVs are formed through nonspecific assembly of shed membrane fragments.

As a complementary approach, ISS3348 MVs were visualized by immunofluorescent staining of M1 protein using stimulated emission depletion (STED) microscopy (Fig. 1H). STED microscopic analysis permitted the accurate resolution and measurement of MV particles (Fig. 1H; see Fig. S1D in the supplemental material), with these data supporting previous TEM image analysis (see Fig. S1A). No fluorescent labeling in STED images was noted in the absence of MV samples, and as such, particle measurements were unlikely to result from freely reactive dye molecules (see Fig. S1E to G). Size distribution measurements were further confirmed by dynamic laser light scattering (DLS), with isolated MVs from ISS3348 and SF370 exhibiting single, broadly dispersed populations (see Fig. S1H). Taken together, these data strongly support the notion that GAS actively produces MVs during normal cell growth. As the clinical isolate ISS3348 produced more MVs relative to other strains, we focused primarily on this isolate for further characterization of MV composition.

### Proteomic characterization of GAS MVs.

**FIG 2:**
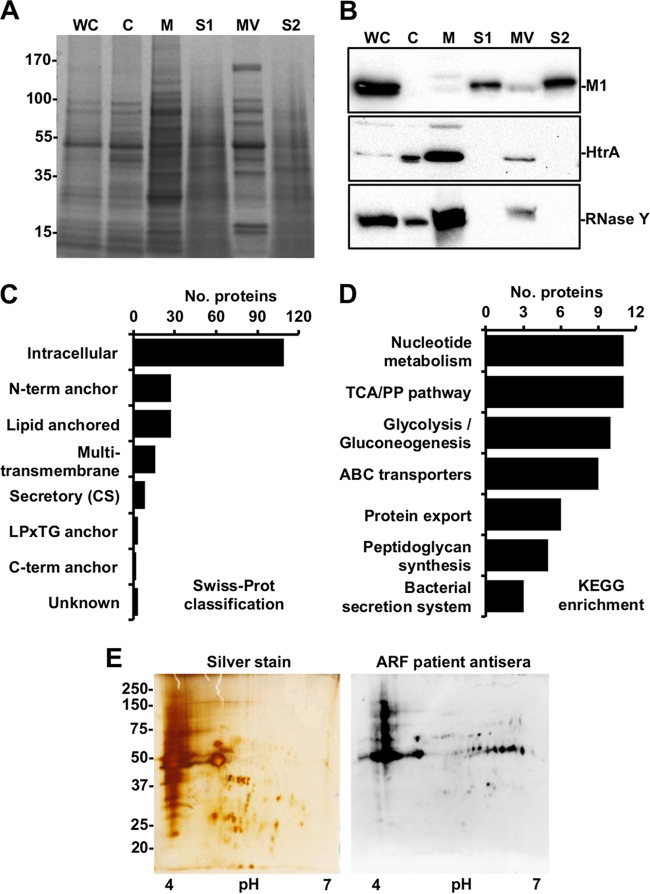
GAS MVs carry classically secreted and anchorless virulence proteins. (A) Protein profiles of ISS3348 whole-cell extract (WC), cytoplasm (C), membrane (M), secreted fraction prior to ultracentrifugation (S1), MV, and secreted fraction after centrifugation (S2). (B) Western blot of virulence-associated proteins identified in MVs. The samples in each lane are the same as in panel A. (C) Swiss-Prot classification of nano-LC-MS/MS-identified MV proteins. (D) KEGG categories enriched in MV proteins. (E) Silver-stained 2D protein profile and respective 2D Western blot of ISS3348 MV proteins probed with ARF patient antisera and anti-human IgG-Fc-horseradish peroxidase (HRP).

Previous studies of diverse Gram-negative bacteria have indicated that outer membrane proteins are differentially enriched in OMVs relative to the outer membrane (18–20). Whether Gram-positive bacteria, which lack an outer membrane, differentially enrich proteins in MVs relative to the bacterial membrane is unknown. We isolated ISS3348 subcellular fractions and compared total protein profiles by one-dimensional (1D) SDS-PAGE (Fig. 2A). In comparison to the secreted and membrane fractions, MV protein profiles exhibited similarities to and differences from both compartments (Fig. 2A), a phenomenon also observed in protein fractions from SF370 and other M1 GAS strains (see Fig. S2A in the supplemental material). Further complementary proteomic analysis by 2D difference gel electrophoresis (DIGE) and high-resolution nano-liquid chromatography-tandem mass spectrometry (nano-LC-MS/MS) analyses revealed a high degree of complexity and overlap between GAS MVs and membranes (see Fig. S2B). We consistently identified 905 proteins in MVs and 1,027 proteins in membranes, of which 23 proteins were more prominently found in MVs compared to membranes according to our filter criteria and 3 proteins were entirely unique to MVs (see Fig. S2C and Data Set S1 in the supplemental material). Label-free quantification revealed 169 proteins that were >2-fold enriched in MVs relative to membranes (see Table S1 in the supplemental material), resulting in a consensus MV proteome of 195 proteins. The subcellular distribution of a subset of proteins identified in the MV proteome, included M1 protein, serine protease HtrA, and RNase Y, the presence of which was confirmed by immunoblotting (Fig. 2B). Grouping of MV proteins by putative location indicated that more than half were cytoplasmic, with the next largest group being putatively membrane associated (Fig. 2C). Furthermore, we tested for enrichment of MV proteins involved in discrete biological processes, revealing that a number of proteins in seven KEGG categories were statistically overrepresented (*P* < 0.0001; false discovery rate [FDR], <5%), including the nucleotide metabolism, glycolysis/gluconeogenesis, tricarboxylic acid/pentose phosphate (TCA/PP) pathway, ABC transports, protein export, peptidoglycan synthesis, and bacterial secretion (Fig. 2D). A complement of virulence-associated proteins, including M protein, C5a peptidase (ScpA), and streptolysin O were identified in both MVs and the membrane fraction (see Fig. S2D).

In comparison with previous studies, we identified MV proteins arising from both the GAS secretome and the cell surface (13, 21) (see Fig. S2E and F and Data Set S1 in the supplemental material). A number of the identified MV proteins have recently been proposed as candidates of a non-M protein-based prophylactic vaccine (22) (see Fig. S2G). To examine the immunogenicity and pathological relevance of MV proteins, we immunoblotted vesicle samples after 2D SDS-PAGE and probed with pooled antisera from patients suffering from acute rheumatic fever (ARF) (Fig. 2E). The presence of multiple immunoreactive IgG epitopes in MV preparations suggests that protein factors present in MVs are expressed during GAS infection in humans.

### Lipid components of GAS MVs.

**FIG 3:**
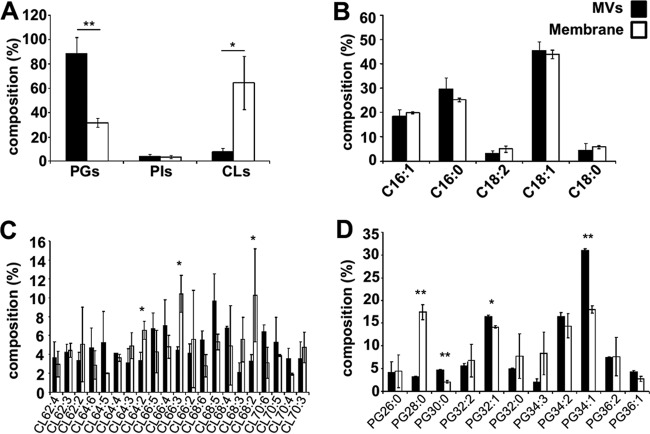
PG is the dominant anionic phospholipid in GAS MVs. (A) Characterization of anionic phospholipids phosphatidylglycerol (PG), phosphatidylinositol (PI), and cardiolipin (CL) in ISS3348 MVs and membranes. (B) General distribution of GAS medium-chain fatty acids (FAs) in MVs and membranes. (C and D) Comparison of GAS MVs and membrane FA saturation level and arrangement in anionic lipids CL and PG. The results presented are means ± standard deviations (SD) from 3 experiments. Asterisks indicate statistical significance by one-way analysis of variance (ANOVA) with Tukey’s *post hoc* test: *, *P* < 0.05; **, *P* < 0.01.

To further characterize GAS MV composition, the lipid components of ISS3348 cell membranes and MVs were analyzed using a previously described matrix-assisted laser desorption ionization (MALDI)-based mass spectrometry approach (see Fig. S3A to C in the supplemental material) (23). More than 85 individual lipid species corresponding to glycoglycerolipids and both anionic and cationic phospholipids (PLs) could be identified. These lipid classes primarily contained 16:1 (palmitoleic), 16:0 (palmitic), 18:1 (oleic/vaccenic), 18:2 (linoleic), and 18:0 (stearic) fatty acids (FAs) (see Table S2 in the supplemental material). Thin-layer chromatography (TLC)-based quantification of membrane and MV lipid species identified anionic phosphatidylglycerol (PG) as being significantly enriched in GAS MVs (Fig. 3A; see Fig. S3D). In contrast, cardiolipin (CL) comprised only a minor fraction of MV lipids relative to the GAS membrane (Fig. 3A). Other lipid classes, including glycoglycerolipids and cationic PLs, were found to be equally distributed between MVs and the GAS membrane (see Fig. S3E and F). Although the overall saturation levels of medium-chain (C_16_ and C_18_) FAs were not different between MVs and membranes (Fig. 3B), characteristic differences in the FA distributions of individual CL and PG species (Fig. 3C and D) and other less abundant lipids were observed (see Fig. S3G and H). Differential enrichment of MV lipid species relative to the membrane provides further evidence for the existence of an ordered mechanism contributing to MV biogenesis, as opposed to nonspecific membrane shedding, which would generate MVs with an identical lipid composition.

### GAS MVs contain differentially abundant RNA species.

Packaging of DNA into OMVs has been noted historically as a mechanism facilitating horizontal transfer of antibiotic resistance genes between Gram-negative bacteria (24–26). Recent studies also report that RNA molecules are present in OMVs in marine ecosystems and in pathogenic *Vibrio cholerae* OMVs (27–29). We initially examined ISS3348 MVs for binding of fluorescent nucleic acid dyes following rigorous nuclease treatment (see Fig. S4A in the supplemental material). MVs were negative for the membrane-impermeable DNA/RNA dye propidium iodide; however, they were positive for a membrane-permeable RNA-specific dye (see Fig. S4A), indicating that GAS may secrete RNA species protected in the lumen of MVs.

**FIG 4:**
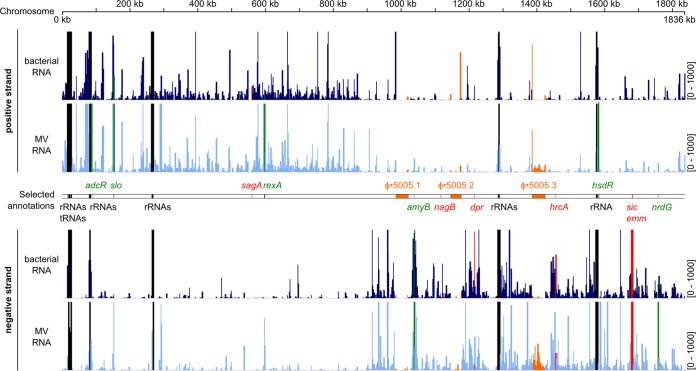
RNA species are differentially abundant between GAS cells and MVs. Coverage of reads from MVs and bacterial cellular RNAs mapped to the reference M5005 genome. Chromosomal coordinates are indicated at the top in kilobases. The scale for mapped reads is indicated to the right of the coverage map. tRNA and rRNA loci are indicated in black. The positions of prophage regions are indicated in orange. Selected RNA species more abundant in MVs are highlighted in green, and RNA species less abundant in MVs are highlighted in red. The vesicular and bacterial coverages shown are from a representative sample from biological triplicates.

We characterized RNA species present in MVs using deep RNA sequencing (RNA-seq), comparing the abundance of vesicular RNAs relative to bacterial cellular RNAs (Fig. 4). Both bacterial and MV-associated mapped reads were well distributed over the reference genome (Fig. 4). The majority of vesicular and bacterial sequences obtained corresponded to rRNAs and tRNAs, with approximately 14% of MV RNAs sequenced mapping uniquely to loci in the reference genome, many of which were differentially abundant between MVs and bacteria. At a conservative cutoff (≥log_2_ fold change, 1.5; adjusted *P* value, <0.01), a total of 207 RNA species were found to be differentially abundant in MVs relative to bacteria (see Table S3 in the supplemental material). Of these, 120 RNAs were more abundant in MVs, with 47 of the 50 most highly differentially abundant RNAs being more enriched in the MV fraction. Highly abundant MV RNAs mapped within the zinc-responsive adhesive competence repressor *adcR*, anaerobic ribonucleoside triphosphate reductase *nrdG*, exonuclease-helicase subunit A *rexA*, and type 1 restriction endonuclease *hsdR* genes (see Fig. S4B in the supplemental material). Conversely, 87 RNAs were less abundant in the MV fraction, including streptolysin S gene *sagA* and glucosamine-6-phosphate isomerase gene *nagB* (see Fig. S4B).

We further analyzed a number of differentially abundant vesicular RNAs by quantitative PCR (qPCR) (see Fig. S4C in the supplemental material). Seven more abundant (*adcR*, *rexA*, *nrdG*, *amyB*, *salR*, *artP*, and *slo*) and three less abundant (*sagA*, *nagB*, and *pfl*) vesicular RNA species were quantified by qPCR from triplicate samples isolated independently from those used for transcriptome sequencing. qPCR data obtained demonstrated a strong correlation with the RNA-seq fold change values (Pearson correlation, *R* = 0.977; *P* < 0.0001). Both RNA-seq and qPCR analyses strongly indicate that GAS MVs contain RNA species that are differentially abundant from the originating bacteria. Previous studies have shown that *Escherichia coli* OMVs contain abundant tRNAs and small, noncoding RNA species (29). To our knowledge, this is the first report describing Gram-positive bacterial secretion of differentially enriched intragenic RNAs via extracellular membrane-derived vesicles.

### GAS vesiculogenesis is affected by activity of the two-component regulator CovRS.

**FIG 5:**
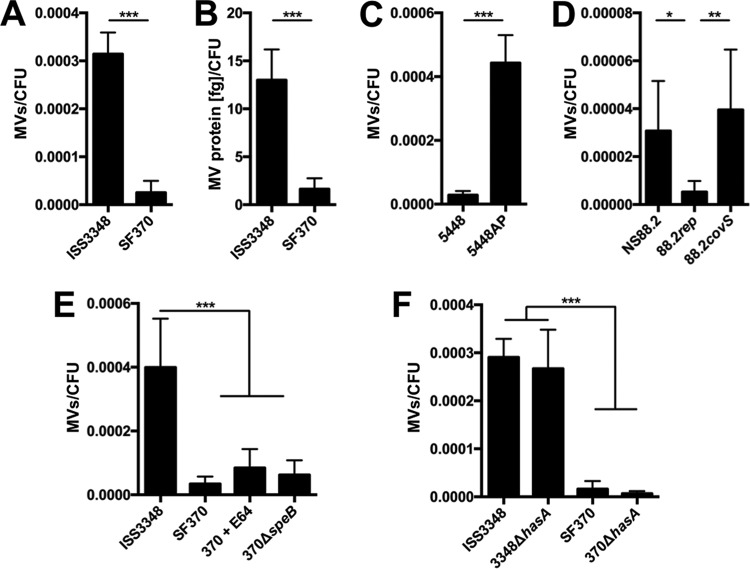
CovRS signaling impacts MV production. Shown is quantification of MV production by GAS strains on the basis of FM1-43 dye staining expressed as MVs per CFU (A and C to F) or total vesicular protein abundance estimated by Bradford protein determination expressed as femtograms of MV protein per CFU (B). Asterisks indicate statistical significance by Student’s unpaired *t* test (A to C) or one-way ANOVA with Tukey’s *post hoc* test (D to F): *, *P* < 0.05; **, *P* < 0.01; and, ***, *P* < 0.001. The results presented are means ± SD from 3 to 5 independent experiments, each of which was conducted with 2 technical replicates.

During our characterization of GAS MV production and composition, we noted a consistently higher quantity of recoverable MVs from ISS3348 culture than from SF370 culture. This difference was significant and reproducible using two different methodologies: (i) flow cytometric counting of MV particles and (ii) total MV protein abundance (Fig. 5A and B) (*P* < 0.001). Of note, both methodologies demonstrated a high correlation in the quantification of MV production (see Fig. S4D in the supplemental material). We hypothesized that the genomic backgrounds of these strains influenced their level of MV production. ISS3348 has been described to bear inactivating mutations in the two-component “control of virulence regulator-sensor” operon (*covRS*) (30). This finding was verified by direct sequencing of the ISS3348 *covRS* operon, which exhibited a large deletion in the *covS* (sensor) catalytic domain (*covS*_Δ1262–1306_ of SF370 sequence). Inactivating mutations in *covRS* are frequently acquired by M1 strains and result in broad regulatory alterations (31). Accordingly, we found several virulence-associated, *covRS*-regulated genes (*hasA*, *slo*, *speB*, and *grab*) differentially expressed in ISS3348 relative to SF370 (see Fig. S4E), suggesting that this mutation disrupts CovS function and may be responsible for the increased virulence of this strain in animal infection models (30).

Recent evolutionary tracing of the M1 serotype GAS genomic lineage illustrates that the laboratory reference clinical isolate SF370 differs significantly in both mobile and core genomic compositions from more contemporary M1 isolates, including ISS3348 (32). To distinguish whether the differences in the genomic contents of ISS3348 and SF370 or disruption of *covRS* signaling influences MV formation, MVs were isolated from M1 GAS strains 5448 and 5448AP. These strains carry identical genomes, except for a single base pair insertion in *covS* leading to expression of truncated, inactive CovS protein in the 5448AP strain (33). We show that the 5448AP strain produces a significantly higher number of MVs relative to 5448 (Fig. 5C). This finding suggests that genes under control of the CovRS regulator may affect GAS MV production. To examine whether this phenomenon applied exclusively to M1 GAS, we analyzed the virulent *covS*-deficient M98.1 GAS strain NS88.2, *covS*-intact derivative 88.2 *rep*, and complemented strain 88.2 *covS* (34). Consistent with a model of *covRS*-mediated regulation of MV production, *covS*-defective strains NS88.2 and 88.2 *covS* exhibited significantly higher MV production, albeit with a smaller degree of difference in comparison to M1 *covRS* mutant strains (Fig. 5D). Altogether, the increased MV production by strains harboring defective *covRS* loci supports the conclusion that the CovRS two-component system impacts MV-mediated release of proteins, nucleic acids, and lipids.

### GAS MV production is independent of SpeB activity and capsule synthesis.

Previous studies demonstrate that extracellular cysteine protease SpeB drastically remodels the GAS secretome in response to *covRS* mutations (35). Consistent with the strong downregulation of *speB* by *covRS*-deficient strain ISS3348 relative to SF370 (see Fig. S4E in the supplemental material), this strain exhibited extracellular cysteine protease activity equivalent to that of an SF370 SpeB deletion strain (370 Δ*speB*) and SF370 culture supernatant incubated with cysteine protease inhibitor E64 (see Fig. S4F). To determine whether an absence of extracellular SpeB activity resulted in a larger quantity of recoverable MVs, MVs were isolated from 370 Δ*speB* and SF370 grown in the presence of E64 (Fig. 5E). No significant differences in MV production between SF370 and SpeB activity-deficient conditions were observed (Fig. 5E), suggesting that GAS MV production is insensitive to SpeB-mediated degradation of the soluble GAS secretome.

Capsule has previously been suggested to hinder *S. pneumoniae* MV release (12). In concordance with previous studies (31), mutation of *covRS* in ISS3348 resulted in upregulation of expression of the hyaluronic acid capsule biosynthesis gene *hasA* (see Fig. S4E). To assess whether capsule expression impacted GAS MV release, MVs were isolated from capsule-deficient ISS3348 (3348 Δ*hasA*) and SF370 (370 Δ*hasA*) mutants (Fig. 5F). The 3348 Δ*hasA* and 370 Δ*hasA* strains showed no significant differences in MV production relative to the parental strains (Fig. 5F). Thus, although MV production appears directly or indirectly regulated by CovRS activity, two phenotypically important genes under direct control of *covRS*, *hasA* and *speB*, do not impact GAS vesiculogenesis.

## DISCUSSION

Vesicular packaging of biomolecules enables cellular compartmentalization and export of proteins, nucleic acids, and lipids in eukaryotes. This mechanism is also utilized by Gram-negative bacteria and has been the subject of intense study (9). Until recently, MV production by Gram-positive bacteria has been disregarded, due to precluding differences in cell wall structure (16). Here, we present a comprehensive analysis of the protein, nucleic acid, and lipid composition of GAS MVs and provide evidence for a two-component system regulating MV release.

To characterize MV protein cargo and examine potential specific enrichment of vesicular versus membrane proteins, we undertook both 2D electrophoretic and mass spectrometric approaches. Both analyses demonstrated a high overlap in protein content and enrichment of MV proteins relative to the membrane. Similar findings have been reported for outer membrane proteins in Gram-negative bacteria (18–20); however, a global comparative analysis of protein enrichment in Gram-positive MVs has been lacking. Multiple putatively soluble (intracellular and secreted) proteins were identified in the membrane fraction, as previously noted (6, 7). A limitation of this study is that despite identification of putatively soluble proteins in membrane, the true localization of anchorless surface proteins on a global scale is difficult to predict. We calculated enrichment of putatively soluble MV-associated factors relative to the membrane compartment; however, the intracellular concentrations of these factors may lead to differences in enrichment values relative to whole-cell concentrations. Of the enriched MV factors, we identified multiple virulence-associated and metabolic proteins. Mechanistic explanation of how this enrichment occurs should be the focus of further investigation.

GAS protein secretion has been linked to a distinct lipid microdomain, the ExPortal, which is enriched in anionic phospholipids PG and CL and serves as a cue for localization and accumulation of Sec pathway translocons (5). We therefore performed lipidomic analysis to link cellular lipid asymmetry to GAS vesiculogenesis and Sec-independent secretion via ExPortal-like microdomains. Our analysis demonstrates a selective release of PG over CL in MVs compared to whole bacterial membranes. Detailed lipidomic analysis revealed characteristic differences in the compositions and distributions of individual lipid species and differences in the chain lengths/saturation levels of FAs between MVs and the GAS membrane. Based on these results, we hypothesize that enrichment of PG relative to CL, a factor known to dictate membrane curvature (36), and increased monounsaturated FA content in MV lipid classes could determine GAS MV formation and export of cargo.

Why and how MVs containing asymmetrical RNA species relative to the bacteria arise are unclear. It is feasible that bacterial subpopulations with distinct transcriptomic profiles are more prone to produce MVs and contribute to the differential enrichment of MV RNAs relative to the total bacterial population. This hypothesis is supported by recent evidence of a subpopulation of *Pseudomonas aeruginosa* strains that contribute to MV formation via explosive cell lysis (37). In comparison to previous studies, we found a high degree of intragenic RNA species, as opposed to intergenic or tRNA species, differentially enriched in MVs relative to bacterial cells (29). Transcription factor *adcR* mRNA was highly enriched in GAS MV fractions and recently was shown to play a central role in GAS adaptive responses to zinc availability (38). Thus, secretion of selected RNAs may allow rapid intercellular communication analogous to secretion of mammalian exosomal shuttle RNA (39).

Most bacterial species express alternative transcription factors (sigma factors [σ]) that direct transcription to distinct gene subsets, enabling adaptation to changing conditions. Multiple reports indicate OMV production is increased in response to defects in the σ^E^ pathway, whereby OMV formation enables cells to eject toxic accumulations of misfolded periplasmic proteins (40). This mechanism may also apply in the case of general stress-responsive σ^B^ present in Gram-positive bacteria (41). The CovRS system in GAS acts as a global regulator of approximately 15% of the GAS genome, including many known GAS virulence determinants, in response to general stress conditions (42, 43). GAS does not encode recognizable stress response sigma factors; however, CovRS has been hypothesized to act as a functional analogue of these transcription factors (43). The observation that disruption of *E. coli* EnvZ/OmpR response regulator (to which CovRS is homologous) similarly bestows a hypervesiculation phenotype supports our finding that CovRS signaling impacts MV production in GAS (44). This finding is further emphasized by the recent observation of vesicle-like structures on the GAS cell surface following incubation with CovS ligands LL-37 and RI-10 (14). Collectively these data could indicate a general trend in regulation of MV synthesis through two-component regulators, particularly in the absence of alternative sigma factors.

Our finding that GAS MV production is unaffected by SpeB protease activity is noteworthy, given that this factor effects broad proteolytic degradation of the GAS secretome and is directly regulated by *covRS* (35). It is also feasible that SpeB-expressing GAS have an altered MV protein composition. Transcriptomic profiles of *covRS*-defective M1 GAS have previously been examined and implicate a multitude of other regulators and virulence factors as potential contributors to MV production (31). Whether one factor solely contributes or multiple *covRS*-regulated factors act in concert to influence MV biogenesis is unknown. In an analogous study of *Bacillus subtilis* extracellular membrane-derived vesicles, it was determined that differences in recoverable MV quantities between an environmental strain and laboratory strain were linked to the secretion of lipopeptide antibiotic surfactin, which destabilized MV integrity (45). It is possible that a *covRS*-regulated factor acts in a similar manner to either disrupt MV integrity or inhibit MV release. This notion is supported by the morphological evidence of MV formation on the SF370 strain cell surface, despite lower quantifiable MV yield.

During the preparation of the manuscript for this article, Biagini et al. (13) demonstrated that GAS releases high-molecular-weight vesicular structures principally composed of lipoproteins. In agreement with their proteomic analysis, we identified all except two (M5005_Spy0249 and M5005_Spy_1732) abundant vesicular lipoproteins found in reference 13 in our 60 highest-label-free quantification (LFQ)-value MV proteome, as well as 80 out of the 83 vesicular non-lipoproteins. (M5005_Spy0012, M5005_Spy1434, and M5005_Spy0107 were not found in our study.) We furthermore consistently identified 50 of 55 proteins detected in the ISS3348 chemically defined media secretome from reference 13 in our MV preparations (see Fig. S2E in the supplemental material). (M5005_Spy0249, M5005_Spy0996, M5005_Spy0469, M5005_Spy2009, and M5005_Spy1436 were not found in our study.) The MV lipidomic analyses of both our study and the study by Biagini et al. (13) indicate an asymmetrical relationship between the vesicular and overall membrane lipid composition, corroborating our identification of the MV-associated ExPortal microdomain proteins and lipid components. It should be noted that despite weakening the GAS cell wall using sublethal penicillin treatment, Biagini et al. (13) noted no significant changes in either the MV proteome or membrane lipidome.

Collectively, we propose GAS exports differentially enriched RNA species and proteins lacking traditional secretion signal peptides through production of MVs of a characteristic lipid composition. These findings should stimulate further studies of MV biogenesis mechanisms and elucidation of the downstream effects of MV-borne factors on both neighboring bacteria and the human host during GAS infection.

## MATERIALS AND METHODS

For detailed methodologies, see the Text S1 in the supplemental material.

### Bacterial strains.

GAS strains used in this study (see Table S4 in the supplemental material) were routinely cultured at 37°C in 5% CO_2_ on tryptone soy agar (TSA) sheep blood agar or in Todd-Hewitt broth (THB) without agitation. Construction of the 370 Δ*speB* and 3348 Δ*hasA* strains is described in detail in Text S1 in the supplemental material.

### MV isolation and quantification.

MVs were routinely isolated from late-logarithmic- or early-stationary-phase GAS cultures in THB. Bacteria were pelleted at 2,500 × *g*, and the supernatant was decanted and filtered twice through 0.22-µm-pore polyethylsulfone membranes prior to ultracentrifugation (175,000 × *g*, 4 h, 4°C). Pellets obtained after ultracentrifugation were directly quantified or were washed once in phosphate-buffered saline (PBS) and further processed for other analyses (described below). MVs were quantified by Bradford protein determination assay (Sigma) or by incubation with 0.5 µg/ml of lipophilic membrane dye FM1-43 (Life Technologies) for 15 min, prior to flow cytometric counting using Count Bright counting particles (Life Technologies). MV production was normalized to the CFU of each strain. For detection of MV RNA species, MVs and GAS were incubated with Benzonase (see the protocol for vesicular RNA isolation below) and stained with both 0.5 µg/ml of FM4-64 and 1 µM Syto RNAselect dyes (Life Technologies) or with propidium iodide (BioLegend) for 15 min prior to detection by flow cytometry.

### AFM and STED microscopic analysis.

Atomic force microscopy (AFM) of bacteria was performed as previously described (46). STED microscopy was conducted by blocking MVs using naive rabbit serum, followed by labeling with mouse anti-M1 antiserum, washing once in PBS, and secondary labeling with goat anti-mouse Alexa Fluor 488 IgG (Dako). Measurements of labeled MVs were taken using a home-built STED microscope setup previously described (47). Data acquisition and analysis was performed using Imspector software (Imspector Image Acquisition and Analysis software v0.10; http://www.imspector.de).

### Proteomics.

Delipidized protein samples with disulfide bonds reduced and thiol groups alkylated were trypsinized and further processed for nano-LC-MS/MS using the Q Exactive Orbitrap mass spectrometer (Thermo Fisher). Raw data were analyzed using MaxQuant, with protein localization and function predicted using LocateP and DAVID.

### Lipidomic analysis.

Lipids extracted with methanol were profiled using FlexiMass-DS sample plates (Shimadzu). Mass spectra were recorded using an AXIMA-CFRplus (Shimadzu) curved-field reflectron time-of-flight mass spectrometer in positive or negative mode using delayed ion extraction for unit mass resolution.

### Vesicular RNA isolation and RNA-seq.

Following MV isolation from early-stationary-phase GAS cultures, samples were treated with Benzonase nuclease (Sigma) for 30 min to degrade all forms of extracellular nucleic acids prior to being washed once with PBS. Vesicular RNA was isolated from biological triplicates using TRI reagent (Sigma), followed by treatment with DNA-free Turbo DNase (Ambion) to remove any contaminating genomic DNA (gDNA). The RNA concentration was assessed using a Bioanalyzer (Agilent) prior to library construction. cDNA libraries were generated without rRNA depletion using the ScriptSeq v2 library preparation kit (Epicentre) and sequenced on an Illumina HiSeq 2500 platform. Short or low-quality reads were filtered and trimmed prior to mapping of trimmed reads to the most closely related reference genome (MGAS5005; GenBank accession no. NC_007297) using the STAR alignment tool (48). Visualization and manual inspection of read coverage were conducted using the Integrative Genomics Viewer (IGV) (49). Differentially abundant RNAs in MV samples and bacteria were computed using the DESeq2 package (50). Prophage-associated genes (φ5005.1, M5005_0995 to -M5005_1054, φ5005.2, M5005_1168 to M5005_1222; and φ5005.3, M5005_1414 to -M5005_1467) were removed from differential expression results.

### Accession number(s).

RNA sequencing data have been deposited at NCBI under accession no. SRP089791.

Mass spectrometry proteomics data have been deposited to the ProteomeXchange Consortium (http://proteomecentral.proteomexchange.org) via the PRIDE partner repository with the dataset identifier PXD005167.

## SUPPLEMENTAL MATERIAL

Text S1  Supplemental materials and methods. Download Text S1, DOCX file, 0.1 MB

Data Set S1  Detailed proteomic analysis of GAS MVs and membranes by nano-LC-MS/MS. Shown is a comprehensive list of annotated proteins identified by at least 2 peptides in GAS ISS3348 MV or respective membranes in any of the biological triplicates. Download Data Set S1, XLSX file, 0.5 MB

Figure S1  Biophysical analyses of GAS MVs. (A) Size distribution of isolated ISS3348 MVs as determined by ImageJ-based vesicle diameter measurements taken from negative-stain TEM micrographs (*n* = 147). Cumulative size frequency is given on the right *y* axis. (B) Quantification of MV production by ISS3348 and SF370 on the basis of FM1-43 dye staining pre- and post-heat inactivation (HI). MVs were harvested from late-logarithmic-growth-phase culture supernatants, purified, and quantified as described in Materials and Methods in the main text. GAS cultures were subsequently washed twice in PBS and heat inactivated at 80°C for 1 h, prior to inoculation into fresh media for 4 h, with MVs purified and quantified from HI GAS cultures as described in Materials and Methods in the main text. The results presented are pooled data ± SD from two independent experiments. Asterisks indicate statistical significance by one-way ANOVA with Tukey’s *post hoc* test: ***, *P* < 0.0001. (C) Treatment of mid-logarithmic-growth-phase ISS3348 and 5448AP cultures with or without 1 mg/ml lysozyme for 2 h prior to MV isolation. Cultures were lysozyme treated prior to MV isolation and SDS-PAGE analysis. (D) Size distribution of FM1-43-labeled ISS3348 MVs as determined by the full-width half-maximum (FWHM) profile of pixel intensities of individual anti-M1-stained MVs using stimulated emission depletion (STED) microscopy. (E and F) STED analysis of isolated ISS3348 MVs stained with mouse anti-M1 and anti-mouse Alexa Fluor 488 (E) or Alexa Fluor 488 alone (no MVs) (F). (G) Confocal microscopy analysis of isolated ISS3348 MVs stained with anti-M1 and anti-mouse Alexa Fluor 488. (H) Size distribution of ISS3348 and SF370 MVs assessed by dynamic laser light scattering analysis. Download Figure S1, TIF file, 1 MB

Figure S2  GAS MV and membrane protein profiles. (A) Protein profiles of cytoplasmic (C), membrane (M), and MV (MV) samples from strains 5448, 5448AP, and SF370. (B) Representative 2D DIGE images from Cy5-labeled ISS3348 MVs and respective Cy3-labeled membranes. Individual channels are shown in black and white, and the merged image was created in ImageJ. (C) Venn diagram showing the overlap of proteins identified in ISS3348 MVs and membranes by at least 2 peptides in each biological triplicate using nano-LC-MS/MS. (D) Venn diagram showing the overlap between virulence-associated proteins (http://www.iedb.org and http://www.mgc.ac.cn/VFs), proteins identified in ISS3348 MVs (unique and >2-fold-enriched compared to membranes [MV+M]; see Data Set S1 in the supplemental material), and all identified proteins in membrane and MV analysis (ALL [see Data Set S1]). (E) Venn diagram showing the overlap of recently published (13) secreted and ISS3348 MV proteins with proteins identified in this study. (F) Venn diagram showing the overlap of surface-associated proteins (21) with proteins identified in this study. (G) Venn diagram showing the overlap of immunogenic proteins described by Fritzer et al. (22) and proteins identified in this study. (H) Representative Coomassie-stained ISS3348 MV protein profile and immunoreactive MV protein profile after incubation with ARF patient antiserum. Download Figure S2, TIF file, 1 MB

Figure S3  Supporting GAS MV and membrane lipidomic data. (A to C) Representative annotated ionization/mass-spectrometry spectra of different lipid classes showing mono- and diglycoglycerolipids (MG and DG, respectively [A]), anionic phospholipids (PG, CL, and PI [B]), and cationic phospholipids (PC [C]). (D) Representative TLC of lipid extracts from ISS3348 GAS MVs and corresponding bacterial membrane preparations (M). Sample loading was normalized to MV protein abundance from Coomassie staining. (E) Quantitative distribution of mono- and diglycosyldiacylglycerols (MGDG and DGDG, respectively) in GAS MVs and membranes. (F) Quantitative distribution of sphingomyelin (SM) and phosphatidylcholine (PC) in GAS MVs and membranes. (G and H) Acyl chain length and saturation level in MGDG (G) and PC (H). Download Figure S3, TIF file, 1.1 MB

Figure S4  Fluorescence-activated cell sorter (FACS) and qPCR analysis of GAS MV RNA. (A) Analysis of ISS3348 MV nucleic acid content by flow cytometry. MVs were isolated as described for RNA-seq analysis and incubated with propidium iodide (PI), singly with 0.5 µg/ml of the lipophilic dye FM4-64 (Life Technologies) or with FM4-64 together with 1 µM of Syto RNAselect (Life Technologies) in the dark for 15 min, prior to being washed once in PBS and analysis of 30,000 events by flow cytometry. Negative controls consisting of PBS plus nucleic acid dyes alone had minimal fluorescence (not shown). Fluorescence of stained MVs or GAS (blue) is shown relative to that of unstained samples (top and bottom rows) or samples stained singly with FM4-64 (red) (middle row). The data shown are representative of two experiments with similar results. (B) Reads per kilobase of transcript per million mapped reads (RPKM) values for selected differentially abundant RNA species. RNA species more abundant in MVs are shown in the right panel and RNA species more abundant in bacteria in the left panel. The results presented are means ± SD. (C) Correlation of differentially abundant RNA species by RNA-seq and qPCR analyses. The Pearson’s correlation coefficient *R*, *P* value, and *R*^2^ of the linear regression line are indicated. Differences in RNA abundance determined by the DESeq2 algorithm and qPCR were log transformed and are expressed as fold change in MV RNA relative to bacterial RNA. The results presented are pooled means from independently triplicated experiments. (D) Correlation of FM1-43-based flow cytometric counting and total vesicular protein abundance quantification using Bradford protein determination. MV abundance is expressed as number of FM1-43-positive events per CFU or femtograms of MV protein per CFU. The Pearson’s correlation coefficient *R* and *P* value are indicated. (E) Expression of ISS3348 *covRS*-regulated genes relative to SF370 during the late logarithmic growth phase. The results presented are pooled means ± SD from two independent experiments conducted in triplicate. (F) Cysteine protease activity of GAS supernatants as assessed by azocasein degradation. The results presented are pooled means ± SD from three independent experiments. Download Figure S4, TIF file, 0.5 MB

Table S1  Proteins exclusively present or enriched in GAS MVs. Table S1, DOCX file, 0.2 MB

Table S2  Detailed MALDI-MS lipidomic analysis of ISS3348 MVs and membranes. Table S2, DOCX file, 0.1 MB

Table S3  DESeq2 analysis of GAS MV and bacterial cellular RNAs. Table S3, DOCX file, 0.4 MB

Table S4  Strains and primers used in this study. Table S4, DOCX file, 0.1 MB
