# Supplementary material for: A Two-Component Regulatory System Impacts Extracellular Membrane-Derived Vesicle Production in Group A Streptococcus
Source: mBio. 2016 Nov 1;7(6):e00207-16. doi: 10.1128/mBio.00207-16 (PMC5090034; doi:10.1128/mBio.00207-16)
Supplement: Text S1 — Supplemental materials and methods. Download [file mbo005163043s1.docx]

**Supplemental Materials and Methods**

*Construction of 370Δ*speB *and 3348Δ*hasA

Complete deletion of the *speB* gene in SF370 was performed using the temperature-sensitive shuttle vector pRDN18 as described previously (1). Briefly, an upstream fragment (with BamHI and KpnI sites added by 5’ primer modification, OLEC114 and OLEC115, respectively), and downstream fragment (with KpnI and EcoRI sites added by 5’ primer modification, OLEC116 and OLEC117, respectively) of the *speB* coding sequence were amplified by PCR. The downstream fragment was then cloned into pRDN18 using the KpnI and EcoRI sites, sequentially, followed by cloning of the upstream fragment using the BamHI and KpnI sites. The resulting plasmid was transformed into SF370 at the permissive temperature of 28°C, prior to shifting to the restrictive temperature of 40°C in the presence of spectinomycin to promote single crossover integration into the chromosome. Passage of Spec^R^ colonies at 28°C in the absence of spectinomycin permitted a double crossover event, resulting in the loss of the original vector and complete removal of *speB* coding sequence. Complete deletion of ISS3348 *hasA* was performed using Cre-Lox recombination. Briefly, an erythromycin resistance (Erm^R^) cassette flanked by the lox71 and lox66 sites (OLEC1931 and OLEC1932), upstream fragment (OLEC2712, OLEC2713) and downstream fragment (OLEC2714, OLEC2715) of *hasA* coding sequence were amplified by PCR. All three fragments were combined by nested PCR using OLEC2716 and OLEC2717, prior to cloning into pJET1.2. The resulting plasmid was linearized, and transformed into ISS3348 by electroporation. Bacteria that had undergone homologous recombination were selected on erythromycin to obtain clones with the erythromycin resistance cassette in place of *hasA*. Erm^R^ GAS were then transformed with pEC455, a plasmid encoding kanamycin resistance and the Cre recombinase under the control of a constitutive promoter to promote excision of the Erm^R^ cassette through recombination at the *lox* sites. Kan^R^ Erm^S^ transformants were selected and passaged in THY without antibiotic to eliminate pEC455. Resulting Kan^S^ Erm^S^ clones with complete *hasA* deletion through the resolved lox72 site were verified by PCR and sequencing analyses. Sequences for all cloning and sequencing primers are given in Table S4.

*SpeB activity assay*

Cysteine protease activity in GAS culture supernatants was assessed by digestion of azocasein as previously described (2). Briefly, overnight cultures of each strain were pelleted by centrifugation (4,000 *x g*, 10 min), prior to decanting and sterile filtering of culture supernatants through 0.2 µm membranes. Supernatant aliquots were prepared by dilution in an equal volume of activation buffer (1 mM EDTA, 20 mM Dithiothreitol [DTT] in 0.1 M sodium acetate, pH 5.0) prior to incubation for 30 min at 40°C. Cysteine protease activity was assayed by the addition of 2% azocasein (Sigma) in activation buffer, followed by incubation for 40 min at 40°C. Non-digested casopeptides were precipitated using ice cold 100% trichloroacetic acid in acetone, and pelleted at 15,000 *x g* for 5 min. Cleared supernatant was measured at OD_450 nm_ to determine the quantity of remaining labeled casopeptides in solution.

*Dynamic laser light scattering*

MV samples resuspended in PBS were analyzed using a Zetasizer (Malvern). For each sample, the average of 90 measurements were taken at 20°C.

*Quantitative real-time PCR*

Analysis of differentially abundant vesicular bacterial RNA and differentially expressed ISS3348 and SF370 *covRS*-regulated genes was undertaken by quantitative real-time PCR (qPCR). For quantification of differentially abundant vesicular RNA, MVs were isolated and RNA purified as described above. For quantification of differentially expressed *covRS* genes, bacteria were grown to late-logarithmic growth phase and RNA purified as described in the main text. Extracted RNA was rigorously treated with DNA-free TURBO DNase (Ambion), and the *proS* gene was amplified by conventional PCR, to ensure complete removal of contaminating genomic DNA from all samples. cDNA libraries were prepared using the High-Capacity cDNA reverse transcription kit (Life Technologies) and were quantified using SensiFAST SYBR no-rox qPCR system (Bioline). Relative gene expression/RNA abundance was normalized to the *proS* gene, the expression of which was equally abundant between vesicular and bacterial RNA, and was previously shown to be invariant to *covRS* mutation and the growth phase (3). Details for all primers sequences are given in Table S4.

*Cellular fractionation and immunoblotting*

Cellular extracts were prepared by resuspension of GAS culture pellets in PBS containing 0.1% Triton supplemented with protease inhibitors and mechanically disrupted using a FastPrep (4 x 45 s, 4.5 m/s). Disrupted cell suspensions were pelleted (3,500 *x g*, 15 min, 4°C) and unlysed bacteria removed, prior to further centrifugation (18,000 *x g*, 30 min, 4°C) to obtain cellular extract (CE) and crude membranes (M). Membrane pellets were treated with DNase I and RNase A (10 µg/ml) for 1 h at 37°C, prior to centrifugation (65,000 *x g*, 1 h, 4°C) to obtain the membrane fraction. GAS culture supernatant proteins prior to (S1) and after (S2) isolation of MVs were precipitated by trichloroacetic acid (TCA), washed thrice with ice-cold acetone and solubilized in 1% SDS overnight. For immunoblotting, protein samples from cellular fractions (WC, C, M, S1, MV, S2) were separated by 12% SDS-PAGE and blotted onto nitrocellulose membranes. Membranes were blocked with TBS-T/5% non-fat milk, incubated with primary antisera against mouse anti-M1 1:5000 (kindly donated by Andreas Meinke, Intercell), rabbit anti-HtrA 1:1000 (kindly donated by Jeffrey Weiser), rabbit anti-RNase Y 1:5000 (Agrisera), or anonymized pooled sera from acute rheumatic fever patients (1:1000) (kindly donated by P. Nitsche-Schmitz) (4). Immunoreactive bands were visualized after washing and incubation with respective secondary antibodies (anti-mouse or anti-rabbit IgG-HRP, Amersham, anti-human IgG-HRP, Sigma or anti-human IgG-Alexa Fluor647) using ECL-substrate (Thermo Scientific) and a ChemiDoc (Biorad) or Typhoon FLA-7000 (GE Healthcare) imaging system.

*2D-DIGE*GAS MV and membrane samples were precipitated with TCA supplemented with 80 mM DTT and washed four times with acetone supplemented with 20 mM DTT. Air-dried pellets were solubilized in DIGE buffer [7 M Urea, 2 M Thiourea in 20 mM Tris, pH 8.5, 4% CHAPS] overnight at 4°C, pelleted at 13,000 x *g* for 5 min and labeled as described (5). IEF was performed using 10 cm IPG strips pH 4 - 7 (GE Healthcare). Second dimension separation was conducted on 10% SDS-PAGE gels with imaging on a Typhoon FLA-7000 reader prior to blotting onto low-fluorescent nitrocellulose or PVDF-membranes (Pall). Filters were incubated with pooled sera from acute rheumatic fever patients (1:1000) (4) overnight and immunoreactive proteins were visualized after further incubation with anti-human IgG-HRP (Sigma) antibodies (1:5000).

*Nano-LC/MS/MS analysis*

MVs and GAS membranes were prepared for proteomic analysis by delipidization and resuspension in 8 M Urea containing Protease Max (Promega), and then incubated for 30 min at room temperature. Disulfide bonds were reduced using 5 mM DTT at 56°C for 20 min, and thiol groups were alkylated using iodoacetamide at a concentration of 15 mM for 20 min at room temperature in the dark. Finally, sequencing grade trypsin (Promega) (final concentration 18 ng/µl) and 1 µl of 1% ProteaseMax^TM^ in 50 mM ammonium bicarbonate (final concentration 0.01%) were added. The digestion was performed for 3 h at 37 °C and stopped by adding trifluoroacetic acid to a final concentration of 0.5%. The supernatants were cleaned and concentrated on C_18_ material (3M Empore) on homemade C_18_ stage tips, eluted and reconstituted in 0.5% TFA. Peptides were separated on an Ultimate 3000 RSLCnano system (Dionex) using a C18 cartridge trap-column (Dionex) in backflush configuration and an in-house packed (3 µm C18 particles) analytical 50 cm x 75 µm emitter-column (New Objective). Peptides were eluted at 200 nl/min to a Q Exactive (Thermo Fisher) tandem mass spectrometer operating with a top-10 strategy and a cycle time of 1.2 s. Single 350 - 1,400 m/z MS scans at a resolution of R=70,000 were followed by higher-energy collisional dissociation fragmentation (normalized collision energy of 27) of up to 10 most intense ions (charge states +2 to +6) at R=17,500. MS and MS/MS ion target values were set to 3e6 and 5e4, respectively, and fragmented ions were dynamically excluded for 50 s.

*Mass spectrometric data analysis*

Mass-spectrometric raw data were analyzed with MaxQuant 1.4.0.8 (6). Two missed cleavages were allowed. Carbamidomethylation was set as a fixed modification, and methionine oxidation and N-terminal acetylation were set as variable modifications. First and main search MS mass tolerances were ≤20 and ≤4.5 ppm, respectively. MS/MS tolerance was ≤20 ppm. Criteria for identification were specified as following: 1 peptide, minimum length of 7 residues and false discovery rate (FDR) <1% using a target decoy approach. Match between runs was enabled, and all other parameters were default. Only proteins that were found in all three biological replicates were considered for further analysis. MaxQuant label free quantification (LFQ) algorithm normalized protein intensities were used to calculate an enrichment factor of individual proteins present in MVs and membranes. Proteins showing an enrichment factor of 2 (LFQ-intensity ratio MV/membranes equal or greater than 2) were considered for further bioinformatic analysis. Proteins exclusively identified in all three MV samples and proteins identified in all three MV samples and in membrane samples at abundances too low for LFQ (<2 quantified peptides or 3 points across a chromatographic peak) were also included in the list of MV proteins (Table S1). Prediction of protein subcellular localization was performed using PSORT3b and LocateP (7-8). Enrichment of gene ontology (GO) terms on biological process, molecular function and cellular component and involvement in KEGG signaling pathways was analyzed using DAVID (9). A False Discovery Rate of <5% was used as the cut-off for statistical significance.

*Lipid extraction and thin-layer chromatography*

Lipid extraction was conducted by modification of a previously described method (10). MV samples were diluted in MeOH, vortexed vigorously and sonicated for 1 minute prior to incubation on ice for 30 minutes. Samples were then vortexed and centrifuged (5,000 *x g* 5 min) to separate extracted lipids from the precipitated proteins. Supernatants were then transferred to glass vials and stored at -70°C before further analysis. For thin-layer chromatography (TLC), lipid extracts were dried under vacuum and dissolved in 50:50 (v/v) CHCl_3_:MeOH. Samples were then applied on ALUGRAM^®^ Nano-SIL-G plates (Macherey-Nagel) and separated using a 2-step one dimensional development system. First, the plate was developed using methyl-acetate:1-propanol:CHCl_3_:MeOH:0.25% KCl = 25 : 25 : 25 : 10 : 9 (v/v/v/v/v). Afterwards, the plate was dried and developed using hexane:diethyl ether:acetic acid = 80:20:1.5 (v/v/v). Mixtures of defined lipid standards (PE, PS, PG, CL, PI, Avanti Polar Lipids) were applied on the plate in separate lanes and co-separated to identify the individual lipid classes present in the samples. Visualization of the plates was conducted with 0.05% primuline (11), illuminated by UV-light (366 nm), photographed and densitometrically evaluated using the GelAnalyzer software (<http://www.gelanalyzer.com/>).

*Lipidomics analysis by MALDI-MS(/MS)*

Lipid profiles were measured by spotting 0.5 µL of the methanolic extracts together with an equal volume of ATT dissolved in EtOH:UHQ = 90:10 (v/v) (12) or 9AA dissolved in isopropanol:ACN = 60:40 (v/v) (13). Mass spectra were recorded using an AXIMA-CFRplus (Shimadzu) curved-field Reflectron Time-Of-Flight (RTOF) mass spectrometer equipped with a 337 nm pulsed nitrogen laser. Measurements were performed either in positive or negative mode using delayed ion extraction for unit mass resolution. The ion acceleration voltage was set to 20 kV and the reflectron analyzer was operated at 25 kV. For structural identification, a hybrid Quadrupole IonTrap (QIT)-TOF tandem-mass spectrometer (AXIMA-Resonance, Shimadzu) was used. Acquisition was performed in the low-mass range (*m*/*z* 300-1000) and high-resolution (R = 1000) ion selection modes for MS/MS experiments of monoisotopically selected precursor ions using low-energy Collision Induced Dissociation (CID) with argon as the collision gas. Each spectrum shown represents the accumulation of 300-500 single laser shots. An external calibration based on the exact mass values of the [M+H]^+^ and [M-H]^-^ ions of defined lipid standards was applied. The structural identity of the different lipid species detected in the samples was confirmed by MALDI-MS/MS spectra of authentic reference compounds. Data processing was performed using Launchpad 2.9.1 software (Shimadzu) using the Savitzky-Golay smoothing algorithm.

*Field emission scanning electron microscopy*

Bacteria were fixed with 5% formaldehyde and 2% glutaraldehyde directly in the growth medium and kept at 7°C. After centrifugation, bacteria were washed with TE buffer (20 mM Tris, 2 mM EDTA, pH 6.9) and resuspended in TE buffer. Glass cover slips of 12 mm in diameter were coated with a poly-L-lysine (Sigma) for 5 min, washed in distilled water and air-dried. 50 µl of the TE-washed fixed bacteria were placed on a cover slip and allowed to settle for 10 min. Cover slips were then fixed in 1% glutaraldehyde in TE-buffer for 10 min at room temperature and subsequently washed with TE-buffer before dehydrating in a graded series of acetone (10, 30, 50, 70, 90, 100%) on ice for 10 min for each step. Samples in the 100% acetone step were allowed to reach room temperature before another change in 100% acetone. Samples were then subjected to critical-point drying with liquid CO_2_ (CPD 030, Bal-Tec, Liechtenstein). Dried samples were covered with a gold-palladium film by sputter coating (SCD 500 Bal-Tec) prior to examination in a field emission scanning electron microscope Merlin using the Everhart-Thornley SE-detector and the Inlens SE-detector in a 50:50 ratio with an acceleration voltage of 5 kV using the SmartSEM software 5.05. Contrast and brightness were adjusted with Adobe Photoshop CS5.

*Transmission electron microscopy*

Bacteria were fixed as above, washed with cacodylate buffer (0.1 M cacodylate, 10 mM CaCl_2_, 10 mM MgCl_2_ and 0.09 M sucrose, pH 6.9) and further fixed and contrasted with 1% aqueous osmium for 1 hour at room temperature. Then, samples were dehydrated with a graded series of acetone (10%, 30%, 50%, 70%, 90%, and 100%) for 30 minutes at each step. Samples were left overnight in 2% uranyl acetate in the 70% acetone step. Samples were then infiltrated with an epoxy resin (1 part acetone/1 part resin; 1 part acetone/2 parts resin, pure resin alternating) according to the Spurr’s formula for hard resin (14). Ultrathin sections were cut with a diamond knife, picked up with butvar-coated 300 mesh grids, and counterstained with 4% aqueous uranyl acetate. Samples were examined in a TEM910 transmission electron microscope (Carl Zeiss) at an acceleration voltage of 80 kV. Images were taken at calibrated magnifications and recorded digitally with a Slow-Scan CCD-Camera (ProScan, 1024x1024,) using the ITEM-Software (Olympus Soft Imaging Solutions). Contrast and brightness were adjusted with Adobe Photoshop CS3.

**References**

1. **Mangold M**, **Siller M**, **Roppenser B**, **Vlaminckx BJM**, **Penfound TA**, **Klein R**, **Novak R**, **Novick RP**, **Charpentier E**. 2004. Synthesis of group A streptococcal virulence factors is controlled by a regulatory RNA molecule. Mol Microbiol **53**:1515–1527.

2. **Hollands A**, **Aziz RK**, **Kansal R**, **Kotb M**, **Nizet V**, **Walker MJ**. 2008. A naturally occurring mutation in *ropB* suppresses SpeB expression and reduces M1T1 group A streptococcal systemic virulence. PLoS ONE **3**:e4102.

3. **Graham MR**, **Smoot LM**, **Migliaccio CAL**, **Virtaneva K**, **Sturdevant DE**, **Porcella SF**, **Federle MJ**, **Adams GJ**, **Scott JR**, **Musser JM**. 2002. Virulence control in group A Streptococcus by a two-component gene regulatory system: Global expression profiling and in vivo infection modeling. Proc Natl Acad Sci U S A **99**:13855–13860.

4. **Dinkla K**, **Rohde M**, **Jansen WTM**, **Kaplan EL**, **Chhatwal GS**, **Talay SR**. 2003. Rheumatic fever–associated *Streptococcus pyogenes* isolates aggregate collagen. J Clin Invest **111**:1905–1912.

5. **Zellner M**, **Babeluk R**, **Diestinger M**, **Pirchegger P**, **Skeledzic S**, **Oehler R**. 2008. Fluorescence-based Western blotting for quantitation of protein biomarkers in clinical samples. ELECTROPHORESIS **29**:3621–3627.

6. **Cox J**, **Mann M**. 2008. MaxQuant enables high peptide identification rates, individualized p.p.b.-range mass accuracies and proteome-wide protein quantification. Nat Biotechnol **26**:1367–1372.

7. **Yu NY**, **Wagner JR**, **Laird MR**, **Melli G**, **Rey S**, **Lo R**, **Dao P**, **Sahinalp SC**, **Ester M**, **Foster LJ**, **Brinkman FSL**. 2010. PSORTb 3.0: improved protein subcellular localization prediction with refined localization subcategories and predictive capabilities for all prokaryotes. Bioinformatics **26**:1608–1615.

8. **Zhou M**, **Boekhorst J**, **Francke C**, **Siezen RJ**. 2008. LocateP: Genome-scale subcellular-location predictor for bacterial proteins. BMC Bioinformatics **9**:173.

9. **Huang DW**, **Sherman BT**, **Lempicki RA**. 2008. Systematic and integrative analysis of large gene lists using DAVID bioinformatics resources. Nat Protoc **4**:44–57.

10. **Zhao Z**, **Xu Y**. 2010. An extremely simple method for extraction of lysophospholipids and phospholipids from blood samples. J Lipid Res **51**:652–659.

11. **White T**, **Bursten S**, **Federighi D**, **Lewis RA**, **Nudelman E**. 1998. High-resolution separation and quantification of neutral lipid and phospholipid species in mammalian cells and sera by multi-one-dimensional thin-layer chromatography. Anal Biochem **258**:109–117.

12. **Stübiger G**, **Belgacem O**, **Rehulka P**, **Bicker W**, **Binder BR**, **Bochkov V**. 2010. Analysis of oxidized phospholipids by MALDI mass spectrometry using 6-aza-2-thiothymine together with matrix additives and disposable target surfaces. Anal Chem **82**:5502–5510.

13. **Sun G**, **Yang K**, **Zhao Z**, **Guan S**, **Han X**, **Gross RW**. 2008. Matrix-assisted laser desorption/ionization time-of-flight mass spectrometric analysis of cellular glycerophospholipids enabled by multiplexed solvent dependent analyte-matrix interactions. Anal Chem **80**:7576–7585.

14. **Spurr AR**. 1969. A low-viscosity epoxy resin embedding medium for electron microscopy. J Ultrastruct Res **26**:31–43.
