# Supplementary figures and images for: A Two-Component Regulatory System Impacts Extracellular Membrane-Derived Vesicle Production in Group A Streptococcus
Source: mBio. 2016 Nov 1;7(6):e00207-16. doi: 10.1128/mBio.00207-16 (PMC5090034; doi:10.1128/mBio.00207-16)

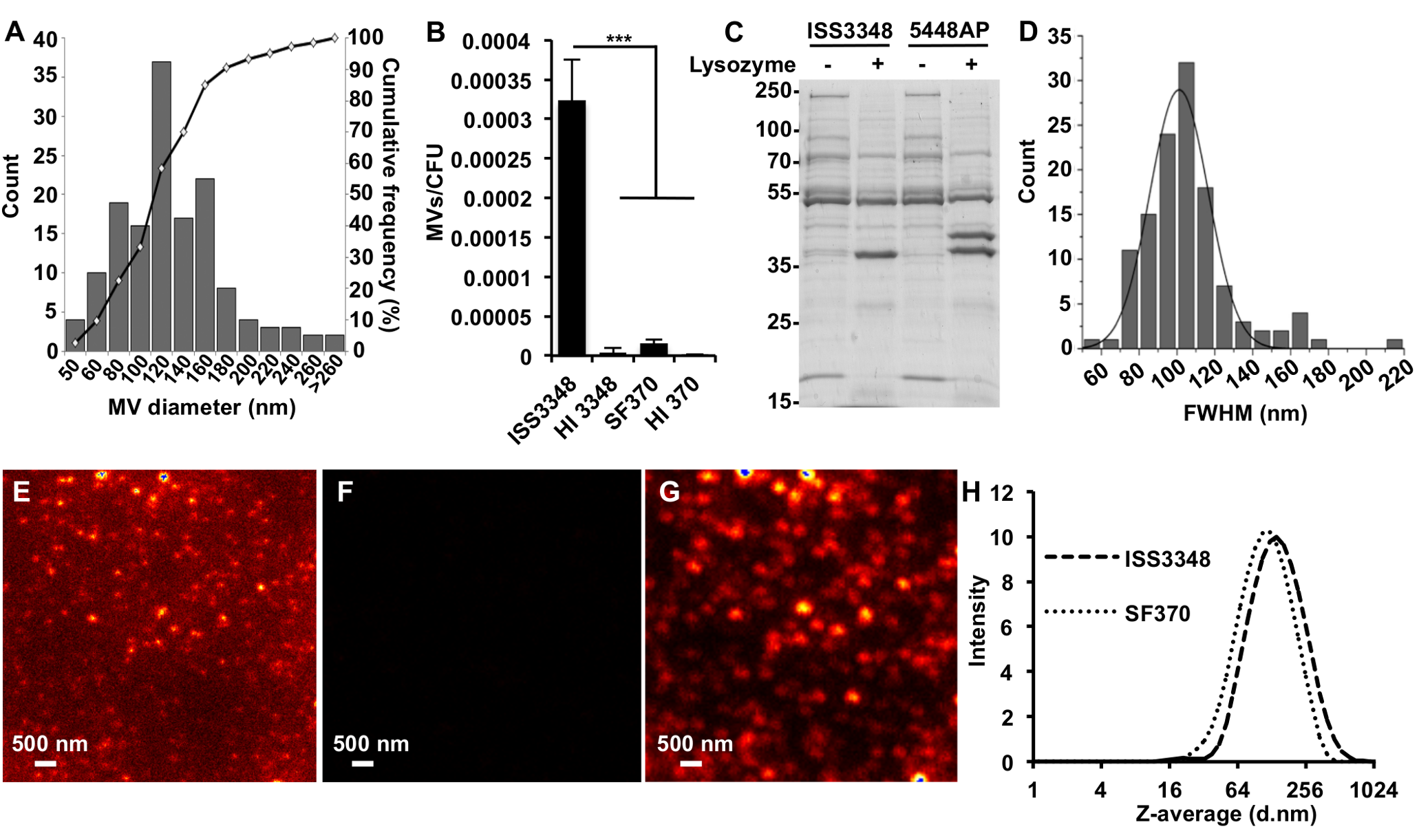

Supplement: Figure S1 — Biophysical analyses of GAS MVs. (A) Size distribution of isolated ISS3348 MVs as determined by ImageJ-based vesicle diameter measurements taken from negative-stain TEM micrographs (n = 147). Cumulative size frequency is given on the right y axis. (B) Quantification of MV production by ISS3348 and SF370 on the basis of FM1-43 dye staining pre- and post-heat inactivation (HI). MVs were harvested from late-logarithmic-growth-phase culture supernatants, purified, and quantified as described in Materials and Methods in the main text. GAS cultures were subsequently washed twice in PBS and heat inactivated at 80°C for 1 h, prior to inoculation into fresh media for 4 h, with MVs purified and quantified from HI GAS cultures as described in Materials and Methods in the main text. The results presented are pooled data ± SD from two independent experiments. Asterisks indicate statistical significance by one-way ANOVA with Tukey’s post hoc test: ***, P < 0.0001. (C) Treatment of mid-logarithmic-growth-phase ISS3348 and 5448AP cultures with or without 1 mg/ml lysozyme for 2 h prior to MV isolation. Cultures were lysozyme treated prior to MV isolation and SDS-PAGE analysis. (D) Size distribution of FM1-43-labeled ISS3348 MVs as determined by the full-width half-maximum (FWHM) profile of pixel intensities of individual anti-M1-stained MVs using stimulated emission depletion (STED) microscopy. (E and F) STED analysis of isolated ISS3348 MVs stained with mouse anti-M1 and anti-mouse Alexa Fluor 488 (E) or Alexa Fluor 488 alone (no MVs) (F). (G) Confocal microscopy analysis of isolated ISS3348 MVs stained with anti-M1 and anti-mouse Alexa Fluor 488. (H) Size distribution of ISS3348 and SF370 MVs assessed by dynamic laser light scattering analysis. Download [file mbo005163043sf1.tif]

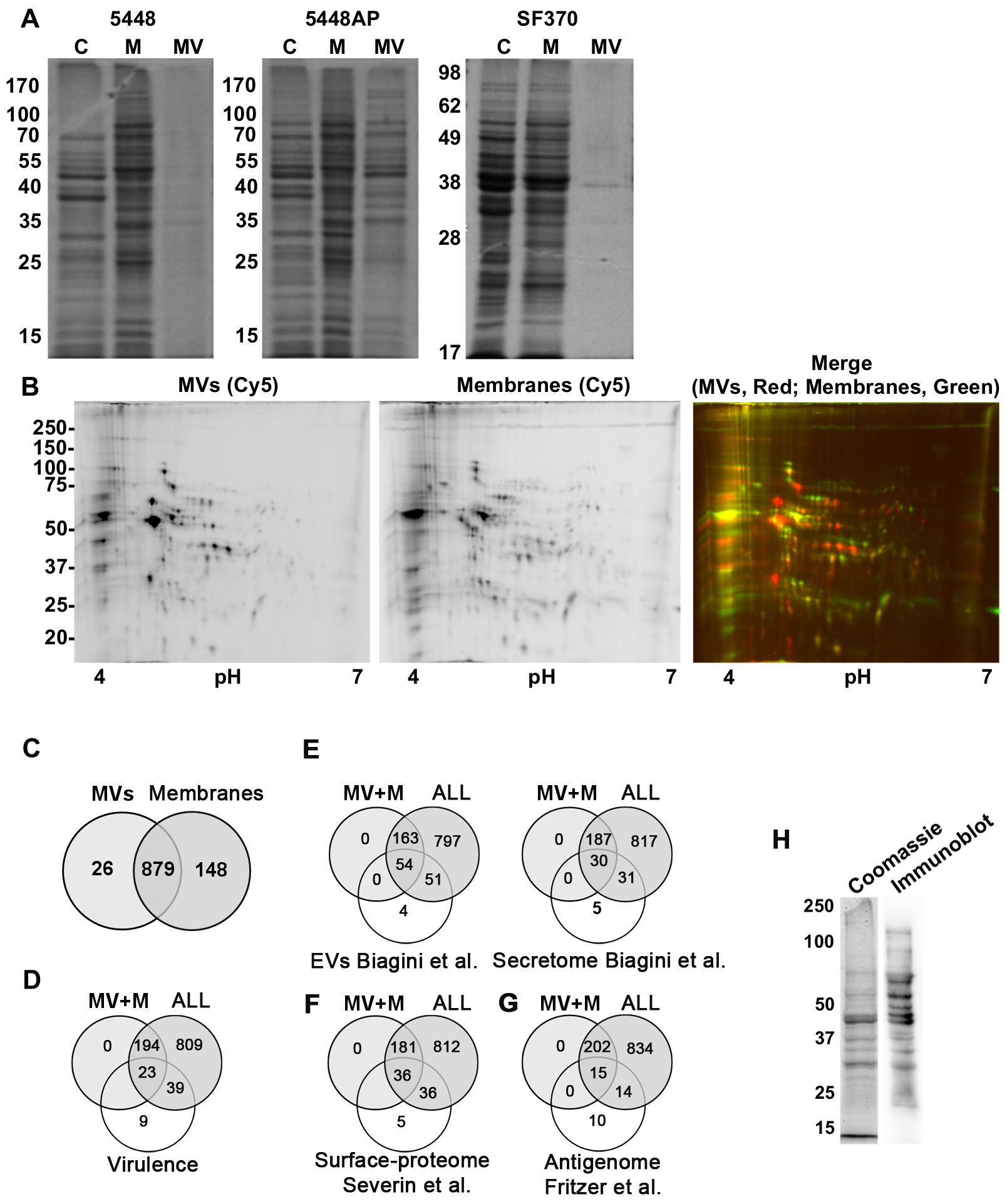

Supplement: Figure S2 — GAS MV and membrane protein profiles. (A) Protein profiles of cytoplasmic (C), membrane (M), and MV (MV) samples from strains 5448, 5448AP, and SF370. (B) Representative 2D DIGE images from Cy5-labeled ISS3348 MVs and respective Cy3-labeled membranes. Individual channels are shown in black and white, and the merged image was created in ImageJ. (C) Venn diagram showing the overlap of proteins identified in ISS3348 MVs and membranes by at least 2 peptides in each biological triplicate using nano-LC-MS/MS. (D) Venn diagram showing the overlap between virulence-associated proteins (http://www.iedb.org and http://www.mgc.ac.cn/VFs), proteins identified in ISS3348 MVs (unique and >2-fold-enriched compared to membranes [MV+M]; see Data Set S1 in the supplemental material), and all identified proteins in membrane and MV analysis (ALL [see Data Set S1]). (E) Venn diagram showing the overlap of recently published (13) secreted and ISS3348 MV proteins with proteins identified in this study. (F) Venn diagram showing the overlap of surface-associated proteins (21) with proteins identified in this study. (G) Venn diagram showing the overlap of immunogenic proteins described by Fritzer et al. (22) and proteins identified in this study. (H) Representative Coomassie-stained ISS3348 MV protein profile and immunoreactive MV protein profile after incubation with ARF patient antiserum. Download [file mbo005163043sf2.tif]

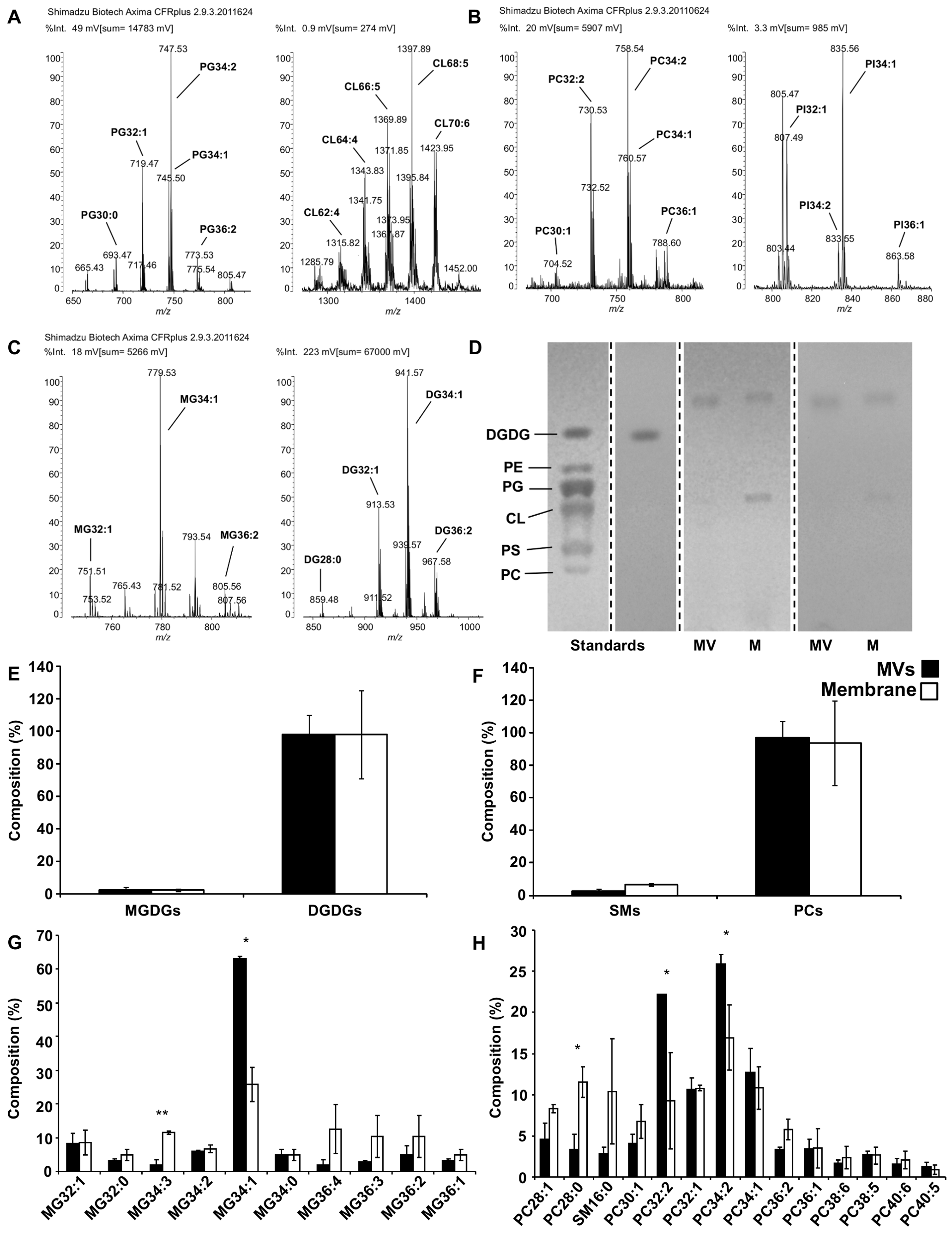

Supplement: Figure S3 — Supporting GAS MV and membrane lipidomic data. (A to C) Representative annotated ionization/mass-spectrometry spectra of different lipid classes showing mono- and diglycoglycerolipids (MG and DG, respectively [A]), anionic phospholipids (PG, CL, and PI [B]), and cationic phospholipids (PC [C]). (D) Representative TLC of lipid extracts from ISS3348 GAS MVs and corresponding bacterial membrane preparations (M). Sample loading was normalized to MV protein abundance from Coomassie staining. (E) Quantitative distribution of mono- and diglycosyldiacylglycerols (MGDG and DGDG, respectively) in GAS MVs and membranes. (F) Quantitative distribution of sphingomyelin (SM) and phosphatidylcholine (PC) in GAS MVs and membranes. (G and H) Acyl chain length and saturation level in MGDG (G) and PC (H). Download [file mbo005163043sf3.tif]

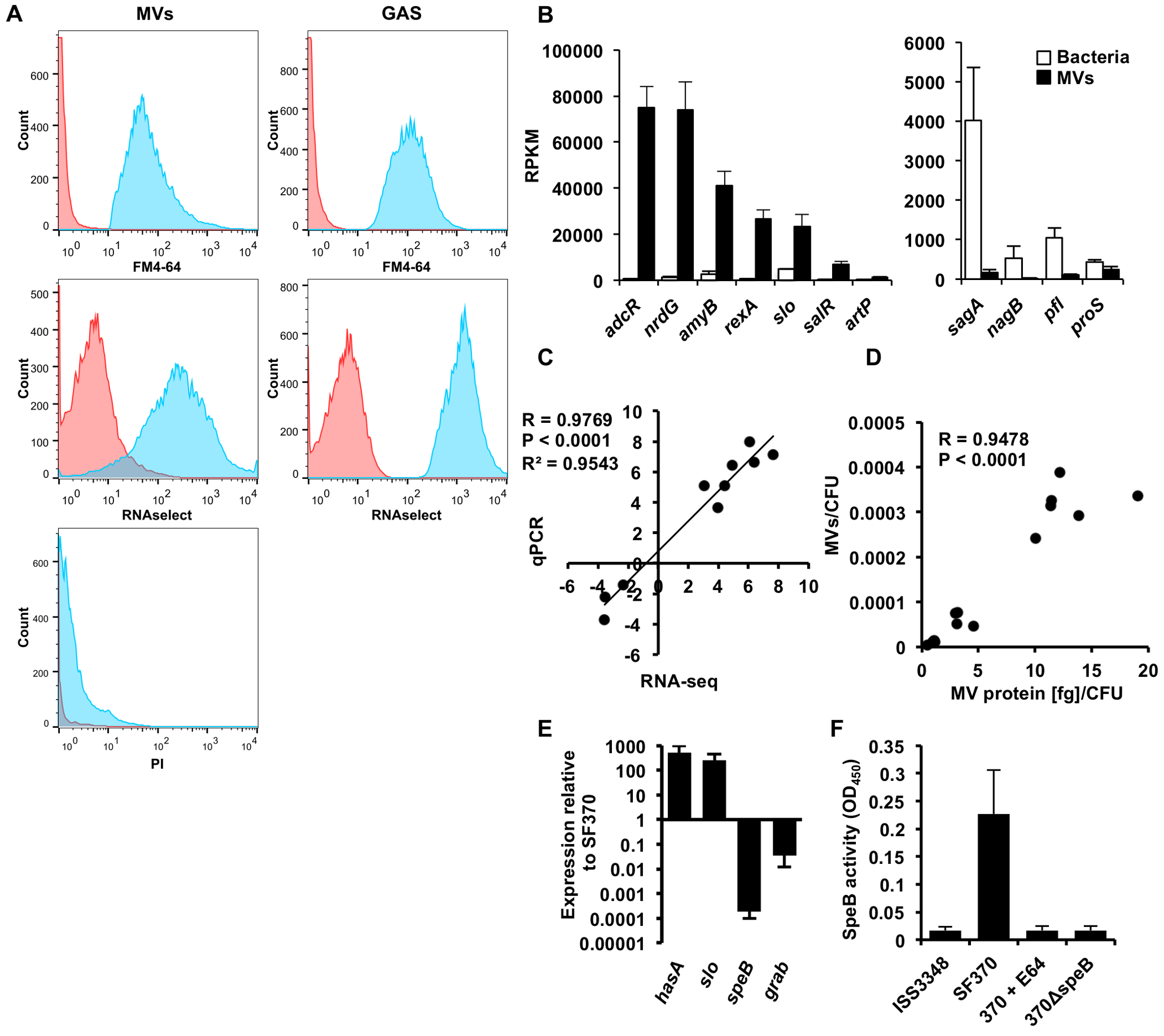

Supplement: Figure S4 — Fluorescence-activated cell sorter (FACS) and qPCR analysis of GAS MV RNA. (A) Analysis of ISS3348 MV nucleic acid content by flow cytometry. MVs were isolated as described for RNA-seq analysis and incubated with propidium iodide (PI), singly with 0.5 µg/ml of the lipophilic dye FM4-64 (Life Technologies) or with FM4-64 together with 1 µM of Syto RNAselect (Life Technologies) in the dark for 15 min, prior to being washed once in PBS and analysis of 30,000 events by flow cytometry. Negative controls consisting of PBS plus nucleic acid dyes alone had minimal fluorescence (not shown). Fluorescence of stained MVs or GAS (blue) is shown relative to that of unstained samples (top and bottom rows) or samples stained singly with FM4-64 (red) (middle row). The data shown are representative of two experiments with similar results. (B) Reads per kilobase of transcript per million mapped reads (RPKM) values for selected differentially abundant RNA species. RNA species more abundant in MVs are shown in the right panel and RNA species more abundant in bacteria in the left panel. The results presented are means ± SD. (C) Correlation of differentially abundant RNA species by RNA-seq and qPCR analyses. The Pearson’s correlation coefficient R, P value, and R2 of the linear regression line are indicated. Differences in RNA abundance determined by the DESeq2 algorithm and qPCR were log transformed and are expressed as fold change in MV RNA relative to bacterial RNA. The results presented are pooled means from independently triplicated experiments. (D) Correlation of FM1-43-based flow cytometric counting and total vesicular protein abundance quantification using Bradford protein determination. MV abundance is expressed as number of FM1-43-positive events per CFU or femtograms of MV protein per CFU. The Pearson’s correlation coefficient R and P value are indicated. (E) Expression of ISS3348 covRS-regulated genes relative to SF370 during the late logarithmic growth phase. The results pres [file mbo005163043sf4.tif]
