# Supplementary material for: A Two-Component Regulatory System Impacts Extracellular Membrane-Derived Vesicle Production in Group A Streptococcus
Source: mBio. 2016 Nov 1;7(6):e00207-16. doi: 10.1128/mBio.00207-16 (PMC5090034; doi:10.1128/mBio.00207-16)
Supplement: Table S1 — Proteins exclusively present or enriched in GAS MVs. [file mbo005163043st1.docx]

| **Table S1 Proteins exclusively present or enriched in GAS MVs** | | | | | |  |  |  |  |  |  |  |  |
| --- | --- | --- | --- | --- | --- | --- | --- | --- | --- | --- | --- | --- | --- |
| **UniProt** | **Protein name** | **Gene** | **Locus** | **∑ pep MV** | **Mean**  **Seqcov MV(%)** | | **∑ pep Mem** | **Mean**  **Seqcov Mem (%)** | **Enrichment (MV/Mem)^1^** | **SD** | **LocateP localization prediction** | | **LocateP pathway prediction** |
| Q99Z07 | Putative structural protein-phage associated | *M5005_Spy1187* | SPy_1457 | 31 | 38 | | 0 | 0 | n.a. | n.a. | Intracellular | | No pathway |
| Q99ZD8 | CAMP factor | *cfa* | SPy_1273 | 20 | 29 | | 0 | 0 | n.a. | n.a. | N-terminally anchored (No CS) | | Sec-(SPI) |
| Q99ZZ3 | Uncharacterized protein | *SPy_1002* | SPy_1002 | 15 | 47 | | 0 | 0 | n.a. | n.a. | N-terminally anchored (No CS) | | Sec-(SPI) |
| P67274 | GTP cyclohydrolase 1 type 2 homolog | SPy_0931 | SPy_0931 | 31 | 41 | | 4 | 4 | n.a. | n.a. | Intracellular | | No pathway |
| Q9A0V0 | Uncharacterized protein | M5005_Spy0502 | SPy_0604 | 24 | 64 | | 1 | 4 | n.a. | n.a. | Lipid anchored | | Sec-(SPII) |
| Q99YW1 | Uncharacterized protein | M5005_Spy1245 | SPy_1515 | 11 | 16 | | 5 | 7 | n.a. | n.a. | Intracellular | | No pathway |
| Q99XJ5 | Putative transcriptional regulator (TetR/AcrR family) | M5005_Spy1830 | SPy_2177 | 10 | 17 | | 5 | 10 | n.a. | n.a. | Intracellular | | No pathway |
| Q99ZI3 | Putative trimethylamine dehydrogenase | M5005_Spy0933 | SPy_1219 | 10 | 12 | | 5 | 5 | n.a. | n.a. | Intracellular | | No pathway |
| Q9A161 | Uncharacterized protein | M5005_Spy0364 | SPy_0446 | 10 | 47 | | 4 | 19 | n.a. | n.a. | Intracellular /TMH start AFTER 60 | | Possibly Sec- |
| Q9A1Y7 | Phosphoribosylamine--glycine ligase | purD | SPy_0032 | 9 | 9 | | 5 | 4 | n.a. | n.a. | Intracellular | | No pathway |
| Q9A0H7 | Uncharacterized protein | M5005_Spy0591 | SPy_0772 | 9 | 11 | | 4 | 5 | n.a. | n.a. | Multi-transmembrane | | Sec-(SPI) |
| Q9A1H8 | Putative ribulose-phosphate 3-epimerase | rpe | SPy_0264 | 8 | 14 | | 3 | 4 | n.a. | n.a. | Intracellular | | No pathway |
| Q9A0W1 | Uncharacterized protein | M5005_Spy0488 | SPy_0589 | 7 | 23 | | 2 | 12 | n.a. | n.a. | Multi-transmembrane | | Sec-(SPI) |
| Q9A0C9 | Putative uracil permease | pyrP | SPy_0831 | 6 | 3 | | 4 | 2 | n.a. | n.a. | Multi-transmembrane | | Sec-(SPI) |
| Q99ZC6 | Uncharacterized protein | SPy_1288 | SPy_1288 | 6 | 9 | | 3 | 4 | n.a. | n.a. | Multi-transmembrane | | Sec-(SPI) |
| Q9A1S8 | Putative glutamyl-aminopeptidase | *pepA* | SPy_0115 | 71 | 86 | | 8 | 13 | 913.59 | 328.48 | Intracellular | | No pathway |
| Q9A173 | Uncharacterized protein | *SPy_0430* | SPy_0430 | 36 | 49 | | 7 | 19 | 394.10 | 218.55 | Secretory(released) (with CS) | | Sec-(SPI) |
| P69883 | 60 kDa chaperonin | *groL* | SPy_2070 | 135 | 83 | | 83 | 64 | 88.52 | 30.80 | Intracellular | | No pathway |
| **UniProt** | **Protein name** | **Gene** | **Locus** | **∑ pep MV** | **Mean**  **Seqcov MV(%)** | | **∑ pep Mem** | **Mean**  **Seqcov Mem (%)** | **Enrichment (MV/Mem)^1^** | **SD** | **LocateP localization prediction** | | **LocateP pathway prediction** |
| Q99YL0 | Putative cysteine aminopeptidase C | *pepC* | SPy_1651 | 117 | 77 | | 42 | 38 | 83.85 | 30.24 | Intracellular | | No pathway |
| Q99XU1 | Uncharacterized protein | *M5005_Spy1729* | SPy_2033 | 21 | 40 | | 15 | 34 | 79.84 | 18.849 | Lipid anchored | | Sec-(SPII) |
| Q99YI6 | Uncharacterized protein | *SPy_1686* | SPy_1686 | 16 | 40 | | 7 | 25 | 47.39 | 26.435 | Lipid anchored | | Sec-(SPII) |
| Q99YA2 | Putative ABC transporter (Periplasmic binding protein) | *M5005_Spy1528* | SPy_1795 | 24 | 35 | | 6 | 7 | 43.77 | 27.266 | Lipid anchored | | Sec-(SPII) |
| Q9A1L1 | Glucose-6-phosphate isomerase | *pgi* | SPy_0215 | 96 | 64 | | 33 | 35 | 40.44 | 15.960 | Intracellular | | No pathway |
| Q9A1E4 | Uncharacterized protein | *M5005_Spy0271* | SPy_0319 | 53 | 65 | | 20 | 31 | 21.12 | 11.602 | Lipid anchored | | Sec-(SPII) |
| P69949 | Enolase | *eno* | SPy_0731 | 138 | 94 | | 113 | 85 | 17.81 | 3.369 | Intracellular | | No pathway |
| Q9A060 | Uncharacterized protein | *M5005_Spy0721* | SPy_0919 | 14 | 35 | | 7 | 23 | 17.68 | 1.333 | Secretory(released) (with CS) | | Sec-(SPI) |
| Q9A156 | Putative cyclophilin-type protein | *SPy_0457* | SPy_0457 | 56 | 70 | | 35 | 62 | 17.04 | 4.116 | Lipid anchored | | Sec-(SPII) |
| Q9A1E5 | Uncharacterized protein | *M5005_Spy0270* | SPy_0317 | 65 | 71 | | 47 | 62 | 16.87 | 4.423 | Lipid anchored | | Sec-(SPII) |
| Q9A1Z8 | Putative secreted protein | *sibA* | SPy_0019 | 54 | 53 | | 38 | 43 | 16.82 | 6.520 | N-terminally anchored (with CS) | | Sec-(SPI) |
| Q9A0H2 | Putative ABC transporter (Substrate-binding protein) | *M5005_Spy0596* | SPy_0778 | 33 | 41 | | 21 | 32 | 15.72 | 0.657 | Lipid anchored | | Sec-(SPII) |
| Q9A1P7 | Putative ABC transporter (Lipoprotein) | *SPy_0163* | SPy_0163 | 59 | 59 | | 49 | 57 | 11.05 | 1.831 | Lipid anchored | | Sec-(SPII) |
| Q99Y41 | Putative glutamine synthetase | *glnA* | SPy_1877 | 108 | 76 | | 82 | 71 | 10.41 | 5.373 | Intracellular | | No pathway |
| Q9A074 | Putative ABC transporter (Binding protein) | *SPy_0903* | SPy_0903 | 37 | 45 | | 17 | 21 | 9.57 | 1.350 | Lipid anchored | | Sec-(SPII) |
| Q99ZX6 | Putative dihydrolipoamide S-acetyltransferase | *acoC* | SPy_1029 | 110 | 84 | | 79 | 65 | 9.49 | 1.317 | Intracellular | | No pathway |
| Q99ZN1 | Uncharacterized protein | *M5005_Spy0878* | SPy_1157 | 23 | 64 | | 17 | 57 | 9.08 | 2.298 | N-terminally anchored (No CS) | | Sec-(SPI) |
| Q9A170 | Uncharacterized protein | *M5005_Spy0354* | SPy_0433 | 26 | 40 | | 9 | 16 | 9.07 | 5.443 | N-terminally anchored (No CS) | | Sec-(SPI) |
| Q9A0W2 | Uncharacterized protein | *M5005_Spy0487* | SPy_0588 | 44 | 84 | | 41 | 78 | 8.63 | 2.196 | Secretory(released) (with CS) | | Sec-(SPI) |
| **UniProt** | **Protein name** | **Gene** | **Locus** | **∑ pep MV** | **Mean**  **Seqcov MV(%)** | | **∑ pep Mem** | **Mean**  **Seqcov Mem (%)** | **Enrichment (MV/Mem)^1^** | **SD** | **LocateP localization prediction** | | **LocateP pathway prediction** |
| Q9A1S0 | Uncharacterized protein | *M5005_Spy0111* | SPy_0130 | 15 | 28 | | 9 | 16 | 8.45 | 4.867 | LPxTG Cell-wall anchored | | Sec-(SPI) |
| Q9A0U8 | Putative oligopeptidase | *pepF* | SPy_0606 | 67 | 42 | | 26 | 17 | 8.22 | 1.994 | Intracellular | | No pathway |
| Q99ZH4 | Putative lipoprotein | *M5005_Spy0942* | SPy_1228 | 67 | 61 | | 57 | 57 | 7.98 | 1.773 | Lipid anchored | | Sec-(SPII) |
| Q99YP8 | Putative hyaluronidase | *SPy_1600* | SPy_1600 | 26 | 17 | | 15 | 9 | 7.93 | 3.414 | Intracellular | | No pathway |
| Q99Y69 | Signal peptidase I | *spi* | SPy_1842 | 60 | 78 | | 32 | 69 | 7.62 | 2.806 | N-terminally anchored (No CS) | | Sec-(SPI) |
| Q99Y01 | Uncharacterized protein | *M5005_Spy1653* | SPy_1939 | 35 | 55 | | 30 | 51 | 7.61 | 1.934 | C-terminally anchored (with CS) | | Sec-(SPI) |
| Q99ZX5 | Putative dihydrolipoamide dehydrogenase, component E3 | *acoL* | SPy_1031 | 121 | 73 | | 109 | 67 | 7.60 | 0.631 | Intracellular | | No pathway |
| Q99YV8 | Uncharacterized protein | *M5005_Spy1248* | SPy_1519 | 35 | 55 | | 15 | 28 | 7.34 | 1.924 | Intracellular | | No pathway |
| Q9A0F1 | Uncharacterized protein | *M5005_Spy0617* | SPy_0802 | 15 | 41 | | 13 | 41 | 7.34 | 1.553 | N-terminally anchored (No CS) | | Sec-(SPI) |
| Q99XT4 | Transaldolase | *mipB* | SPy_2048 | 26 | 49 | | 9 | 14 | 7.29 | 2.120 | Intracellular | | No pathway |
| Q99Z67 | Putative NADP-dependent glyceraldehyde-3-phosphate dehydrogenase | *gapN* | SPy_1371 | 113 | 76 | | 88 | 69 | 7.16 | 1.376 | Intracellular | | No pathway |
| Q9A0S3 | Cell division protein FtsX | *ftsX* | SPy_0645 | 22 | 28 | | 10 | 15 | 6.89 | 4.390 | Multi-transmembrane | | Sec-(SPI) |
| P0A4G4 | Metal ABC transporter substrate-binding lipoprotein | *mtsA* | SPy_0453 | 80 | 76 | | 70 | 76 | 6.79 | 1.371 | Lipid anchored | | Sec-(SPII) |
| Q99ZC0 | Putative maltose/maltodextrin-binding protein | *SPy_1294* | SPy_1294 | 100 | 78 | | 84 | 72 | 6.64 | 1.888 | Lipid anchored | | Sec-(SPII) |
| Q99ZG1 | Putative phosphate ABC transporter, periplasmic phosphate-binding protein | *pstS* | SPy_1245 | 31 | 55 | | 18 | 26 | 6.56 | 2.061 | Lipid anchored | | Sec-(SPII) |
| P68840 | DNA-directed RNA polymerase subunit omega | *rpoZ* | SPy_1630 | 23 | 75 | | 15 | 59 | 6.42 | 2.220 | Intracellular | | No pathway |
| Q99YS5 | Uncharacterized protein | *M5005_Spy1288* | SPy_1564 | 87 | 85 | | 72 | 80 | 6.36 | 1.163 | Intracellular | | No pathway |
| **UniProt** | **Protein name** | **Gene** | **Locus** | **∑ pep MV** | **Mean**  **Seqcov MV(%)** | | **∑ pep Mem** | **Mean**  **Seqcov Mem (%)** | **Enrichment (MV/Mem)^1^** | **SD** | **LocateP localization prediction** | | **LocateP pathway prediction** |
| Q7DAM2 | Uncharacterized protein | *mac* | SPy_0861 | 49 | 63 | | 36 | 44 | 6.33 | 1.666 | N-terminally anchored (with CS) | | Sec-(SPI) |
| Q99Y59 | Putative transcriptional regulatory protein | *srv* | SPy_1857 | 24 | 35 | | 18 | 28 | 6.30 | 0.701 | Intracellular | | No pathway |
| Q9A1S2 | Pilin | *SPy_0128* | SPy_0128 | 58 | 57 | | 41 | 55 | 6.27 | 2.012 | LPxTG Cell-wall anchored | | Sec-(SPI) |
| P0C0G7 | Glyceraldehyde-3-phosphate dehydrogenase | *gap* | SPy_0274 | 96 | 88 | | 84 | 85 | 6.24 | 2.210 | Intracellular | | No pathway |
| Q99Y38 | Putative acid phosphatase | *lppC* | SPy_1882 | 83 | 81 | | 62 | 69 | 6.22 | 0.684 | Lipid anchored | | Sec-(SPII) |
| Q99YJ3 | Putative transketolase | *tkt* |  | 104 | 68 | | 67 | 47 | 6.17 | 0.445 |  | |  |
| Q9A081 | Putative purine nucleoside phosphorylase | *deoD2* | SPy_0894 | 37 | 70 | | 32 | 63 | 6.17 | 1.808 | Intracellular | | No pathway |
| Q99Y34 | Uncharacterized protein | *M5005_Spy1608* | SPy_1892 | 38 | 40 | | 14 | 14 | 6.06 | 2.021 | N-terminally anchored (No CS) | | Sec-(SPI) |
| Q99ZN4 | Uncharacterized protein | *M5005_Spy0875* | SPy_1154 | 37 | 53 | | 29 | 39 | 5.88 | 3.196 | N-terminally anchored (No CS) | | Sec-(SPI) |
| Q99ZK8 | Putative citrate lyase, beta subunit | *citE* | SPy_1188 | 38 | 51 | | 19 | 28 | 5.67 | 1.299 | Intracellular | | No pathway |
| Q7DAL1 | Inhibitor of complement-mediated lysis | *sic* | SPy_2016 | 32 | 44 | | 24 | 41 | 5.52 | 1.136 | N-terminally anchored (No CS) | | Sec-(SPI) |
| Q99XU7 | Immunogenic secreted protein | *isp* | SPy_2025 | 111 | 73 | | 79 | 63 | 5.45 | 1.365 | Secretory(released) (with CS) | | Sec-(SPI) |
| Q9A1S3 | Signal peptidase I | *SPy_0127* | SPy_0127 | 19 | 69 | | 12 | 40 | 5.39 | 1.441 | Intracellular | | No pathway |
| Q99Y99 | Immunogenic secreted-like protein | *isp2* | SPy_1801 | 118 | 74 | | 97 | 64 | 5.37 | 1.182 | Secretory(released) (with CS) | | Sec-(SPI) |
| Q9A1W7 | 50S ribosomal protein L16 | *rplP* | SPy_0057 | 39 | 63 | | 20 | 46 | 5.34 | 1.254 | Intracellular | | No pathway |
| Q9A0E1 | Putative folyl-polyglutamate synthetase | *folC.2* | SPy_0814 | 23 | 22 | | 13 | 15 | 5.31 | 1.221 | Intracellular | | No pathway |
| Q99ZQ0 | Xanthine phosphoribosyltransferase | *xpt* | SPy_1136 | 25 | 52 | | 21 | 47 | 4.96 | 1.402 | Intracellular | | No pathway |
| Q99YS4 | Uncharacterized protein | *SPy_1565* | SPy_1565 | 105 | 57 | | 66 | 44 | 4.88 | 0.452 | Intracellular | | No pathway |
| Q99YL1 | Putative penicillin-binding protein^1^a | *pbp1A* | SPy_1649 | 114 | 61 | | 84 | 54 | 4.41 | 0.507 | Secretory(released) (with CS) | | Sec-(SPI) |
| **UniProt** | **Protein name** | **Gene** | **Locus** | **∑ pep MV** | **Mean**  **Seqcov MV(%)** | | **∑ pep Mem** | **Mean**  **Seqcov Mem (%)** | **Enrichment (MV/Mem)^1^** | **SD** | **LocateP localization prediction** | | **LocateP pathway prediction** |
| P0C0C8 | S-ribosylhomocysteine lyase | *luxS* | SPy_1642 | 25 | 70 | | 14 | 38 | 4.35 | 1.804 | N-terminally anchored (No CS) | | Sec-(SPI) |
| Q99YF3 | Putative cell-cycle regulation histidine triad (HIT) protein | *hit* | SPy_1730 | 21 | 91 | | 19 | 87 | 4.26 | 0.497 | Intracellular | | No pathway |
| Q9A080 | Histidine protein kinase | *cpsX* | SPy_0895 | 16 | 22 | | 9 | 12 | 4.24 | 1.710 | Intracellular | | No pathway |
| P68896 | Pyrrolidone-carboxylate peptidase | *pcp* | SPy_0506 | 32 | 81 | | 26 | 73 | 4.22 | 0.989 | Intracellular | | No pathway |
| Q99Y85 | Putative aminopeptidase P XAA-pro aminopeptidase | *pepP* | SPy_1824 | 33 | 41 | | 20 | 25 | 4.16 | 0.577 | Intracellular | | No pathway |
| Q9A1S9 | Putative pyrroline carboxylate reductase | *proC* | SPy_0112 | 23 | 51 | | 14 | 30 | 4.15 | 1.162 | Intracellular | | No pathway |
| Q99ZG6 | Phosphate-specific transport system accessory protein PhoU | *phoU* | SPy_1240 | 55 | 68 | | 43 | 68 | 4.11 | 0.877 | Intracellular | | No pathway |
| Q9A046 | Putative dTDP-4-keto-6-deoxyglucose-3,5-epimerase | *cpsFP* | SPy_0935 | 30 | 48 | | 21 | 36 | 4.08 | 1.382 | Intracellular | | No pathway |
| Q99XU2 | Putative ATP-binding cassette transporter-like protein | *M5005_Spy1728* | SPy_2032 | 92 | 65 | | 64 | 56 | 4.02 | 1.195 | N-terminally anchored (No CS) | | Sec-(SPI) |
| Q9A1I9 | Putative sugar transporter sugar binding lipoprotein | *M5005_Spy0213* | SPy_0252 | 38 | 39 | | 21 | 22 | 4.02 | 2.258 | Lipid anchored | | Sec-(SPII) |
| Q9A160 | 5-methylthioadenosine/S-adenosylhomocysteine nucleosidase | *pfs* | SPy_0447 | 35 | 82 | | 28 | 62 | 3.91 | 0.631 | Intracellular | | No pathway |
| Q99XS5 | Penicillin-binding protein 2a | *pbp2A* | SPy_2059 | 100 | 37 | | 66 | 29 | 3.85 | 1.002 | Intracellular /TMH start AFTER 60 | | Possibly Sec- |
| Q99ZK7 | Putative citrate lyase, alpha subunit | *citF* | SPy_1189 | 48 | 43 | | 36 | 33 | 3.85 | 0.394 | Intracellular | | No pathway |
| Q99Z53 | Putative oligopeptidase | *pepB* | SPy_1393 | 64 | 41 | | 39 | 26 | 3.80 | 1.041 | Intracellular | | No pathway |
| P0C0I3 | Streptolysin O | *slo* | SPy_0167 | 144 | 77 | | 121 | 68 | 3.79 | 0.738 | N-terminally anchored (with CS) | | Sec-(SPI) |
| Q99YD4 | Putative trans-2-enoyl-ACP reductase II | *fabK* | SPy_1751 | 56 | 56 | | 47 | 52 | 3.76 | 0.422 | Intracellular | | No pathway |
| P63597 | Shikimate dehydrogenase (NADP(+)) | *aroE* | SPy_1584 | 23 | 36 | | 24 | 38 | 3.75 | 0.098 | Intracellular | | No pathway |
| Q99ZM4 | Putative DNA topoisomerase I | *topA* | SPy_1164 | 124 | 60 | | 108 | 58 | 3.74 | 2.194 | Intracellular | | No pathway |
| **UniProt** | **Protein name** | **Gene** | **Locus** | **∑ pep MV** | **Mean**  **Seqcov MV(%)** | | **∑ pep Mem** | **Mean**  **Seqcov Mem (%)** | **Enrichment (MV/Mem)^1^** | **SD** | **LocateP localization prediction** | | **LocateP pathway prediction** |
| Q99YD5 | Putative malonyl CoA-acyl carrier protein transacylase | *fabD* | SPy_1750 | 73 | 73 | | 61 | 72 | 3.73 | 0.356 | Intracellular | | No pathway |
| Q9A198 | Ferrichrome ABC transporter (Ferrichrome-binding protein) | *fhuD* | SPy_0385 | 61 | 59 | | 45 | 51 | 3.68 | 0.925 | Lipid anchored | | Sec-(SPII) |
| Q99ZF4 | Uncharacterized protein | *M5005_Spy0962* | SPy_1252 | 92 | 55 | | 67 | 45 | 3.65 | 1.420 | Intracellular | | No pathway |
| Q9A1P0 | Leucine--tRNA ligase | *leuS* | SPy_0173 | 113 | 51 | | 100 | 47 | 3.63 | 0.476 | Intracellular | | No pathway |
| P63770 | 10 kDa chaperonin | *groS* | SPy_2072 | 20 | 88 | | 11 | 40 | 3.58 | 1.040 | Intracellular | | No pathway |
| Q9A1C1 | Putative rRNA methylase | *M5005_Spy0299* | SPy_0356 | 32 | 54 | | 28 | 48 | 3.46 | 0.232 | Intracellular | | No pathway |
| Q99XH0 | Ribosomal RNA large subunit methyltransferase H | *rlmH* | SPy_2215 | 19 | 46 | | 11 | 33 | 3.46 | 1.204 | Intracellular | | No pathway |
| Q99YX0 | Putative deoxyribodipyrimidine photolyase | *phr* | SPy_1505 | 71 | 51 | | 63 | 45 | 3.46 | 0.651 | Intracellular | | No pathway |
| Q99YH8 | Uncharacterized protein | *M5005_Spy1390* | SPy_1697 | 30 | 36 | | 21 | 33 | 3.39 | 1.018 | N-terminally anchored (No CS) | | Sec-(SPI) |
| P65666 | Putative 2-dehydropantoate 2-reductase | *apbA* | SPy_0852 | 43 | 63 | | 45 | 62 | 3.37 | 1.374 | Intracellular | | No pathway |
| Q99YL4 | Cell cycle protein GpsB | *gpsB* | SPy_1646 | 26 | 76 | | 26 | 77 | 3.37 | 0.421 | Intracellular | | No pathway |
| Q9A096 | Phosphomevalonate kinase | *mvaK2* | SPy_0878 | 20 | 26 | | 30 | 38 | 3.24 | 0.235 | Intracellular | | No pathway |
| Q9A0H5 | Uncharacterized protein | *SPy_0775* | SPy_0775 | 18 | 32 | | 12 | 19 | 3.23 | 1.337 | Multi-transmembrane | | Sec-(SPI) |
| Q9A119 | Putative XAA-PRO dipeptidase X-PRO dipeptidase | *pepQ* | SPy_0513 | 40 | 43 | | 31 | 33 | 3.21 | 0.661 | Intracellular | | No pathway |
| P66719 | Probable DNA-directed RNA polymerase subunit delta | *rpoE* | SPy_1895 | 17 | 29 | | 9 | 23 | 3.20 | 0.292 | Intracellular | | No pathway |
| Q99YM2 | Putative acetyl-CoA:acetyltransferase | *atoB* | SPy_1637 | 35 | 37 | | 29 | 33 | 3.17 | 1.015 | Intracellular | | No pathway |
| Q7DAN3 | Possible transcriptional regulator | *M5005_Spy0104* | SPy_0122 | 66 | 77 | | 50 | 63 | 3.15 | 0.250 | Intracellular | | No pathway |
| Q9A1D7 | Ribosomal RNA small subunit methyltransferase G | *rsmG* | SPy_0329 | 40 | 60 | | 25 | 36 | 3.10 | 1.114 | Intracellular | | No pathway |
| **UniProt** | **Protein name** | **Gene** | **Locus** | **∑ pep MV** | **Mean**  **Seqcov MV(%)** | | **∑ pep Mem** | **Mean**  **Seqcov Mem (%)** | **Enrichment (MV/Mem)^1^** | **SD** | **LocateP localization prediction** | | **LocateP pathway prediction** |
| Q9A0A9 | Putative peptidoglycan hydrolase | *mur1.1* | SPy_0856 | 16 | 36 | | 7 | 14 | 3.09 | 0.036 | N-terminally anchored (with CS) | | Sec-(SPI) |
| Q9A0J7 | Uncharacterized protein | *M5005_Spy0571* | SPy_0747 | 57 | 25 | | 42 | 23 | 3.04 | 0.699 | LPxTG Cell-wall anchored | | Sec-(SPI) |
| Q99YF1 | Putative transcription regulator | *lytR* | SPy_1733 | 65 | 43 | | 47 | 39 | 3.03 | 0.707 | Secretory(released) (with CS) | | Sec-(SPI) |
| Q99ZD7 | Putative amino acid ABC transporter, periplasmic amino acid-binding protein | *M5005_Spy0982* | SPy_1274 | 71 | 70 | | 60 | 66 | 3.01 | 0.660 | Lipid anchored | | Sec-(SPII) |
| Q99YK7 | Putative amino acid permease | *aapA* | SPy_1654 | 8 | 9 | | 8 | 8 | 2.98 | 0.771 | Multi-transmembrane | | Sec-(SPI) |
| Q9A206 | Peptidyl-tRNA hydrolase | *pth* | SPy_0007 | 24 | 50 | | 27 | 52 | 2.98 | 0.195 | Intracellular | | No pathway |
| Q9A0L4 | Putative flavodoxin | *M5005_Spy0548* | SPy_0721 | 8 | 32 | | 9 | 36 | 2.98 | 0.818 | Intracellular | | No pathway |
| Q9A0A8 | Putative peptidoglycan hydrolase | *mur1.2* | SPy_0857 | 12 | 32 | | 7 | 18 | 2.97 | 0.501 | Lipid anchored | | Sec-(SPII) |
| P69887 | Triosephosphate isomerase | *tpiA* | SPy_0613 | 54 | 58 | | 51 | 58 | 2.97 | 0.738 | Intracellular | | No pathway |
| Q99XV3 | Putative laminin adhesion | *lmb* | SPy_2007 | 44 | 52 | | 25 | 37 | 2.96 | 0.559 | Lipid anchored | | Sec-(SPII) |
| P65243 | Ribose-phosphate pyrophosphokinase^1^ | *prs1* | SPy_0020 | 41 | 43 | | 38 | 43 | 2.93 | 0.464 | Intracellular | | No pathway |
| Q99YU7 | Putative peroxide resistance protein | *dpr* | SPy_1531 | 42 | 69 | | 42 | 70 | 2.86 | 0.574 | Intracellular | | No pathway |
| Q99ZT1 | Uncharacterized protein | *M5005_Spy0818* | SPy_1094 | 46 | 62 | | 42 | 61 | 2.82 | 0.789 | Lipid anchored | | Sec-(SPII) |
| Q99ZB3 | Putative cyclomaltodextrin glucanotransferase | *amyA* | SPy_1302 | 129 | 69 | | 103 | 64 | 2.78 | 0.335 | N-terminally anchored (with CS) | | Sec-(SPI) |
| Q9A1L6 | Queuine tRNA-ribosyltransferase | *tgt* | SPy_0203 | 52 | 67 | | 45 | 53 | 2.78 | 0.664 | Intracellular | | No pathway |
| P68900 | 50S ribosomal protein L10 | *rplJ* | SPy_1072 | 61 | 88 | | 56 | 83 | 2.77 | 0.539 | Intracellular | | No pathway |
| P66280 | 50S ribosomal protein L35 | *rpmI* | SPy_0805 | 12 | 33 | | 13 | 41 | 2.73 | 0.631 | Intracellular | | No pathway |
| P65850 | tRNA pseudouridine synthase A | *truA* | SPy_1901 | 14 | 22 | | 7 | 7 | 2.73 | 0.173 | Intracellular | | No pathway |
| **UniProt** | **Protein name** | **Gene** | **Locus** | **∑ pep MV** | **Mean**  **Seqcov MV(%)** | | **∑ pep Mem** | **Mean**  **Seqcov Mem (%)** | **Enrichment (MV/Mem)^1^** | **SD** | **LocateP localization prediction** | | **LocateP pathway prediction** |
| Q9A1Z9 | Putative amino acid permease | *M5005_Spy0014* | SPy_0016 | 8 | 4 | | 7 | 4 | 2.72 | 1.765 | Multi-transmembrane | | Sec-(SPI) |
| Q99YK9 | NH(3)-dependent NAD(+) synthetase | *nadE* | SPy_1652 | 46 | 76 | | 36 | 62 | 2.70 | 0.477 | Intracellular | | No pathway |
| Q99ZE7 | Uncharacterized protein | *SPy_1260* | SPy_1260 | 35 | 90 | | 41 | 95 | 2.69 | 0.450 | Intracellular | | No pathway |
| Q99ZX4 | Extracellular hyaluronate lyase | *hylA* | SPy_1032 | 115 | 57 | | 87 | 46 | 2.67 | 0.406 | Secretory(released) (with CS) | | Sec-(SPI) |
| P0C0G5 | Dihydroneopterin aldolase | *folB* | SPy_1099 | 11 | 36 | | 14 | 43 | 2.67 | 0.598 | Intracellular | | No pathway |
| P0C0E0 | DNA-directed RNA polymerase subunit beta | *rpoC* | SPy_0099 | 391 | 84 | | 345 | 80 | 2.65 | 0.318 | Intracellular | | No pathway |
| Q99Y43 | UPF0356 protein SPy_1875/M5005_Spy1594 | *SPy_1875* | SPy_1875 | 15 | 68 | | 12 | 59 | 2.65 | 0.555 | Intracellular | | No pathway |
| P0C0D0 | Ornithine carbamoyltransferase, catabolic | *arcB* | SPy_1544 | 66 | 66 | | 61 | 58 | 2.64 | 0.769 | Intracellular | | No pathway |
| Q99ZD2 | Putative signal peptidase I | *sipC* | SPy_1281 | 27 | 41 | | 20 | 29 | 2.63 | 0.852 | N-terminally anchored (No CS) | | Sec-(SPI) |
| Q99YU9 | Glucose kinase | *glcK* | SPy_1529 | 31 | 44 | | 26 | 36 | 2.62 | 0.544 | Intracellular | | No pathway |
| Q99ZQ5 | Putative phosphotransacetylase | *pta* | SPy_1128 | 67 | 79 | | 64 | 79 | 2.56 | 0.597 | Intracellular | | No pathway |
| Q99ZR1 | Putative iron-sulfur cofactor synthesis protein | *nifS2* | SPy_1122 | 20 | 28 | | 22 | 34 | 2.51 | 0.116 | Intracellular | | No pathway |
| Q99Y51 | Deoxyribose-phosphate aldolase | *deoC* | SPy_1867 | 40 | 63 | | 35 | 54 | 2.50 | 0.255 | Intracellular | | No pathway |
| Q9A0X7 | Putative signal recognition particle (Docking protein) | *ftsY* | SPy_0569 | 93 | 58 | | 81 | 55 | 2.45 | 0.735 | Intracellular | | No pathway |
| Q9A1Q0 | Putative toxic anion resistance protein | *M5005_Spy0134* | SPy_0158 | 60 | 63 | | 48 | 53 | 2.43 | 0.941 | Intracellular | | No pathway |
| Q9A1L2 | Uncharacterized protein | *SPy_0210* | SPy_0210 | 21 | 19 | | 15 | 20 | 2.40 | 0.523 | Lipid anchored | | Sec-(SPII) |
| Q99ZI9 | Formate--tetrahydrofolate ligase^1^ | *fhs1* | SPy_1213 | 62 | 46 | | 54 | 41 | 2.40 | 0.711 | Intracellular | | No pathway |
| Q9A0V2 | Putative endolysin, phage associated | *M5005_Spy0500* | SPy_0601 | 16 | 25 | | 11 | 17 | 2.39 | 0.589 | N-terminally anchored (with CS) | | Sec-(SPI) |
| **UniProt** | **Protein name** | **Gene** | **Locus** | **∑ pep MV** | **Mean**  **Seqcov MV(%)** | | **∑ pep Mem** | **Mean**  **Seqcov Mem (%)** | **Enrichment (MV/Mem)^1^** | **SD** | **LocateP localization prediction** | | **LocateP pathway prediction** |
| Q99YL9 | Putative oxidoreductase | *M5005_Spy1347* | SPy_1640 | 36 | 47 | | 31 | 51 | 2.35 | 0.316 | Intracellular | | No pathway |
| Q99YK4 | Putative amino acid ABC transproter (Permease protein) | *M5005_Spy1363* | SPy_1658 | 6 | 5 | | 6 | 5 | 2.35 | 1.364 | Multi-transmembrane | | Sec-(SPI) |
| Q99XV9 | Surface lipoprotein | *dppA* | SPy_2000 | 139 | 74 | | 128 | 75 | 2.34 | 0.545 | Lipid anchored | | Sec-(SPII) |
| Q99ZR0 | Ribose-phosphate pyrophosphokinase 2 | *prs2* | SPy_1123 | 48 | 60 | | 42 | 55 | 2.33 | 0.506 | Intracellular | | No pathway |
| Q9A1B3 | Tyrosine recombinase XerD-like | *SPy_0365* | SPy_0365 | 20 | 31 | | 10 | 15 | 2.33 | 0.827 | Intracellular | | No pathway |
| Q99XR7 | Putative alkyl hydroperoxidase | *ahpC* | SPy_2079 | 33 | 46 | | 27 | 48 | 2.33 | 0.730 | Intracellular | | No pathway |
| Q9A0G8 | Putative RNA polymerase sigma 42 protein | *rpoD* | SPy_0782 | 63 | 66 | | 51 | 58 | 2.30 | 0.537 | Intracellular | | No pathway |
| Q9A0C5 | Uncharacterized protein | *SPy_0836* | SPy_0836 | 58 | 70 | | 48 | 56 | 2.30 | 0.662 | N-terminally anchored (No CS) | | Sec-(SPI) |
| Q99Z84 | Putative shikimate kinase | *aroK* | SPy_1351 | 13 | 28 | | 14 | 38 | 2.29 | 0.513 | Intracellular | | No pathway |
| Q9A1G0 | Penicillin-binding protein (D-alanyl-D-alanine carboxypeptidase) | *dacA* | SPy_0292 | 31 | 31 | | 23 | 25 | 2.29 | 0.418 | N-terminally anchored (No CS) | | Sec-(SPI) |
| Q99XH2 | ABC transporter, ATP-binding protein | *M5005_Spy1861* | SPy_2210 | 103 | 63 | | 90 | 61 | 2.29 | 0.638 | Intracellular | | No pathway |
| Q9A1U1 | DNA-directed RNA polymerase subunit beta | *rpoB* | SPy_0098 | 269 | 69 | | 252 | 68 | 2.28 | 0.285 | Intracellular | | No pathway |
| Q99ZD1 | Putative pyruvate kinase | *pyk* | SPy_1282 | 153 | 83 | | 146 | 87 | 2.27 | 0.461 | Intracellular | | No pathway |
| Q9A1F1 | Uncharacterized protein | *M5005_Spy0264* | SPy_0309 | 26 | 51 | | 18 | 35 | 2.26 | 0.707 | Intracellular | | No pathway |
| Q99ZQ2 | Putative ABC transporter (Binding protein) |  |  | 13 | 9 | | 8 | 5 | 2.24 | 0.668 |  | |  |
| Q9A1B7 | Glutamate racemase | *murI* | SPy_0361 | 37 | 48 | | 36 | 51 | 2.24 | 0.078 | Intracellular | | No pathway |
| Q99YS1 | Valine--tRNA ligase | *valS* | SPy_1568 | 105 | 51 | | 110 | 53 | 2.24 | 0.696 | Intracellular | | No pathway |
| Q9A0Z6 | Uncharacterized protein | *M5005_Spy0447* | SPy_0540 | 17 | 26 | | 15 | 20 | 2.23 | 0.491 | Intracellular | | No pathway |
| **UniProt** | **Protein name** | **Gene** | **Locus** | **∑ pep MV** | **Mean**  **Seqcov MV(%)** | | **∑ pep Mem** | **Mean**  **Seqcov Mem (%)** | **Enrichment (MV/Mem)^1^** | **SD** | **LocateP localization prediction** | | **LocateP pathway prediction** |
| Q9A0F4 | Peptidase T | *pepT* | SPy_0799 | 40 | 47 | | 39 | 48 | 2.21 | 0.911 | Intracellular | | No pathway |
| Q99YC5 | Putative D,D-carboxypeptidase | *M5005_Spy1502* | SPy_1765 | 25 | 41 | | 22 | 47 | 2.21 | 0.487 | N-terminally anchored (No CS) | | Sec-(SPI) |
| Q9A1H5 | Putative CMP-binding factor | *cbf* | SPy_0267 | 48 | 58 | | 41 | 53 | 2.20 | 0.598 | Intracellular | | No pathway |
| P66751 | Holliday junction ATP-dependent DNA helicase RuvA | *ruvA* | SPy_2119 | 13 | 25 | | 15 | 23 | 2.19 | 1.218 | Intracellular | | No pathway |
| P67372 | DegV domain-containing protein SPy_1493/M5005_Spy1226 | *SPy_1493* | SPy_1493 | 42 | 63 | | 47 | 77 | 2.18 | 0.171 | Intracellular | | No pathway |
| P66710 | DNA-directed RNA polymerase subunit alpha | *rpoA* | SPy_0080 | 125 | 89 | | 101 | 77 | 2.17 | 0.225 | Intracellular | | No pathway |
| Q9A1C3 | Membrane protein insertase YidC 2 | *yidC2* | SPy_0351 | 24 | 17 | | 26 | 17 | 2.17 | 0.464 | Multi-transmembrane(Lipid modified N-termini) | | Sec-(SPII) |
| Q99ZL4 | Putative transcriptional regulator | *SPy_1179* | SPy_1179 | 18 | 23 | | 19 | 27 | 2.16 | 0.260 | Intracellular | | No pathway |
| P65631 | Membrane protein insertase YidC^1^ | *yidC1* | SPy_0247 | 10 | 9 | | 13 | 9 | 2.15 | 0.627 | Multi-transmembrane(Lipid modified N-termini) | | Sec-(SPII) |
| P67295 | UPF0154 protein SPy_0359/M5005_Spy0302 | *SPy_0359* | SPy_0359 | 12 | 32 | | 12 | 35 | 2.14 | 0.683 | N-terminally anchored (No CS) | | Sec-(SPI) |
| P60811 | Foldase protein PrsA^1^ | *prsA1* | SPy_1390 | 92 | 67 | | 87 | 67 | 2.13 | 0.454 | Lipid anchored | | Sec-(SPII) |
| Q99YN6 | Uncharacterized protein | *M5005_Spy1328* | SPy_1617 | 33 | 51 | | 16 | 35 | 2.13 | 0.388 | Intracellular | | No pathway |
| Q99ZC4 | Uncharacterized protein | *M5005_Spy1054* | SPy_1290 | 12 | 29 | | 7 | 13 | 2.13 | 0.711 | Lipid anchored | | Sec-(SPII) |
| P65259 | L-lactate dehydrogenase | *ldh* | SPy_1151 | 73 | 52 | | 71 | 62 | 2.08 | 0.745 | Intracellular | | No pathway |
| Q9A116 | Putative glucosyl transferase | *M5005_Spy0426* | SPy_0516 | 69 | 53 | | 62 | 58 | 2.06 | 0.196 | Intracellular | | No pathway |
| Q99XS2 | Uncharacterized protein | *SPy_2065* | SPy_2065 | 77 | 49 | | 74 | 45 | 2.06 | 0.467 | N-terminally anchored (No CS) | | Sec-(SPI) |
| Q99YA0 | Uncharacterized protein | *M5005_Spy1530* | SPy_1798 | 133 | 41 | | 118 | 38 | 2.02 | 0.243 | C-terminally anchored (with CS) | | Sec-(SPI) |
| **UniProt** | **Protein name** | **Gene** | **Locus** | **∑ pep MV** | **Mean**  **Seqcov MV(%)** | | **∑ pep Mem** | **Mean**  **Seqcov Mem (%)** | **Enrichment (MV/Mem)^1^** | **SD** | **LocateP localization prediction** | | **LocateP pathway prediction** |
| Q99Y25 | Putative type I site-specific deoxyribonuclease | *hsdS* | SPy_1905 | 30 | 28 | | 9 | 7 | 2.02 | 0.000 | Intracellular | | No pathway |
| Q99ZY6 | Uncharacterized protein | *M5005_Spy0743* | SPy_1016 | 14 | 21 | | 9 | 12 | 2.01 | 0.083 | N-terminally anchored (No CS) | | Sec-(SPI) |
| Q9A168 | Uncharacterized protein | *M5005_Spy0357* | SPy_0437 | 31 | 58 | | 34 | 65 | 2.01 | 0.172 | N-terminally anchored (No CS) | | Sec-(SPI) |
| P69884 | ATP-dependent Clp protease proteolytic subunit | *clpP* | SPy_0395 | 20 | 36 | | 20 | 38 | 2.00 | 0.514 | Intracellular | | No pathway |
| **^1^**: relative enrichment (LFQ intensities MV/Mem) | | | | | | | | | | | |  |  |
