# Supplementary material for: A Two-Component Regulatory System Impacts Extracellular Membrane-Derived Vesicle Production in Group A Streptococcus
Source: mBio. 2016 Nov 1;7(6):e00207-16. doi: 10.1128/mBio.00207-16 (PMC5090034; doi:10.1128/mBio.00207-16)
Supplement: Table S2 — Detailed MALDI-MS lipidomic analysis of ISS3348 MVs and membranes. [file mbo005163043st2.docx]

**Table S2 Detailed MADLI-MS lipidomic analysis of ISS3348 MVs and membranes**

| **A) Glycoglycerolipids (MG- and DGDG)** | | |  |  | **Composition (%)^5^** | | | |
| --- | --- | --- | --- | --- | --- | --- | --- | --- |
| **Ion type [M+Na]+^1^** | **Lipid species (CN:DB)^2^** | **Fatty acid residues^3^** | **[M+Na-162]+ / [M+Na-RCOOH-162]+^4^** | **[M+Na-RCOOH]+^4^** | **MV** | SD  (n = 3) | **Membrane** | SD  (n = 3) |
| m/z 751.54 | **MG32:1** | 16:0/16:1 |  |  | 8.239 | 3.080 | 8.497 | 3.697 |
| m/z 753.55 | **MG32:0** | 16:0/16:0 | ― | ― | 3.338 | 0.435 | 4.793 | 1.541 |
| m/z 775.53 | **MG34:3** | 16:1/18:2 | ― | ― | 1.875 | 1.634 | 11.438 | 0.462 |
| m/z 777.57 | **MG34:2** | 16:1/18:1 | ― | ― | 5.895 | 0.234 | 6.645 | 1.078 |
| m/z 779.58 | **MG34:1** | 16:0/18:1 | ― | ― | 63.068 | 0.804 | 25.817 | 5.084 |
| m/z 781.58 | **MG34:0** | 16:0/18:0 | ― | ― | 4.853 | 1.708 | 4.793 | 1.541 |
| m/z 801.55 | **MG36:4** | 18:2/18:2 | ― | ― | 1.875 | 1.634 | 12.527 | 7.241 |
| m/z 803.57 | **MG36:3** | 18:1/18:2 | ― | ― | 2.817 | 0.301 | 10.349 | 6.316 |
| m/z 805.56 | **MG36:2** | 18:1/18:1 | ― | ― | 4.901 | 2.645 | 10.349 | 6.316 |
| m/z 807.59 | **MG36:1** | 18:0/18:1 | ― | ― | 3.338 | 0.435 | 4.793 | 1.541 |
| m/z 829.4 | **DG26:1** | 12:0/14:1 | ― | ― | 0.101 | 0.059 | 0.082 | 0.074 |
| m/z 831.5 | **DG26:0** | 12:0/14:0 | ― | ― | 0.655 | 0.429 | 0.107 | 0.039 |
| m/z 857.50 | **DG28:1** | 14:0/14:1 | ― | ― | 0.343 | 0.236 | 0.107 | 0.039 |
| m/z 859.51 | **DG28:0** | 14:0/14:0 | ― | ― | 1.692 | 0.778 | 0.320 | 0.116 |
| m/z 885.55 | **DG30:1** | 14:1/16:0 14:0/16:1 12:0/18:1 | 723/497/495 | 685/659/657/631/629/603 | 0.548 | 0.320 | 0.146 | 0.017 |
| m/z 887.56 | **DG30:0** | 14:0/16:0 | ― | ― | 1.034 | 0.469 | 0.412 | 0.203 |
| m/z 911.57 | **DG32:2** | 16:1/16:1 | 749/495 | 657 | 1.443 | 0.178 | 0.744 | 0.294 |
| m/z 913.58 | **DG32:1** | 16:1/16:0 | 751/497/495 | 657/659 | 14.099 | 0.341 | 12.650 | 1.067 |
| m/z 915.59 | **DG32:0** | 16:0/16:0 | ― | ― | 9.121 | 0.069 | 7.640 | 2.653 |
| m/z 939.58 | **DG34:2** | 16:1/18:1 | 777/523/495 | 685/657 | 8.523 | 0.051 | 7.697 | 0.679 |
| m/z 941.62 | **DG34:1** | 16:0/18:1 | 779/523/497 | 685/659 | 37.560 | 4.411 | 47.126 | 1.600 |
| m/z 943.60 | **DG34:0** | 16:0/18:0 | ― | ― | 9.866 | 0.119 | 9.578 | 1.034 |
| m/z 963.6 | **DG36:4** | 18:2/18:2 | ― | ― | 0.274 | 0.246 | 0.439 | 0.052 |
| m/z 965.62 | **DG36:3** | 18:1/18:2 | 803/523/521 | 685/683 | 0.378 | 0.244 | 0.253 | 0.021 |
| m/z 967.62 | **DG36:2** | 18:1/18:1 | 805/523 | 685 | 6.289 | 0.617 | 6.206 | 0.702 |
| m/z 969.63 | **DG36:1** | 18:1/18:0 | ― | ― | 5.691 | 0.598 | 5.255 | 0.418 |
| m/z 971.61 | **DG36:0** | 18:0/18:0 | ― | ― | 0.891 | 0.267 | 0.399 | 0.004 |
| m/z 979.5 | **DG37:3** | 19:1 (19:0cy)/18:2 | ― | ― | 0.076 | 0.066 | 0.008 | 0.004 |
| m/z 981.5 | **DG37:2** | 19:1 (19:0cy)/18:1 | ― | ― | 0.155 | 0.012 | 0.107 | 0.039 |
| m/z 983.6 | **DG37:1** | 19:1 (19:0cy)/18:0 | ― | ― | 0.271 | 0.135 | 0.023 | 0.011 |
| m/z 985.6 | **DG37:0** | 19:0/18:0 | ― | ― | 0.281 | 0.150 | 0.082 | 0.074 |
| m/z 1001.6 | **DG39:6** | 19:3/20:3 | ― | ― | 0.101 | 0.059 | 0.050 | 0.042 |
| m/z 1003.5 | **DG39:5** | 19:2/20:3 | ― | ― | 0.095 | 0.010 | 0.107 | 0.039 |
| m/z 1007.5 | **DG39:4** | 19:1(19:0cy)/20:3 | ― | ― | 0.050 | 0.013 | 0.107 | 0.039 |
| m/z 1009.5 | **DG39:3** | 19:1(19:0cy)/20:2 | ― | ― | 0.223 | 0.057 | 0.107 | 0.039 |
| m/z 1031.5 | **DG41:5** | 21:1(21:0cy)/20:4 | ― | ― | 0.159 | 0.023 | 0.107 | 0.039 |
| m/z 1035.5 | **DG41:3** | 21:1(21:0cy)/20:2 | ― | ― | 0.064 | 0.033 | 0.107 | 0.039 |
| m/z 1037.6 | **DG41:2** | 21:1(21:0cy)/20:1 | ― | ― | 0.025 | 0.006 | 0.107 | 0.039 |
| **B) Anionic Phospholipids** | | |  |  | **Composition (%)^5^** | | | |
| **Ion type [M+Na]+^1^** | **Lipid species (CN:DB)^2^** | **Fatty acid residues^3^** | **Carboxylate anions [RCOO]-^4^** | **[M-H-74-RCOOH]-^4^** | **MV** | SD  (n = 3) | **Membrane** | SD  (n = 3) |
| m/z 637.37 | **PG26:0** | 12:0/14:0 | 199/227 | 353/381 | 4.089 | 2.412 | 4.426 | 3.607 |
| m/z 665.41 | **PG28:0** | 12:0/16:0 | 199/255 | 391/409 | 3.219 | 0.082 | 17.427 | 1.665 |
| m/z 693.45 | **PG30:0** | 14:0/16:0 | 227/255 | 363/391 | 4.666 | 0.142 | 2.100 | 0.319 |
| m/z 717.50 | **PG32:2** | 16:1/16:1 | ― | ― | 5.613 | 0.488 | 6.838 | 3.589 |
| m/z 719.52 | **PG32:1** | 16:1/16:0 | 253/255 | 389/391 | 16.434 | 0.350 | 14.164 | 0.298 |
| m/z 721.51 | **PG32:0** | 16:0/16:0 | ― | ― | 4.880 | 0.300 | 7.790 | 4.893 |
| m/z 743.49 | **PG34:3** | 16:1/18:2 | ― | ― | 2.081 | 0.708 | 8.314 | 4.687 |
| m/z 745.52 | **PG34:2** | 16:1/18:1 | 253/281 | 389/417 | 16.442 | 0.903 | 14.390 | 2.672 |
| m/z 747.52 | **PG34:1** | 16:0/18:1 | 255/281 | 391/417 | 31.005 | 0.391 | 18.052 | 0.781 |
| m/z 773.56 | **PG36:2** | 18:1/18:1 | 281 | 417 | 7.406 | 0.081 | 7.638 | 4.224 |
| m/z 775.57 | **PG36:1** | 18:1/18:0 | 281/283 | 417/419 | 4.166 | 0.428 | 2.725 | 0.565 |
| m/z 1315.87 | **CL62:4** | 12:0/14:0/18:2/18:2 | ― | ― | 3.638 | 1.682 | 2.977 | 1.336 |
| m/z 1317.89 | **CL62:3** | 12:0/14:0/18:2/18:1 | ― | ― | 4.203 | 0.883 | 4.444 | 0.739 |
| m/z 1319.86 | **CL62:2** | 12:0/14:0/18:1/18:1 | ― | ― | 3.357 | 0.846 | 5.050 | 3.950 |
| m/z 1339.87 | **CL64:6** | 10:0/18:2/18:2/18:2 | ― | ― | 4.704 | 2.125 | 2.864 | 1.496 |
| m/z 1341.89 | **CL64:5** | 12:0/16:1/18:2/18:2 | ― | ― | 5.206 | 3.367 | 1.996 | 0.050 |
| m/z 1343.86 | **CL64:4** | 12:0/16:0/18:2/18:2 16:1/16:1/16:1/16:1 | ― | ― | 4.141 | 0.004 | 3.654 | 0.379 |
| m/z 1345.91 | **CL64:3** | 16:1/16:1/16:1/16:0 14:0/16:1/16:1/18:1 | ― | ― | 3.106 | 1.467 | 4.895 | 1.377 |
| m/z 1347.92 | **CL64:2** | 16:1/16:1/16:0/16:0 14:0/16:1/16:0/18:1 | ― | ― | 3.357 | 0.846 | 6.553 | 0.948 |
| m/z 1369.92 | **CL66:5** | 16:1/16:1/16:1/18:2 | ― | ― | 6.713 | 1.692 | 4.296 | 2.244 |
| m/z 1371.92 | **CL66:4** | 16:1/16:0/16:1/18:2 | ― | ― | 7.025 | 2.744 | 4.782 | 1.217 |
| m/z 1373.95 | **CL66:3** | 16:1/16:0/16:1/18:1 | ― | ― | 4.423 | 0.403 | 10.397 | 1.934 |
| m/z 1375.98 | **CL66:2** | 16:1/16:0/16:1/18:0 | ― | ― | 4.109 | 1.017 | 5.608 | 5.158 |
| m/z 1395.93 | **CL68:6** | 16:1/16:1/18:2/18:2 | ― | ― | 5.521 | 0.981 | 2.786 | 1.167 |
| m/z 1397.95 | **CL68:5** | 16:1/16:1/18:1/18:2 16:1/16:0/18:2/18:2 | ― | ― | 9.631 | 2.892 | 5.311 | 0.807 |
| m/z 1399.97 | **CL68:4** | 16:1/16:1/18:0/18:2 16:0/16:0/18:2/18:2 | ― | ― | 6.744 | 0.216 | 4.931 | 4.200 |
| m/z 1401.98 | **CL68:3** | 16:1/16:1/18:0/18:1 16:0/16:0/18:1/18:2 | ― | ― | 2.102 | 1.022 | 5.573 | 2.335 |
| m/z 1404.00 | **CL68:2** | 16:1/16:1/18:0/18:0 16:0/16:0/18:0/18:2 | ― | ― | 3.263 | 0.713 | 10.249 | 4.917 |
| m/z 1423.97 | **CL70:6** | 16:1/18:2/18:2/18:1 | ― | ― | 6.367 | 0.748 | 3.125 | 1.646 |
| m/z 1425.98 | **CL70:5** | 16:0/18:2/18:2/18:1 | ― | ― | 5.302 | 2.622 | 3.880 | 0.059 |
| m/z 1427.98 | **CL70:4** | 16:0/18:2/18:2/18:0 | ― | ― | 3.545 | 1.112 | 1.883 | 0.110 |
| m/z 1430.01 | **CL70:3** | 16:0/18:2/18:1/18:0 | ― | ― | 3.544 | 1.816 | 4.747 | 1.606 |
| **C) Cationic Phospholipids** | | |  |  | **Composition (%)^5^** | | | |
| **Ion type [M+Na]+^1^** | **Lipid species (CN:DB)^2^** | **Fatty acid residues^3^** |  |  | **MV** | SD  (n = 3) | **Membrane** | SD  (n = 3) |
| m/z 676.45 | **PC28:1** | 12:0/16:1 | ― | ― | 4.588 | 1.967 | 8.322 | 0.499 |
| m/z 678.45 | **PC28:0** | 12:0/16:0 | ― | ― | 3.281 | 1.907 | 11.553 | 1.839 |
| m/z 703.54 | **SM16:0** | d18:1/16:0 | ― | ― | 2.847 | 0.747 | 10.385 | 6.399 |
| m/z 704.52 | **PC30:1** | 14:0/16:1 | ― | ― | 4.062 | 1.074 | 6.755 | 2.081 |
| m/z 730.50 | **PC32:2** | 16:1/16:1 | ― | ― | 22.158 | 0.030 | 9.258 | 5.867 |
| m/z 732.52 | **PC32:1** | 16:1/16:0 | ― | ― | 10.619 | 1.380 | 10.790 | 0.354 |
| m/z 758.54 | **PC34:2** | 16:1/18:1 | ― | ― | 25.888 | 1.154 | 16.913 | 3.964 |
| m/z 760.54 | **PC34:1** | 16:0/18:1 | ― | ― | 12.683 | 2.935 | 10.808 | 2.560 |
| m/z 786.56 | **PC36:2** | 18:1/18:1 | ― | ― | 3.300 | 0.303 | 5.787 | 1.269 |
| m/z 788.57 | **PC36:1** | 18:1/18:0 | ― | ― | 3.410 | 1.140 | 3.502 | 2.408 |
| m/z 806.51 | **PC38:6** | 16:0/22:6 18:1/20:5 18:2/20:4 | ― | ― | 1.649 | 0.395 | 2.381 | 1.359 |
| m/z 808.51 | **PC38:5** | 18:1/20:4 | ― | ― | 2.699 | 0.407 | 2.617 | 1.025 |
| m/z 834.51 | **PC40:6** | 18:0/22:6 20:1/20:5 | ― | ― | 1.557 | 0.662 | 2.045 | 1.107 |
| m/z 836.54 | **PC40:5** | 20:1/20:4 | ― | ― | 1.259 | 0.538 | 0.879 | 0.574 |
|  | |  |  |  |  | | | |
| **D) Fatty acids** | |  |  |  | **Composition (%)^5^** | | | |
| **Ion type [M+Na]+^1^** | **Lipid species (CN:DB)^2^** |  |  |  | **MV** | SD  (n = 3) | **Membrane** | SD  (n = 3) |
| m/z 253.2 | **C16:1** | ― | ― | ― | 18.385 | 2.712 | 19.902 | 0.367 |
| m/z 255.2 | **C16:0** | ― | ― | ― | 29.642 | 4.656 | 25.254 | 0.781 |
| m/z 279.2 | **C18:2** | ― | ― | ― | 3.249 | 1.208 | 5.027 | 1.217 |
| m/z 281.2 | **C18:1** | ― | ― | ― | 45.334 | 3.634 | 43.990 | 1.765 |
| m/z 283.3 | **C18:0** | ― | ― | ― | 4.390 | 2.955 | 5.827 | 0.599 |
|  |  |  |  |  |  |  |  |  |
| **^1^:** Type of ions the different lipid species of the four lipid classes (A-D) are detected using the MALDI matrix systems in positive and negative mode (m/z values of the detected peaks are shown). | | | | | | | | |
| **^2^:** Short hand designation of the individual lipid species based on the ratio of total carbon atom (CN) to double bond (DB) number of their fatty acid residues. | | | | | | | | |
| **^3^:** Fatty acid residues of the individual lipid species identified based on the detection of characteristic fragment ions from MALDI-MS/MS spectra and on search of the LIPID MAPS database (http://www.lipidmaps.org/). | | | | | | | | |
| **^4^:** Characteristic fragment ions detected from selected precursor ions of the different lipid classes. Note that only the most abundant lipid species were subjected to MS/MS analysis. | | | | | | | | |
| **^5^:** Values display the relative composition of the individual lipid species within a specific lipid class based on the signal intensities of the MALDI mass spectra (sum of signal intensities = 100%). Shown is the mean of triplicate measurements (n = 3) of individual samples including standard deviation (SD) of extracellular membrane vesicles (MV) and the membrane of the ISS3348 strain of *S. pyogenes*. | | | | | | | | |
