# Supplementary material for: A Two-Component Regulatory System Impacts Extracellular Membrane-Derived Vesicle Production in Group A Streptococcus
Source: mBio. 2016 Nov 1;7(6):e00207-16. doi: 10.1128/mBio.00207-16 (PMC5090034; doi:10.1128/mBio.00207-16)
Supplement: Table S3 — DESeq2 analysis of GAS MV and bacterial cellular RNAs. [file mbo005163043st3.docx]

| **Table S3 DESeq2 analysis of GAS MV and bacterial cellular RNAs** | | | | | |
| --- | --- | --- | --- | --- | --- |
| *Gene or locus* | *Normalized count base mean* | *log2 fold change* | *Standard error* | *Wald test statistic* | *Benjamini-Hochberg adjusted P value* |
| *M5005_Spy_0073* | 50169.199 | 7.791 | 0.301 | 25.915 | 3.85E-145 |
| *adcR* | 36473.240 | 7.647 | 0.259 | 29.575 | 5.36E-189 |
| *nrdG* | 50735.994 | 6.366 | 0.301 | 21.169 | 7.85E-97 |
| *rexA* | 108599.580 | 6.063 | 0.316 | 19.160 | 2.28E-79 |
| *hsdR* | 28155.593 | 5.929 | 0.324 | 18.314 | 1.38E-72 |
| *M5005_Spy_1087* | 624.838 | 5.622 | 0.310 | 18.122 | 3.68E-71 |
| *lacA.1* | 8509.897 | 5.590 | 0.301 | 18.550 | 2.02E-74 |
| *M5005_Spy_1626* | 5543.193 | 5.423 | 0.338 | 16.046 | 7.47E-56 |
| *M5005_Spy_0281* | 56639.771 | 5.126 | 0.281 | 18.214 | 7.59E-72 |
| *rpmD* | 4705.940 | 5.064 | 0.232 | 21.818 | 9.05E-103 |
| *M5005_Spy_1650* | 12218.240 | 5.028 | 0.281 | 17.912 | 1.49E-69 |
| *salR* | 4685.756 | 4.928 | 0.320 | 15.380 | 2.39E-51 |
| *M5005_Spy_0836* | 6683.119 | 4.888 | 0.415 | 11.785 | 2.17E-30 |
| *M5005_Spy_0547* | 3025.172 | 4.685 | 0.241 | 19.408 | 2.29E-81 |
| *M5005_Spy_0652* | 1894.409 | 4.562 | 0.283 | 16.129 | 2.11E-56 |
| *amyB* | 79543.088 | 4.434 | 0.451 | 9.833 | 1.81E-21 |
| *M5005_Spy_0007* | 2475.531 | 4.323 | 0.298 | 14.503 | 9.04E-46 |
| *M5005_Spy_1261* | 4787.878 | 4.117 | 0.313 | 13.166 | 1.02E-37 |
| *M5005_Spy_0112* | 358.260 | 4.041 | 0.274 | 14.724 | 3.91E-47 |
| *M5005_Spy_0074* | 394.866 | 3.982 | 0.308 | 12.915 | 2.66E-36 |
| *artP* | 1229.937 | 3.954 | 0.328 | 12.070 | 7.73E-32 |
| *M5005_Spy_0447* | 1219.017 | 3.896 | 0.365 | 10.687 | 3.54E-25 |
| *msrA* | 101.150 | 3.831 | 0.354 | 10.819 | 9.13E-26 |
| *M5005_Spy_0243* | 3249.638 | 3.823 | 0.249 | 15.359 | 3.12E-51 |
| *M5005_Spy_0364* | 1384.248 | 3.582 | 0.285 | 12.550 | 2.64E-34 |
| *sagA* | 398.383 | -3.571 | 0.315 | -11.347 | 2.93E-28 |
| *adcC* | 1631.866 | 3.563 | 0.293 | 12.151 | 3.05E-32 |
| *nagB* | 224.125 | -3.534 | 0.403 | -8.762 | 2.93E-17 |
| *rpmJ* | 1021.094 | 3.516 | 0.228 | 15.430 | 1.18E-51 |
| *recF* | 494.688 | 3.509 | 0.239 | 14.706 | 4.84E-47 |
| *M5005_Spy_0075* | 298.345 | 3.406 | 0.319 | 10.690 | 3.47E-25 |
| *M5005_Spy_0165* | 20.116 | 3.402 | 0.545 | 6.240 | 3.21E-09 |
| *M5005_Spy_1138* | 260.890 | -3.399 | 0.372 | -9.133 | 1.18E-18 |
| *M5005_Spy_0467* | 55.492 | 3.394 | 0.376 | 9.030 | 2.87E-18 |
| *opuABC* | 1032.629 | 3.322 | 0.371 | 8.961 | 5.16E-18 |
| *recX* | 786.873 | 3.295 | 0.273 | 12.049 | 9.60E-32 |
| *rpsE* | 4799.500 | 3.278 | 0.300 | 10.945 | 2.38E-26 |
| *M5005_Spy_0500* | 2063.144 | 3.255 | 0.317 | 10.274 | 2.41E-23 |
| *M5005_Spy_0276* | 1205.174 | 3.226 | 0.288 | 11.193 | 1.62E-27 |
| *M5005_Spy_1152* | 66.239 | 3.219 | 0.354 | 9.097 | 1.58E-18 |
| *priA* | 1242.070 | 3.212 | 0.253 | 12.699 | 4.11E-35 |
| *covR* | 5876.727 | 3.140 | 0.252 | 12.474 | 6.35E-34 |
| *Gene or locus* | *Normalized count base mean* | *log2 fold change* | *Standard error* | *Wald test statistic* | *Benjamini-Hochberg adjusted P value* |
| *M5005_Spy_1624* | 128.903 | 3.085 | 0.321 | 9.596 | 1.71E-20 |
| *M5005_Spy_0978* | 226.365 | 3.080 | 0.290 | 10.613 | 7.72E-25 |
| *slo* | 49444.069 | 3.048 | 0.309 | 9.857 | 1.48E-21 |
| *pepN* | 5035.962 | 2.995 | 0.277 | 10.813 | 9.55E-26 |
| *rpmC* | 1292.769 | 2.975 | 0.256 | 11.622 | 1.44E-29 |
| *M5005_Spy_0670* | 74.245 | 2.969 | 0.464 | 6.398 | 1.25E-09 |
| *uppS* | 1523.400 | 2.942 | 0.240 | 12.281 | 6.32E-33 |
| *divIC* | 601.240 | 2.939 | 0.238 | 12.367 | 2.33E-33 |
| *M5005_Spy_1153* | 49.008 | 2.895 | 0.489 | 5.919 | 2.13E-08 |
| *M5005_Spy_0448* | 24.078 | 2.888 | 0.472 | 6.125 | 6.47E-09 |
| *M5005_Spy_0574* | 3867.981 | 2.864 | 0.295 | 9.724 | 5.12E-21 |
| *hrcA* | 4806.662 | -2.754 | 0.321 | -8.581 | 1.31E-16 |
| *recD* | 833.447 | 2.754 | 0.239 | 11.541 | 3.42E-29 |
| *uppP* | 356.116 | 2.722 | 0.221 | 12.322 | 3.96E-33 |
| *M5005_Spy_1149* | 569.624 | 2.721 | 0.238 | 11.419 | 1.32E-28 |
| *M5005_Spy_1756* | 1619.022 | 2.714 | 0.296 | 9.164 | 8.99E-19 |
| *lacB.2* | 1564.431 | 2.711 | 0.366 | 7.408 | 1.36E-12 |
| *ppc* | 3344.061 | 2.686 | 0.287 | 9.372 | 1.41E-19 |
| *rpsQ* | 2444.962 | 2.666 | 0.332 | 8.031 | 1.23E-14 |
| *M5005_Spy_0835* | 155.343 | -2.653 | 0.417 | -6.370 | 1.48E-09 |
| *M5005_Spy_1285* | 429.189 | 2.619 | 0.339 | 7.724 | 1.29E-13 |
| *M5005_Spy_0651* | 3412.456 | 2.567 | 0.245 | 10.499 | 2.42E-24 |
| *purF* | 465.255 | 2.534 | 0.326 | 7.771 | 9.02E-14 |
| *M5005_Spy_0009* | 206.452 | 2.501 | 0.399 | 6.269 | 2.72E-09 |
| *cdd* | 3418.915 | 2.491 | 0.278 | 8.962 | 5.16E-18 |
| *M5005_Spy_0280* | 4018.609 | 2.491 | 0.288 | 8.652 | 7.21E-17 |
| *M5005_Spy_1696* | 774.773 | 2.431 | 0.212 | 11.452 | 9.21E-29 |
| *M5005_Spy_0336* | 279.579 | 2.405 | 0.249 | 9.665 | 8.84E-21 |
| *M5005_Spy_0361* | 435.372 | 2.394 | 0.241 | 9.921 | 8.05E-22 |
| *dpr* | 2151.095 | -2.392 | 0.251 | -9.524 | 3.35E-20 |
| *rpsB* | 547.838 | -2.363 | 0.213 | -11.073 | 5.98E-27 |
| *M5005_Spy_1786* | 11.721 | 2.362 | 0.542 | 4.361 | 5.11E-05 |
| *M5005_Spy_0377* | 37.544 | -2.361 | 0.403 | -5.860 | 3.05E-08 |
| *purM* | 176.911 | 2.358 | 0.321 | 7.338 | 2.23E-12 |
| *estA* | 330.308 | 2.350 | 0.257 | 9.129 | 1.21E-18 |
| *sodA* | 555.161 | -2.341 | 0.235 | -9.950 | 6.08E-22 |
| *M5005_Spy_0384* | 1934.628 | 2.337 | 0.232 | 10.084 | 1.62E-22 |
| *pfl* | 1643.706 | -2.330 | 0.143 | -16.298 | 1.45E-57 |
| *flaR* | 66.876 | -2.324 | 0.312 | -7.454 | 9.77E-13 |
| *M5005_Spy_1689* | 627.204 | -2.294 | 0.249 | -9.232 | 4.84E-19 |
| *M5005_Spy_0118* | 13.718 | -2.273 | 0.508 | -4.476 | 3.17E-05 |
| *pfs* | 2445.023 | 2.271 | 0.256 | 8.884 | 1.01E-17 |
| *M5005_Spy_0076* | 305.726 | 2.260 | 0.215 | 10.508 | 2.23E-24 |
| *Gene or locus* | *Normalized count base mean* | *log2 fold change* | *Standard error* | *Wald test statistic* | *Benjamini-Hochberg adjusted P value* |
| *M5005_Spy_1640* | 74.353 | -2.257 | 0.306 | -7.366 | 1.84E-12 |
| *rpsT* | 190.373 | -2.234 | 0.212 | -10.557 | 1.37E-24 |
| *M5005_Spy_0610* | 608.482 | 2.229 | 0.283 | 7.882 | 3.85E-14 |
| *pflC* | 613.771 | -2.217 | 0.205 | -10.793 | 1.17E-25 |
| *rbfA* | 230.662 | 2.208 | 0.329 | 6.719 | 1.60E-10 |
| *M5005_Spy_0793* | 2649.665 | 2.191 | 0.225 | 9.730 | 4.91E-21 |
| *M5005_Spy_1556* | 127.737 | -2.186 | 0.246 | -8.879 | 1.04E-17 |
| *dnaN* | 1622.437 | 2.185 | 0.255 | 8.559 | 1.58E-16 |
| *M5005_Spy_0533* | 151.689 | -2.182 | 0.197 | -11.046 | 7.86E-27 |
| *M5005_Spy_1137* | 156.972 | -2.179 | 0.323 | -6.745 | 1.34E-10 |
| *M5005_Spy_1382* | 439.988 | -2.157 | 0.266 | -8.117 | 6.17E-15 |
| *xerS* | 332.972 | 2.142 | 0.189 | 11.316 | 4.08E-28 |
| *clpE* | 2747.400 | -2.125 | 0.249 | -8.518 | 2.21E-16 |
| *lacA.2* | 153.050 | -2.115 | 0.433 | -4.888 | 4.90E-06 |
| *infC* | 463.736 | -2.096 | 0.173 | -12.121 | 4.26E-32 |
| *ulaD* | 21.241 | 2.096 | 0.590 | 3.554 | NA |
| *M5005_Spy_1830* | 22.350 | -2.061 | 0.419 | -4.924 | 4.16E-06 |
| *M5005_Spy_1535* | 6.835 | 2.052 | 0.634 | 3.237 | 0.003128744 |
| *M5005_Spy_0861* | 20.624 | -2.052 | 0.406 | -5.053 | 2.19E-06 |
| *rplK* | 809.127 | -2.034 | 0.137 | -14.854 | 6.26E-48 |
| *M5005_Spy_1260* | 2.411 | 2.020 | 0.708 | 2.854 | NA |
| *M5005_Spy_1148* | 17.085 | 2.019 | 0.497 | 4.059 | 0.000176277 |
| *rplD* | 271.989 | -2.012 | 0.231 | -8.713 | 4.29E-17 |
| *rplQ* | 199.157 | -1.997 | 0.261 | -7.665 | 2.04E-13 |
| *M5005_Spy_1068* | 5.427 | 1.990 | 0.657 | 3.027 | 0.006008115 |
| *rpsR* | 203.285 | 1.988 | 0.222 | 8.952 | 5.58E-18 |
| *M5005_Spy_1514* | 604.510 | -1.978 | 0.241 | -8.206 | 3.01E-15 |
| *M5005_Spy_1141* | 396.685 | 1.963 | 0.224 | 8.754 | 3.10E-17 |
| *M5005_Spy_0446* | 442.256 | 1.963 | 0.267 | 7.352 | 2.04E-12 |
| *M5005_Spy_0444* | 103.237 | 1.953 | 0.305 | 6.394 | 1.27E-09 |
| *M5005_Spy_1864* | 61.335 | -1.945 | 0.409 | -4.757 | 9.01E-06 |
| *M5005_Spy_1089* | 52.505 | -1.944 | 0.502 | -3.876 | 0.000348036 |
| *rplW* | 159.136 | -1.937 | 0.279 | -6.956 | 3.29E-11 |
| *M5005_Spy_0111* | 4100.419 | 1.930 | 0.254 | 7.593 | 3.43E-13 |
| *regR* | 105.529 | -1.929 | 0.252 | -7.645 | 2.35E-13 |
| *vicK* | 2191.234 | 1.928 | 0.221 | 8.714 | 4.28E-17 |
| *manO* | 64.156 | -1.914 | 0.305 | -6.276 | 2.60E-09 |
| *M5005_Spy_1700* | 19.597 | -1.913 | 0.465 | -4.110 | 0.0001427 |
| *copY* | 318.914 | -1.911 | 0.386 | -4.952 | 3.63E-06 |
| *M5005_Spy_0399* | 123.376 | 1.908 | 0.275 | 6.942 | 3.62E-11 |
| *rpmB* | 123.342 | -1.901 | 0.330 | -5.760 | 5.33E-08 |
| *M5005_Spy_0452* | 11.241 | 1.896 | 0.627 | 3.023 | 0.006069196 |
| *M5005_Spy_0115* | 599.273 | -1.894 | 0.203 | -9.340 | 1.85E-19 |
| *Gene or locus* | *Normalized count base mean* | *log2 fold change* | *Standard error* | *Wald test statistic* | *Benjamini-Hochberg adjusted P value* |
| *mraY* | 2895.605 | 1.850 | 0.275 | 6.716 | 1.61E-10 |
| *M5005_Spy_0186* | 322.204 | -1.845 | 0.275 | -6.718 | 1.60E-10 |
| *M5005_Spy_0596* | 2329.452 | 1.841 | 0.208 | 8.837 | 1.51E-17 |
| *M5005_Spy_0152* | 18.697 | 1.830 | 0.661 | 2.770 | NA |
| *phoU* | 390.250 | 1.814 | 0.288 | 6.306 | 2.18E-09 |
| *dppE* | 431.900 | 1.804 | 0.293 | 6.162 | 5.17E-09 |
| *M5005_Spy_1340* | 1300.634 | 1.803 | 0.285 | 6.335 | 1.84E-09 |
| *M5005_Spy_0714* | 261.998 | -1.803 | 0.246 | -7.321 | 2.51E-12 |
| *udp* | 569.918 | -1.794 | 0.346 | -5.190 | 1.11E-06 |
| *secA* | 9459.694 | 1.794 | 0.261 | 6.871 | 5.81E-11 |
| *M5005_Spy_0488* | 23.270 | -1.793 | 0.390 | -4.596 | 1.88E-05 |
| *M5005_Spy_0458* | 67.405 | -1.782 | 0.318 | -5.600 | 1.29E-07 |
| *rpsJ* | 287.425 | -1.780 | 0.191 | -9.339 | 1.85E-19 |
| *rpmH* | 69.708 | -1.753 | 0.262 | -6.696 | 1.84E-10 |
| *M5005_Spy_0143* | 7.362 | -1.752 | 0.576 | -3.042 | 0.005739265 |
| *M5005_Spy_0456* | 110.821 | -1.746 | 0.327 | -5.338 | 5.26E-07 |
| *M5005_Spy_1239* | 78.788 | -1.742 | 0.300 | -5.808 | 4.09E-08 |
| *rpsS* | 343.981 | -1.742 | 0.177 | -9.835 | 1.81E-21 |
| *M5005_Spy_0937* | 360.667 | 1.739 | 0.182 | 9.559 | 2.39E-20 |
| *lacF* | 258.525 | 1.729 | 0.286 | 6.048 | 1.00E-08 |
| *M5005_Spy_0316* | 359.592 | 1.728 | 0.238 | 7.274 | 3.49E-12 |
| *malE* | 176.617 | -1.728 | 0.250 | -6.922 | 4.15E-11 |
| *lacB.1* | 491.809 | 1.726 | 0.236 | 7.305 | 2.81E-12 |
| *pta* | 146.740 | -1.726 | 0.317 | -5.445 | 3.01E-07 |
| *sclA* | 63.830 | -1.725 | 0.311 | -5.553 | 1.68E-07 |
| *cpsFP* | 961.493 | 1.724 | 0.279 | 6.186 | 4.46E-09 |
| *rpmF* | 1717.540 | -1.723 | 0.197 | -8.748 | 3.26E-17 |
| *M5005_Spy_0772* | 88.586 | 1.721 | 0.334 | 5.149 | 1.37E-06 |
| *rpsF* | 336.424 | -1.720 | 0.220 | -7.822 | 6.02E-14 |
| *czcD* | 45.650 | -1.718 | 0.410 | -4.189 | 0.000104463 |
| *M5005_Spy_0534* | 602.851 | -1.717 | 0.150 | -11.474 | 7.31E-29 |
| *tsf* | 268.346 | -1.716 | 0.163 | -10.540 | 1.61E-24 |
| *M5005_Spy_1223* | 931.893 | -1.709 | 0.170 | -10.036 | 2.60E-22 |
| *carA* | 1457.417 | 1.703 | 0.302 | 5.638 | 1.06E-07 |
| *M5005_Spy_1082* | 17.128 | 1.701 | 0.470 | 3.618 | 0.000880827 |
| *M5005_Spy_0846* | 26.728 | -1.692 | 0.356 | -4.752 | 9.15E-06 |
| *M5005_Spy_1829* | 13.193 | -1.687 | 0.589 | -2.866 | 0.009520826 |
| *ssb3* | 697.137 | -1.681 | 0.164 | -10.262 | 2.69E-23 |
| *M5005_Spy_1856* | 630.683 | -1.675 | 0.274 | -6.110 | 7.05E-09 |
| *recG* | 762.180 | 1.672 | 0.270 | 6.189 | 4.41E-09 |
| *tpiA* | 723.866 | -1.667 | 0.172 | -9.689 | 7.01E-21 |
| *M5005_Spy_0676* | 16.760 | -1.658 | 0.553 | -3.000 | 0.006496485 |
| *M5005_Spy_0611* | 56.795 | 1.652 | 0.284 | 5.814 | 3.95E-08 |
| *Gene or locus* | *Normalized count base mean* | *log2 fold change* | *Standard error* | *Wald test statistic* | *Benjamini-Hochberg adjusted P value* |
| *gyrB* | 3361.873 | 1.651 | 0.243 | 6.804 | 9.02E-11 |
| *asp* | 328.107 | -1.649 | 0.209 | -7.884 | 3.81E-14 |
| *msrB* | 109.915 | -1.644 | 0.239 | -6.882 | 5.44E-11 |
| *sibA* | 1151.019 | 1.641 | 0.301 | 5.444 | 3.02E-07 |
| *M5005_Spy_0716* | 58.906 | -1.639 | 0.323 | -5.076 | 1.97E-06 |
| *tig* | 2888.161 | -1.633 | 0.181 | -9.019 | 3.12E-18 |
| *rplC* | 460.361 | -1.622 | 0.175 | -9.254 | 4.01E-19 |
| *tuf* | 5427.846 | -1.620 | 0.188 | -8.634 | 8.38E-17 |
| *M5005_Spy_0003* | 50.679 | -1.619 | 0.337 | -4.805 | 7.35E-06 |
| *proA* | 519.749 | 1.612 | 0.264 | 6.105 | 7.20E-09 |
| *ppaC* | 847.135 | -1.608 | 0.172 | -9.370 | 1.42E-19 |
| *M5005_Spy_0486* | 238.950 | -1.608 | 0.227 | -7.083 | 1.38E-11 |
| *M5005_Spy_0154* | 1.568 | 1.598 | 0.700 | 2.284 | NA |
| *tkt* | 445.029 | -1.598 | 0.176 | -9.063 | 2.13E-18 |
| *M5005_Spy_0202* | 286.935 | -1.598 | 0.213 | -7.512 | 6.40E-13 |
| *gatC* | 231.403 | 1.592 | 0.280 | 5.693 | 7.77E-08 |
| *clpL* | 1780.935 | -1.582 | 0.170 | -9.289 | 2.90E-19 |
| *M5005_Spy_1083* | 117.868 | 1.578 | 0.370 | 4.270 | 7.50E-05 |
| *M5005_Spy_0498* | 30.709 | -1.577 | 0.455 | -3.467 | 0.001493191 |
| *era* | 1073.112 | 1.576 | 0.229 | 6.880 | 5.47E-11 |
| *M5005_Spy_1703* | 3.984 | 1.576 | 0.672 | 2.347 | 0.037321582 |
| *cutC* | 65.007 | -1.573 | 0.302 | -5.214 | 9.89E-07 |
| *acoA* | 823.433 | -1.571 | 0.245 | -6.420 | 1.09E-09 |
| *trx* | 424.594 | -1.566 | 0.172 | -9.106 | 1.47E-18 |
| *rpsD* | 148.843 | -1.566 | 0.199 | -7.870 | 4.21E-14 |
| *M5005_Spy_0451* | 4.528 | 1.565 | 0.692 | 2.262 | 0.045582451 |
| *M5005_Spy_0930* | 498.813 | 1.563 | 0.308 | 5.074 | 1.99E-06 |
| *M5005_Spy_1144* | 8.874 | -1.561 | 0.541 | -2.885 | 0.009068999 |
| *M5005_Spy_1628* | 28.432 | 1.557 | 0.429 | 3.631 | 0.000842016 |
| *M5005_Spy_0468* | 16.690 | 1.557 | 0.447 | 3.484 | 0.001404554 |
| *amrA* | 1781.589 | 1.554 | 0.270 | 5.748 | 5.71E-08 |
| *M5005_Spy_1592* | 148.182 | -1.552 | 0.259 | -5.998 | 1.35E-08 |
| *M5005_Spy_0780* | 2019.621 | 1.544 | 0.260 | 5.946 | 1.84E-08 |
| *M5005_Spy_0785* | 194.144 | 1.533 | 0.208 | 7.370 | 1.80E-12 |
| *M5005_Spy_0678* | 29.288 | -1.532 | 0.397 | -3.855 | 0.000374233 |
| *M5005_Spy_1785* | 11.952 | -1.527 | 0.532 | -2.869 | 0.00945598 |
| *pfkA* | 2351.747 | -1.520 | 0.243 | -6.264 | 2.78E-09 |
| *M5005_Spy_0099* | 65.118 | -1.519 | 0.392 | -3.879 | 0.000346043 |
| *dltD* | 4724.696 | 1.513 | 0.191 | 7.926 | 2.79E-14 |
| *M5005_Spy_0038* | 1703.515 | 1.506 | 0.249 | 6.043 | 1.03E-08 |
| *M5005_Spy_1081* | 11.407 | 1.506 | 0.517 | 2.916 | 0.008277946 |
| *M5005_Spy_0784* | 311.995 | 1.500 | 0.190 | 7.889 | 3.70E-14 |
| *M5005_Spy_1134* | 136.406 | -1.494 | 0.209 | -7.142 | 9.13E-12 |
| *Gene or locus* | *Normalized count base mean* | *log2 fold change* | *Standard error* | *Wald test statistic* | *Benjamini-Hochberg adjusted P value* |
| *M5005_Spy_0900* | 38.536 | 1.492 | 0.336 | 4.446 | 3.59E-05 |
| *M5005_Spy_1828* | 141.997 | 1.490 | 0.459 | 3.247 | 0.003040836 |
| *secG* | 129.783 | -1.489 | 0.213 | -6.980 | 2.78E-11 |
| *ptsK* | 299.926 | -1.488 | 0.249 | -5.985 | 1.46E-08 |
| *ulaA* | 83.046 | 1.481 | 0.700 | 2.117 | NA |
| *secY* | 8252.373 | 1.480 | 0.203 | 7.295 | 3.01E-12 |
| *M5005_Spy_0457* | 121.843 | -1.479 | 0.340 | -4.349 | 5.40E-05 |
| *M5005_Spy_1471* | 115.944 | -1.472 | 0.254 | -5.786 | 4.64E-08 |
| *M5005_Spy_0733* | 510.702 | 1.469 | 0.210 | 6.990 | 2.61E-11 |
| *tyrS* | 232.661 | -1.467 | 0.223 | -6.568 | 4.17E-10 |
| *malX* | 2900.810 | -1.460 | 0.381 | -3.830 | 0.000410105 |
| *pepC* | 10197.092 | 1.453 | 0.239 | 6.085 | 8.11E-09 |
| *M5005_Spy_0271* | 494.802 | 1.448 | 0.257 | 5.637 | 1.06E-07 |
| *emm1.0* | 33795.820 | -1.444 | 0.171 | -8.450 | 3.93E-16 |
| *vicX* | 751.828 | 1.439 | 0.232 | 6.190 | 4.39E-09 |
| *M5005_Spy_0741* | 10.312 | -1.436 | 0.515 | -2.787 | 0.011899052 |
| *M5005_Spy_0560* | 21.329 | -1.436 | 0.400 | -3.591 | 0.00097204 |
| *M5005_Spy_1757* | 1539.859 | 1.434 | 0.260 | 5.512 | 2.11E-07 |
| *lplB* | 169.546 | -1.432 | 0.234 | -6.109 | 7.06E-09 |
| *M5005_Spy_0466* | 1017.320 | 1.432 | 0.210 | 6.830 | 7.67E-11 |
| *M5005_Spy_1411* | 211.104 | 1.430 | 0.248 | 5.765 | 5.17E-08 |
| *M5005_Spy_0613* | 671.451 | 1.429 | 0.234 | 6.117 | 6.78E-09 |
| *M5005_Spy_1101* | 497.827 | 1.428 | 0.170 | 8.385 | 6.79E-16 |
| *pstA* | 324.844 | 1.416 | 0.186 | 7.620 | 2.81E-13 |
| *M5005_Spy_1859* | 36.788 | -1.414 | 0.374 | -3.779 | 0.000494371 |
| *M5005_Spy_1604* | 4.439 | 1.407 | 0.675 | 2.085 | 0.067911325 |
| *rpmI* | 321.020 | -1.405 | 0.210 | -6.676 | 2.09E-10 |
| *M5005_Spy_1069* | 4040.359 | 1.398 | 0.222 | 6.288 | 2.44E-09 |
| *M5005_Spy_1325* | 4096.846 | -1.397 | 0.264 | -5.293 | 6.56E-07 |
| *M5005_Spy_0487* | 291.369 | -1.395 | 0.226 | -6.162 | 5.17E-09 |
| *plr* | 2278.323 | -1.394 | 0.219 | -6.367 | 1.50E-09 |
| *M5005_Spy_0093* | 187.343 | -1.393 | 0.264 | -5.283 | 6.91E-07 |
| *M5005_Spy_0270* | 1000.345 | 1.393 | 0.274 | 5.078 | 1.96E-06 |
| *M5005_Spy_0792* | 582.525 | 1.391 | 0.261 | 5.327 | 5.56E-07 |
| *ahpC* | 864.129 | -1.384 | 0.337 | -4.101 | 0.000147785 |
| *ldh* | 655.933 | -1.383 | 0.273 | -5.057 | 2.16E-06 |
| *pstS* | 260.039 | 1.382 | 0.175 | 7.902 | 3.34E-14 |
| *pbp2A* | 3808.740 | 1.378 | 0.291 | 4.745 | 9.46E-06 |
| *M5005_Spy_0344* | 27.356 | -1.374 | 0.386 | -3.564 | 0.001062011 |
| *smc* | 2836.007 | 1.374 | 0.161 | 8.519 | 2.21E-16 |
| *M5005_Spy_0098* | 45.955 | -1.373 | 0.402 | -3.415 | 0.001770839 |
| *M5005_Spy_1392* | 41.913 | -1.371 | 0.339 | -4.044 | 0.00018579 |
| *pyrD* | 54.401 | -1.371 | 0.279 | -4.909 | 4.45E-06 |
| *Gene or locus* | *Normalized count base mean* | *log2 fold change* | *Standard error* | *Wald test statistic* | *Benjamini-Hochberg adjusted P value* |
| *sic1.01* | 58971.752 | -1.371 | 0.175 | -7.828 | 5.86E-14 |
| *hutI* | 6.932 | 1.369 | 0.604 | 2.266 | 0.045215277 |
| *M5005_Spy_0085* | 132.873 | -1.367 | 0.213 | -6.423 | 1.07E-09 |
| *M5005_Spy_0851* | 477.320 | -1.362 | 0.286 | -4.756 | 9.01E-06 |
| *eno* | 3854.066 | -1.356 | 0.206 | -6.596 | 3.47E-10 |
| *M5005_Spy_1296* | 340.213 | -1.354 | 0.222 | -6.085 | 8.11E-09 |
| *M5005_Spy_1095* | 940.287 | 1.349 | 0.226 | 5.959 | 1.70E-08 |
| *M5005_Spy_0150* | 6.786 | 1.348 | 0.670 | 2.014 | NA |
| *M5005_Spy_0355* | 136.105 | -1.345 | 0.248 | -5.416 | 3.49E-07 |
| *gapN* | 3097.628 | -1.345 | 0.198 | -6.795 | 9.58E-11 |
| *trmD* | 307.890 | 1.343 | 0.207 | 6.488 | 7.04E-10 |
| *glpK* | 155.919 | -1.340 | 0.676 | -1.981 | NA |
| *grab* | 9.708 | -1.340 | 0.515 | -2.603 | 0.019622718 |
| *M5005_Spy_0449* | 128.778 | 1.339 | 0.225 | 5.945 | 1.84E-08 |
| *M5005_Spy_0680* | 1084.943 | 1.336 | 0.234 | 5.698 | 7.57E-08 |
| *M5005_Spy_0854* | 56.524 | -1.334 | 0.374 | -3.568 | 0.001049629 |
| *M5005_Spy_0976* | 590.693 | -1.332 | 0.178 | -7.467 | 8.86E-13 |
| *ntpF* | 30.118 | 1.325 | 0.355 | 3.729 | 0.000596583 |
| *M5005_Spy_0944* | 158.250 | -1.324 | 0.226 | -5.855 | 3.13E-08 |
| *M5005_Spy_0269* | 586.964 | -1.324 | 0.204 | -6.479 | 7.40E-10 |
| *pyrH* | 89.676 | -1.323 | 0.350 | -3.776 | 0.000499932 |
| *M5005_Spy_1242* | 323.236 | -1.317 | 0.172 | -7.658 | 2.14E-13 |
| *upp* | 383.330 | -1.314 | 0.216 | -6.076 | 8.48E-09 |
| *rpoA* | 1980.898 | -1.311 | 0.150 | -8.737 | 3.55E-17 |
| *udk* | 165.327 | -1.311 | 0.213 | -6.161 | 5.18E-09 |
| *satE* | 67.533 | 1.311 | 0.327 | 4.005 | 0.000215794 |
| *nusB* | 539.653 | -1.308 | 0.190 | -6.869 | 5.87E-11 |
| *corA* | 181.884 | -1.305 | 0.262 | -4.980 | 3.14E-06 |
| *lacR.2* | 375.257 | -1.304 | 0.331 | -3.945 | 0.00026839 |
| *M5005_Spy_0548* | 553.234 | -1.300 | 0.236 | -5.506 | 2.18E-07 |
| *araD* | 11.239 | 1.297 | 0.706 | 1.836 | NA |
| *argS* | 416.389 | -1.295 | 0.170 | -7.629 | 2.64E-13 |
| *M5005_Spy_0453* | 7.743 | 1.293 | 0.615 | 2.101 | 0.065751458 |
| *coaA* | 72.702 | -1.289 | 0.257 | -5.024 | 2.55E-06 |
| *M5005_Spy_1602* | 1485.044 | -1.289 | 0.144 | -8.942 | 6.04E-18 |
| *M5005_Spy_0718* | 6.185 | -1.283 | 0.584 | -2.199 | 0.052951032 |
| *mutY* | 249.477 | -1.282 | 0.309 | -4.154 | 0.000120528 |
| *pyrP* | 547.662 | 1.280 | 0.320 | 3.995 | 0.000222722 |
| *pstC* | 73.723 | 1.279 | 0.256 | 4.998 | 2.91E-06 |
| *rsuA* | 33.229 | -1.272 | 0.342 | -3.717 | 0.000618448 |
| *M5005_Spy_0463* | 2573.562 | 1.272 | 0.170 | 7.471 | 8.64E-13 |
| *M5005_Spy_1843* | 222.621 | -1.269 | 0.435 | -2.916 | 0.008277946 |
| *malM* | 139.388 | -1.269 | 0.268 | -4.727 | 1.03E-05 |
| *Gene or locus* | *Normalized count base mean* | *log2 fold change* | *Standard error* | *Wald test statistic* | *Benjamini-Hochberg adjusted P value* |
| *rplV* | 423.573 | -1.264 | 0.239 | -5.289 | 6.70E-07 |
| *M5005_Spy_0742* | 9.546 | -1.263 | 0.530 | -2.383 | 0.034232811 |
| *metK1* | 124.986 | 1.256 | 0.235 | 5.339 | 5.26E-07 |
| *manM* | 474.318 | 1.256 | 0.216 | 5.817 | 3.91E-08 |
| *pyk* | 1301.939 | -1.255 | 0.223 | -5.631 | 1.09E-07 |
| *gidA* | 3117.090 | 1.251 | 0.199 | 6.284 | 2.50E-09 |
| *rplO* | 4233.153 | 1.251 | 0.156 | 8.006 | 1.50E-14 |
| *rpsL* | 1129.376 | -1.247 | 0.198 | -6.308 | 2.17E-09 |
| *rpsK* | 513.259 | -1.245 | 0.257 | -4.848 | 5.98E-06 |
| *acoC* | 526.558 | -1.245 | 0.264 | -4.709 | 1.12E-05 |
| *M5005_Spy_0320* | 110.920 | -1.243 | 0.225 | -5.513 | 2.11E-07 |
| *scrR* | 25.659 | -1.238 | 0.387 | -3.198 | 0.003549198 |
| *pgi* | 638.032 | -1.237 | 0.186 | -6.642 | 2.61E-10 |
| *M5005_Spy_0296* | 145.881 | -1.236 | 0.197 | -6.279 | 2.57E-09 |
| *M5005_Spy_1295* | 231.062 | -1.234 | 0.235 | -5.255 | 7.98E-07 |
| *M5005_Spy_1797* | 271.625 | -1.233 | 0.305 | -4.050 | 0.000182223 |
| *M5005_Spy_1230* | 577.833 | 1.233 | 0.186 | 6.632 | 2.76E-10 |
| *M5005_Spy_0513* | 12.966 | 1.231 | 0.472 | 2.610 | 0.019245888 |
| *mraW* | 342.378 | -1.229 | 0.285 | -4.307 | 6.47E-05 |
| *M5005_Spy_1160* | 106.127 | 1.229 | 0.344 | 3.573 | 0.001038072 |
| *thrS* | 225.415 | -1.228 | 0.223 | -5.503 | 2.21E-07 |
| *rpsC* | 1773.300 | 1.225 | 0.230 | 5.320 | 5.75E-07 |
| *rpsM* | 3665.444 | 1.224 | 0.268 | 4.576 | 2.04E-05 |
| *rpsU* | 196.727 | -1.223 | 0.255 | -4.795 | 7.64E-06 |
| *M5005_Spy_0529* | 487.353 | 1.210 | 0.169 | 7.139 | 9.28E-12 |
| *glmS* | 1896.334 | -1.209 | 0.171 | -7.056 | 1.66E-11 |
| *salX* | 9.401 | 1.208 | 0.602 | 2.007 | 0.079349666 |
| *M5005_Spy_0117* | 40.367 | -1.207 | 0.442 | -2.734 | 0.013791964 |
| *M5005_Spy_1563* | 57.400 | -1.206 | 0.293 | -4.108 | 0.000143991 |
| *M5005_Spy_0991* | 405.380 | -1.202 | 0.151 | -7.958 | 2.20E-14 |
| *M5005_Spy_1641* | 3.738 | -1.197 | 0.656 | -1.825 | 0.11323761 |
| *pth* | 79.559 | -1.196 | 0.385 | -3.105 | 0.004743097 |
| *rpoE* | 2255.233 | -1.195 | 0.117 | -10.188 | 5.69E-23 |
| *srtF* | 65.758 | 1.194 | 0.346 | 3.456 | 0.001544742 |
| *M5005_Spy_1822* | 90.602 | 1.190 | 0.277 | 4.294 | 6.79E-05 |
| *nagA* | 711.587 | -1.189 | 0.206 | -5.766 | 5.16E-08 |
| *dexS* | 138.581 | -1.188 | 0.314 | -3.783 | 0.000489388 |
| *M5005_Spy_0766* | 473.624 | 1.188 | 0.220 | 5.401 | 3.78E-07 |
| *gmk* | 663.901 | -1.179 | 0.204 | -5.792 | 4.48E-08 |
| *metG* | 564.702 | 1.177 | 0.219 | 5.376 | 4.33E-07 |
| *M5005_Spy_0494* | 11.918 | -1.169 | 0.504 | -2.321 | 0.03974691 |
| *M5005_Spy_1597* | 468.300 | -1.168 | 0.225 | -5.197 | 1.07E-06 |
| *M5005_Spy_0493* | 40.308 | -1.164 | 0.303 | -3.837 | 0.000398235 |
| *Gene or locus* | *Normalized count base mean* | *log2 fold change* | *Standard error* | *Wald test statistic* | *Benjamini-Hochberg adjusted P value* |
| *cbiQ* | 493.628 | 1.156 | 0.324 | 3.564 | 0.001062011 |
| *M5005_Spy_1167* | 1471.387 | 1.155 | 0.214 | 5.396 | 3.88E-07 |
| *M5005_Spy_0178* | 152.006 | -1.148 | 0.190 | -6.052 | 9.86E-09 |
| *M5005_Spy_0440* | 14.969 | -1.147 | 0.497 | -2.305 | 0.041173851 |
| *M5005_Spy_1374* | 647.968 | -1.143 | 0.261 | -4.381 | 4.73E-05 |
| *gpsA* | 399.655 | -1.143 | 0.270 | -4.226 | 8.89E-05 |
| *rplJ* | 865.068 | -1.141 | 0.214 | -5.335 | 5.33E-07 |
| *ftsY* | 962.197 | 1.141 | 0.240 | 4.762 | 8.82E-06 |
| *fmt* | 413.063 | -1.141 | 0.204 | -5.581 | 1.44E-07 |
| *M5005_Spy_0528* | 112.815 | 1.139 | 0.283 | 4.026 | 0.000199522 |
| *luxS* | 48.287 | 1.137 | 0.323 | 3.523 | 0.001223916 |
| *trxB* | 637.985 | 1.137 | 0.237 | 4.802 | 7.42E-06 |
| *M5005_Spy_0609* | 1372.111 | 1.136 | 0.210 | 5.422 | 3.38E-07 |
| *silD* | 31.008 | 1.134 | 0.332 | 3.416 | 0.00176454 |
| *M5005_Spy_1378* | 138.493 | -1.131 | 0.357 | -3.168 | 0.003895258 |
| *M5005_Spy_0634* | 808.036 | -1.129 | 0.199 | -5.672 | 8.72E-08 |
| *M5005_Spy_0743* | 214.077 | 1.125 | 0.172 | 6.529 | 5.37E-10 |
| *M5005_Spy_0834* | 107.610 | -1.125 | 0.253 | -4.437 | 3.72E-05 |
| *M5005_Spy_1112* | 311.995 | -1.124 | 0.169 | -6.664 | 2.26E-10 |
| *M5005_Spy_0719* | 40.512 | -1.124 | 0.342 | -3.285 | 0.00271388 |
| *trmE* | 88.775 | -1.123 | 0.306 | -3.669 | 0.000737279 |
| *recU* | 182.639 | -1.123 | 0.218 | -5.149 | 1.37E-06 |
| *glnH* | 1461.353 | 1.121 | 0.270 | 4.157 | 0.000119204 |
| *M5005_Spy_0496* | 117.310 | -1.121 | 0.273 | -4.103 | 0.000146995 |
| *adhA* | 1344.654 | -1.119 | 0.221 | -5.058 | 2.15E-06 |
| *M5005_Spy_1396* | 473.568 | 1.119 | 0.256 | 4.371 | 4.92E-05 |
| *glpO* | 185.674 | -1.117 | 0.699 | -1.599 | NA |
| *recN* | 1405.968 | 1.117 | 0.160 | 6.992 | 2.59E-11 |
| *M5005_Spy_0385* | 914.552 | -1.116 | 0.232 | -4.820 | 6.87E-06 |
| *M5005_Spy_0042* | 38.795 | 1.116 | 0.383 | 2.916 | 0.008277946 |
| *rpoD* | 422.013 | -1.112 | 0.169 | -6.593 | 3.53E-10 |
| *M5005_Spy_0163* | 2827.702 | 1.109 | 0.202 | 5.483 | 2.47E-07 |
| *pepXP* | 303.965 | -1.107 | 0.215 | -5.146 | 1.39E-06 |
| *glyS* | 707.797 | 1.107 | 0.203 | 5.452 | 2.91E-07 |
| *grpE* | 3338.192 | -1.100 | 0.260 | -4.235 | 8.62E-05 |
| *M5005_Spy_0144* | 6.757 | -1.100 | 0.614 | -1.793 | 0.120152518 |
| *M5005_Spy_1254* | 182.426 | -1.100 | 0.282 | -3.903 | 0.000315106 |
| *M5005_Spy_1755* | 81.110 | -1.099 | 0.230 | -4.784 | 8.00E-06 |
| *nrdD* | 4313.343 | -1.099 | 0.231 | -4.760 | 8.88E-06 |
| *srtK* | 1041.521 | 1.098 | 0.285 | 3.856 | 0.000373031 |
| *M5005_Spy_1567* | 184.120 | -1.097 | 0.251 | -4.366 | 5.01E-05 |
| *lysS* | 317.836 | -1.095 | 0.212 | -5.169 | 1.24E-06 |
| *rpsO* | 136.077 | -1.093 | 0.243 | -4.496 | 2.91E-05 |
| *Gene or locus* | *Normalized count base mean* | *log2 fold change* | *Standard error* | *Wald test statistic* | *Benjamini-Hochberg adjusted P value* |
| *yvqF* | 179.393 | 1.093 | 0.228 | 4.793 | 7.68E-06 |
| *comYA* | 2.339 | 1.092 | 0.707 | 1.544 | 0.187509016 |
| *holA* | 295.149 | 1.090 | 0.238 | 4.580 | 2.01E-05 |
| *pyrG* | 43.508 | -1.088 | 0.314 | -3.472 | 0.00147074 |
| *M5005_Spy_0179* | 125.885 | -1.085 | 0.226 | -4.801 | 7.43E-06 |
| *M5005_Spy_0164* | 5201.150 | 1.084 | 0.247 | 4.383 | 4.70E-05 |
| *M5005_Spy_0871* | 327.999 | 1.083 | 0.230 | 4.705 | 1.14E-05 |
| *M5005_Spy_1293* | 59.593 | -1.083 | 0.315 | -3.437 | 0.001648035 |
| *M5005_Spy_1377* | 188.972 | -1.083 | 0.461 | -2.348 | 0.037223543 |
| *folD* | 81.723 | -1.081 | 0.305 | -3.539 | 0.001156349 |
| *M5005_Spy_1321* | 630.233 | 1.079 | 0.232 | 4.647 | 1.49E-05 |
| *M5005_Spy_0786* | 188.340 | 1.078 | 0.219 | 4.929 | 4.07E-06 |
| *licT* | 23.552 | -1.078 | 0.391 | -2.758 | 0.012859419 |
| *M5005_Spy_0636* | 92.221 | -1.076 | 0.241 | -4.471 | 3.22E-05 |
| *M5005_Spy_1824* | 28.707 | -1.075 | 0.329 | -3.264 | 0.002897852 |
| *M5005_Spy_0422* | 349.797 | -1.074 | 0.209 | -5.126 | 1.53E-06 |
| *gpmA* | 500.885 | -1.072 | 0.157 | -6.818 | 8.25E-11 |
| *M5005_Spy_0878* | 1732.904 | -1.071 | 0.319 | -3.359 | 0.002137509 |
| *M5005_Spy_0966* | 170.295 | 1.069 | 0.237 | 4.509 | 2.77E-05 |
| *M5005_Spy_0939* | 705.299 | 1.065 | 0.301 | 3.543 | 0.001142562 |
| *mscL* | 41.632 | -1.062 | 0.353 | -3.005 | 0.006404369 |
| *sloR* | 176.065 | 1.058 | 0.230 | 4.607 | 1.79E-05 |
| *guaC* | 48.408 | -1.057 | 0.378 | -2.798 | 0.011547887 |
| *rplN* | 1790.938 | 1.055 | 0.169 | 6.244 | 3.14E-09 |
| *M5005_Spy_1716* | 63.792 | -1.054 | 0.255 | -4.129 | 0.00013271 |
| *M5005_Spy_0881* | 40.606 | -1.054 | 0.323 | -3.266 | 0.002875519 |
| *dnaJ* | 893.600 | -1.053 | 0.290 | -3.627 | 0.00085179 |
| *M5005_Spy_0311* | 113.594 | 1.052 | 0.263 | 4.001 | 0.000218387 |
| *clpP* | 3333.473 | 1.051 | 0.279 | 3.761 | 0.000528441 |
| *M5005_Spy_0971* | 4005.515 | -1.048 | 0.243 | -4.313 | 6.29E-05 |
| *pstI* | 1404.956 | -1.046 | 0.149 | -6.998 | 2.50E-11 |
| *hemK* | 397.493 | 1.038 | 0.180 | 5.775 | 4.93E-08 |
| *M5005_Spy_0626* | 267.908 | -1.037 | 0.198 | -5.229 | 9.18E-07 |
| *M5005_Spy_0845* | 242.939 | -1.036 | 0.292 | -3.552 | 0.001106759 |
| *M5005_Spy_1097* | 255.070 | 1.035 | 0.217 | 4.768 | 8.62E-06 |
| *glpF* | 68.075 | -1.032 | 0.708 | -1.458 | NA |
| *fruR* | 1969.114 | -1.031 | 0.363 | -2.845 | 0.010092568 |
| *M5005_Spy_1111* | 91.344 | -1.030 | 0.231 | -4.456 | 3.46E-05 |
| *M5005_Spy_0433* | 18.162 | 1.029 | 0.434 | 2.369 | 0.03526639 |
| *M5005_Spy_1113* | 113.643 | -1.029 | 0.293 | -3.514 | 0.001258968 |
| *M5005_Spy_1749* | 42.523 | -1.028 | 0.283 | -3.632 | 0.000839027 |
| *uviB* | 38.881 | -1.028 | 0.390 | -2.633 | 0.018175675 |
| *M5005_Spy_1105* | 135.053 | -1.027 | 0.262 | -3.923 | 0.000291958 |
| *Gene or locus* | *Normalized count base mean* | *log2 fold change* | *Standard error* | *Wald test statistic* | *Benjamini-Hochberg adjusted P value* |
| *typA* | 974.977 | -1.024 | 0.155 | -6.603 | 3.32E-10 |
| *rplB* | 773.559 | -1.024 | 0.140 | -7.321 | 2.51E-12 |
| *purA* | 374.981 | -1.020 | 0.240 | -4.257 | 7.88E-05 |
| *M5005_Spy_1227* | 2619.871 | 1.013 | 0.171 | 5.937 | 1.92E-08 |
| *M5005_Spy_0747* | 2497.051 | 1.013 | 0.227 | 4.453 | 3.50E-05 |
| *mtsB* | 225.529 | -1.012 | 0.235 | -4.301 | 6.58E-05 |
| *M5005_Spy_0483* | 727.833 | -1.009 | 0.210 | -4.811 | 7.17E-06 |
| *M5005_Spy_0101* | 365.405 | 1.006 | 0.224 | 4.502 | 2.85E-05 |
| *lplA* | 643.667 | 1.006 | 0.273 | 3.684 | 0.00069764 |
| *M5005_Spy_1369* | 31.179 | 1.005 | 0.355 | 2.828 | 0.010604163 |
| *M5005_Spy_1078* | 1.944 | 1.003 | 0.706 | 1.422 | NA |
| *codY* | 1333.761 | -1.000 | 0.164 | -6.081 | 8.31E-09 |
| *pstB* | 624.995 | 0.996 | 0.194 | 5.137 | 1.45E-06 |
| *pstB2* | 167.074 | 0.995 | 0.224 | 4.438 | 3.70E-05 |
| *M5005_Spy_1258* | 186.750 | -0.993 | 0.304 | -3.266 | 0.002875519 |
| *M5005_Spy_0671* | 647.370 | -0.992 | 0.224 | -4.434 | 3.77E-05 |
| *M5005_Spy_0702* | 317.861 | -0.990 | 0.239 | -4.139 | 0.000127579 |
| *alaS* | 524.052 | -0.990 | 0.223 | -4.446 | 3.59E-05 |
| *M5005_Spy_1863* | 1.945 | 0.988 | 0.707 | 1.398 | NA |
| *M5005_Spy_0721* | 158.302 | -0.987 | 0.247 | -4.002 | 0.000218344 |
| *M5005_Spy_0847* | 356.813 | -0.980 | 0.235 | -4.173 | 0.000111866 |
| *dppD* | 372.878 | 0.979 | 0.192 | 5.090 | 1.85E-06 |
| *pppL* | 1431.335 | 0.979 | 0.221 | 4.426 | 3.89E-05 |
| *cpsY* | 146.540 | -0.977 | 0.248 | -3.939 | 0.000274114 |
| *M5005_Spy_1810* | 517.178 | 0.977 | 0.143 | 6.821 | 8.12E-11 |
| *engB* | 1060.311 | 0.974 | 0.195 | 4.987 | 3.05E-06 |
| *M5005_Spy_0914* | 56.078 | -0.973 | 0.327 | -2.971 | 0.007090183 |
| *M5005_Spy_1694* | 184.602 | -0.973 | 0.216 | -4.509 | 2.77E-05 |
| *M5005_Spy_1351* | 933.149 | 0.971 | 0.178 | 5.441 | 3.05E-07 |
| *sdhB* | 147.445 | -0.969 | 0.212 | -4.563 | 2.17E-05 |
| *M5005_Spy_0770* | 130.178 | 0.969 | 0.276 | 3.510 | 0.001276779 |
| *ctsR* | 275.030 | -0.968 | 0.269 | -3.600 | 0.000940413 |
| *ntpB* | 104.931 | 0.965 | 0.251 | 3.838 | 0.000398172 |
| *srtI* | 0.725 | 0.964 | 0.643 | 1.498 | NA |
| *dnaK* | 11067.483 | 0.963 | 0.234 | 4.114 | 0.000140891 |
| *deoD* | 423.292 | -0.961 | 0.227 | -4.230 | 8.75E-05 |
| *ahpF* | 1290.532 | -0.960 | 0.286 | -3.352 | 0.002187461 |
| *purK* | 77.492 | -0.959 | 0.283 | -3.383 | 0.001972202 |
| *M5005_Spy_0426* | 716.038 | -0.957 | 0.205 | -4.675 | 1.30E-05 |
| *M5005_Spy_0876* | 109.604 | 0.957 | 0.241 | 3.976 | 0.000237722 |
| *clpC* | 1809.189 | 0.955 | 0.204 | 4.688 | 1.23E-05 |
| *serS* | 322.711 | -0.954 | 0.199 | -4.787 | 7.88E-06 |
| *rplS* | 508.463 | -0.953 | 0.138 | -6.881 | 5.47E-11 |
| *Gene or locus* | *Normalized count base mean* | *log2 fold change* | *Standard error* | *Wald test statistic* | *Benjamini-Hochberg adjusted P value* |
| *oadB* | 31.731 | 0.951 | 0.373 | 2.553 | 0.022486346 |
| *M5005_Spy_0273* | 189.436 | 0.951 | 0.225 | 4.233 | 8.68E-05 |
| *pheS* | 465.112 | 0.949 | 0.194 | 4.903 | 4.57E-06 |
| *rplL* | 1167.030 | -0.948 | 0.143 | -6.637 | 2.69E-10 |
| *phr* | 151.539 | -0.946 | 0.234 | -4.051 | 0.000181382 |
| *M5005_Spy_1613* | 67.612 | -0.942 | 0.323 | -2.917 | 0.008268902 |
| *mefE* | 90.244 | 0.942 | 0.283 | 3.326 | 0.002375691 |
| *exoA* | 47.666 | -0.940 | 0.333 | -2.818 | 0.010903299 |
| *proC* | 166.808 | -0.939 | 0.222 | -4.232 | 8.71E-05 |
| *M5005_Spy_0343* | 17.953 | -0.938 | 0.426 | -2.202 | 0.052547706 |
| *M5005_Spy_0041* | 98.605 | 0.936 | 0.218 | 4.290 | 6.90E-05 |
| *M5005_Spy_1274* | 1335.712 | -0.936 | 0.235 | -3.980 | 0.000234524 |
| *M5005_Spy_0885* | 1080.293 | 0.934 | 0.201 | 4.660 | 1.40E-05 |
| *M5005_Spy_0902* | 37.575 | 0.934 | 0.338 | 2.765 | 0.012668367 |
| *htrA* | 5573.909 | -0.934 | 0.281 | -3.328 | 0.002359582 |
| *hslO* | 103.540 | -0.934 | 0.292 | -3.200 | 0.003528734 |
| *nifS2* | 422.762 | -0.932 | 0.255 | -3.656 | 0.000773381 |
| *dnaQ* | 84.644 | 0.931 | 0.322 | 2.887 | 0.009026689 |
| *fba* | 1924.815 | -0.929 | 0.154 | -6.026 | 1.14E-08 |
| *isp* | 1597.467 | 0.928 | 0.147 | 6.335 | 1.84E-09 |
| *M5005_Spy_1151* | 94.983 | 0.928 | 0.270 | 3.437 | 0.001648035 |
| *dinP* | 72.221 | -0.926 | 0.307 | -3.019 | 0.006152028 |
| *deoB* | 533.180 | -0.923 | 0.206 | -4.492 | 2.96E-05 |
| *M5005_Spy_0965* | 148.826 | 0.923 | 0.259 | 3.565 | 0.00105913 |
| *M5005_Spy_0638* | 255.947 | 0.922 | 0.223 | 4.137 | 0.000127964 |
| *aapA* | 33.068 | -0.922 | 0.328 | -2.814 | 0.011031653 |
| *M5005_Spy_1360* | 823.070 | 0.921 | 0.193 | 4.773 | 8.41E-06 |
| *fabH* | 2932.985 | -0.920 | 0.166 | -5.551 | 1.71E-07 |
| *M5005_Spy_0180* | 72.428 | -0.920 | 0.281 | -3.270 | 0.00284413 |
| *malC* | 3860.548 | 0.918 | 0.384 | 2.392 | 0.033498024 |
| *M5005_Spy_1163* | 259.947 | 0.918 | 0.241 | 3.810 | 0.000441931 |
| *mccF* | 1654.892 | 0.916 | 0.139 | 6.607 | 3.26E-10 |
| *M5005_Spy_0149* | 4.856 | 0.916 | 0.688 | 1.332 | NA |
| *endA* | 52.163 | -0.915 | 0.356 | -2.575 | 0.021209806 |
| *M5005_Spy_1527* | 118.515 | 0.915 | 0.309 | 2.962 | 0.00726911 |
| *srv* | 117.684 | -0.915 | 0.281 | -3.261 | 0.002918816 |
| *M5005_Spy_0262* | 399.096 | 0.915 | 0.192 | 4.759 | 8.94E-06 |
| *pyrB* | 735.360 | 0.911 | 0.252 | 3.616 | 0.000885029 |
| *M5005_Spy_0897* | 44.463 | 0.911 | 0.366 | 2.490 | 0.026392338 |
| *def* | 266.406 | -0.910 | 0.228 | -3.996 | 0.000221738 |
| *asnC* | 431.535 | -0.908 | 0.209 | -4.341 | 5.57E-05 |
| *arsC* | 141.568 | -0.908 | 0.220 | -4.119 | 0.000137813 |
| *M5005_Spy_0555* | 21.489 | -0.908 | 0.380 | -2.392 | 0.033498024 |
| *Gene or locus* | *Normalized count base mean* | *log2 fold change* | *Standard error* | *Wald test statistic* | *Benjamini-Hochberg adjusted P value* |
| *gidB* | 89.377 | -0.906 | 0.256 | -3.546 | 0.001132771 |
| *M5005_Spy_1330* | 351.166 | -0.905 | 0.181 | -4.987 | 3.05E-06 |
| *copA* | 1154.565 | -0.905 | 0.293 | -3.083 | 0.005052211 |
| *phoH* | 177.432 | -0.905 | 0.321 | -2.821 | 0.010831266 |
| *M5005_Spy_1094* | 218.519 | 0.904 | 0.337 | 2.686 | 0.015677661 |
| *M5005_Spy_0552* | 122.235 | -0.903 | 0.284 | -3.176 | 0.003797222 |
| *M5005_Spy_0746* | 297.336 | 0.902 | 0.174 | 5.198 | 1.07E-06 |
| *lacD.1* | 828.357 | 0.902 | 0.151 | 5.954 | 1.75E-08 |
| *glgP* | 150.572 | -0.902 | 0.287 | -3.140 | 0.004245665 |
| *salB* | 23.104 | 0.901 | 0.373 | 2.419 | 0.031567829 |
| *M5005_Spy_0759* | 222.006 | 0.901 | 0.224 | 4.030 | 0.000196196 |
| *M5005_Spy_1692* | 249.712 | 0.900 | 0.370 | 2.431 | 0.030649612 |
| *rplU* | 209.478 | -0.900 | 0.225 | -3.993 | 0.000223907 |
| *M5005_Spy_0216* | 38.974 | 0.898 | 0.419 | 2.144 | 0.060127606 |
| *M5005_Spy_0306* | 396.281 | 0.898 | 0.210 | 4.269 | 7.52E-05 |
| *ptsH* | 1799.420 | -0.892 | 0.221 | -4.046 | 0.000184737 |
| *rpsA* | 1408.839 | -0.891 | 0.171 | -5.220 | 9.57E-07 |
| *M5005_Spy_1688* | 4.015 | -0.888 | 0.652 | -1.362 | 0.250733207 |
| *glmU* | 805.470 | -0.887 | 0.233 | -3.810 | 0.000441931 |
| *M5005_Spy_1127* | 1.169 | 0.884 | 0.697 | 1.268 | NA |
| *M5005_Spy_0300* | 1149.495 | -0.883 | 0.286 | -3.093 | 0.0049059 |
| *rpsN* | 106.005 | -0.883 | 0.259 | -3.410 | 0.001801118 |
| *M5005_Spy_1473* | 127.511 | -0.883 | 0.267 | -3.308 | 0.002511609 |
| *pmi* | 197.641 | -0.881 | 0.191 | -4.612 | 1.75E-05 |
| *rnpA* | 124.150 | -0.876 | 0.245 | -3.580 | 0.001010393 |
| *rmlD* | 230.060 | -0.876 | 0.178 | -4.923 | 4.16E-06 |
| *M5005_Spy_1302* | 145.560 | -0.876 | 0.228 | -3.843 | 0.000391097 |
| *M5005_Spy_0940* | 893.565 | 0.874 | 0.220 | 3.975 | 0.000238235 |
| *M5005_Spy_0829* | 1103.453 | 0.870 | 0.222 | 3.916 | 0.000299782 |
| *M5005_Spy_0856* | 442.081 | 0.870 | 0.209 | 4.166 | 0.000115116 |
| *infA* | 1403.046 | -0.867 | 0.130 | -6.694 | 1.85E-10 |
| *M5005_Spy_1584* | 167.635 | 0.864 | 0.216 | 4.001 | 0.000218475 |
| *oppD* | 1005.063 | 0.862 | 0.270 | 3.193 | 0.003602015 |
| *M5005_Spy_0669* | 30.655 | 0.861 | 0.386 | 2.233 | 0.048795778 |
| *mvaS.1* | 190.146 | -0.860 | 0.322 | -2.674 | 0.016192984 |
| *srtA* | 41.418 | 0.860 | 0.350 | 2.456 | 0.028693339 |
| *M5005_Spy_1731* | 13.310 | -0.858 | 0.445 | -1.929 | 0.092314581 |
| *dut* | 156.562 | -0.857 | 0.227 | -3.780 | 0.000493984 |
| *nrdE.2* | 897.832 | -0.856 | 0.174 | -4.909 | 4.45E-06 |
| *ccdA* | 8.542 | 0.852 | 0.635 | 1.342 | 0.258262597 |
| *M5005_Spy_0313* | 31.837 | -0.852 | 0.325 | -2.618 | 0.018887286 |
| *rgpDc* | 1268.136 | 0.849 | 0.161 | 5.275 | 7.16E-07 |
| *M5005_Spy_0992* | 405.647 | -0.846 | 0.163 | -5.204 | 1.04E-06 |
| *Gene or locus* | *Normalized count base mean* | *log2 fold change* | *Standard error* | *Wald test statistic* | *Benjamini-Hochberg adjusted P value* |
| *M5005_Spy_0970* | 314.800 | -0.846 | 0.188 | -4.495 | 2.91E-05 |
| *acpS* | 238.021 | 0.845 | 0.184 | 4.592 | 1.91E-05 |
| *M5005_Spy_0875* | 575.732 | 0.845 | 0.270 | 3.131 | 0.004367773 |
| *M5005_Spy_0833* | 9.491 | -0.844 | 0.614 | -1.375 | NA |
| *prmA* | 177.103 | -0.844 | 0.188 | -4.484 | 3.06E-05 |
| *rpmA* | 348.309 | -0.843 | 0.187 | -4.508 | 2.78E-05 |
| *M5005_Spy_1806* | 38.942 | -0.843 | 0.338 | -2.492 | 0.0263028 |
| *M5005_Spy_0549* | 19.174 | -0.843 | 0.400 | -2.104 | 0.065405736 |
| *M5005_Spy_1504* | 105.697 | 0.841 | 0.225 | 3.730 | 0.000595183 |
| *M5005_Spy_1578* | 5.753 | -0.838 | 0.645 | -1.299 | 0.275211084 |
| *hit* | 86.582 | -0.836 | 0.261 | -3.201 | 0.003528734 |
| *M5005_Spy_1746* | 9.289 | 0.835 | 0.505 | 1.652 | 0.155192666 |
| *lacE* | 408.805 | 0.834 | 0.156 | 5.363 | 4.63E-07 |
| *M5005_Spy_0398* | 86.875 | 0.831 | 0.228 | 3.647 | 0.000793859 |
| *aroK* | 109.059 | 0.831 | 0.214 | 3.877 | 0.000347552 |
| *dnaD* | 736.671 | 0.830 | 0.191 | 4.342 | 5.56E-05 |
| *M5005_Spy_0388* | 581.483 | 0.828 | 0.214 | 3.868 | 0.000359569 |
| *groEL* | 5067.982 | -0.827 | 0.255 | -3.244 | 0.003066013 |
| *M5005_Spy_0590* | 40.052 | 0.827 | 0.325 | 2.541 | 0.023133901 |
| *M5005_Spy_0623* | 619.518 | -0.824 | 0.232 | -3.552 | 0.001106759 |
| *ptsD* | 893.426 | 0.824 | 0.234 | 3.519 | 0.001238795 |
| *acpP* | 445.644 | -0.824 | 0.276 | -2.985 | 0.006823457 |
| *yvqC* | 543.611 | 0.823 | 0.211 | 3.902 | 0.000316183 |
| *elaC* | 330.715 | 0.823 | 0.168 | 4.903 | 4.57E-06 |
| *M5005_Spy_0868* | 358.936 | 0.823 | 0.207 | 3.970 | 0.000242852 |
| *M5005_Spy_1652* | 657.037 | 0.817 | 0.203 | 4.023 | 0.000201047 |
| *M5005_Spy_0502* | 28.186 | -0.812 | 0.422 | -1.922 | 0.09325498 |
| *M5005_Spy_0357* | 674.657 | 0.812 | 0.227 | 3.568 | 0.001049629 |
| *M5005_Spy_0744* | 16.318 | 0.810 | 0.421 | 1.927 | 0.092567675 |
| *fbp* | 71.961 | -0.809 | 0.281 | -2.876 | 0.009279499 |
| *M5005_Spy_0420* | 56.376 | -0.806 | 0.340 | -2.371 | 0.035231793 |
| *speJ* | 557.294 | 0.806 | 0.278 | 2.895 | 0.008810588 |
| *M5005_Spy_0994* | 404.569 | -0.805 | 0.202 | -3.986 | 0.00023058 |
| *M5005_Spy_1528* | 227.700 | 0.799 | 0.191 | 4.179 | 0.000108871 |
| *M5005_Spy_1315* | 23.239 | -0.798 | 0.402 | -1.985 | 0.082567124 |
| *yajC* | 207.778 | -0.796 | 0.211 | -3.780 | 0.000494213 |
| *guaA* | 738.367 | 0.796 | 0.196 | 4.056 | 0.000178196 |
| *thiI* | 224.406 | -0.791 | 0.217 | -3.650 | 0.000790297 |
| *comFC* | 3.594 | -0.791 | 0.660 | -1.198 | 0.320013483 |
| *M5005_Spy_1714* | 1893.724 | -0.789 | 0.212 | -3.720 | 0.000614674 |
| *ntpK* | 20.738 | 0.789 | 0.427 | 1.847 | 0.108214278 |
| *M5005_Spy_0028* | 5.509 | 0.787 | 0.650 | 1.211 | 0.31561573 |
| *M5005_Spy_1390* | 459.478 | -0.785 | 0.234 | -3.352 | 0.002187461 |
| *Gene or locus* | *Normalized count base mean* | *log2 fold change* | *Standard error* | *Wald test statistic* | *Benjamini-Hochberg adjusted P value* |
| *asnB* | 95.273 | -0.784 | 0.263 | -2.984 | 0.006823457 |
| *pepQ* | 492.849 | -0.784 | 0.264 | -2.975 | 0.007004565 |
| *glnA* | 1743.245 | -0.784 | 0.148 | -5.304 | 6.23E-07 |
| *dexB* | 68.324 | -0.782 | 0.278 | -2.817 | 0.010928869 |
| *endoS* | 427.200 | 0.781 | 0.236 | 3.316 | 0.002452972 |
| *citC* | 265.592 | -0.780 | 0.162 | -4.805 | 7.35E-06 |
| *trsA* | 133.874 | -0.780 | 0.307 | -2.539 | 0.023247057 |
| *atpH* | 361.828 | -0.779 | 0.237 | -3.288 | 0.002689948 |
| *M5005_Spy_1558* | 16.772 | -0.779 | 0.411 | -1.898 | 0.097851813 |
| *M5005_Spy_1348* | 61.267 | 0.777 | 0.287 | 2.707 | 0.01484855 |
| *M5005_Spy_1827* | 195.002 | 0.775 | 0.252 | 3.078 | 0.005126022 |
| *pepA* | 238.340 | -0.774 | 0.264 | -2.934 | 0.007873407 |
| *mreA* | 1156.659 | 0.773 | 0.142 | 5.458 | 2.83E-07 |
| *spd* | 1632.650 | -0.772 | 0.307 | -2.518 | 0.024520121 |
| *M5005_Spy_0771* | 52.792 | 0.771 | 0.268 | 2.873 | 0.009346747 |
| *M5005_Spy_1571* | 1508.353 | 0.769 | 0.208 | 3.700 | 0.0006596 |
| *M5005_Spy_1693* | 36.226 | -0.768 | 0.402 | -1.908 | 0.095745673 |
| *cinA* | 362.888 | 0.767 | 0.190 | 4.032 | 0.000195061 |
| *M5005_Spy_1727* | 250.700 | 0.767 | 0.163 | 4.717 | 1.08E-05 |
| *M5005_Spy_1289* | 819.367 | 0.766 | 0.238 | 3.215 | 0.003369787 |
| *M5005_Spy_0869* | 226.821 | 0.764 | 0.203 | 3.773 | 0.000505718 |
| *folK* | 1605.080 | 0.762 | 0.157 | 4.858 | 5.71E-06 |
| *M5005_Spy_0135* | 313.478 | -0.761 | 0.220 | -3.461 | 0.001519665 |
| *M5005_Spy_0310* | 417.743 | 0.760 | 0.160 | 4.745 | 9.45E-06 |
| *rplR* | 1514.806 | 0.760 | 0.219 | 3.468 | 0.001488632 |
| *M5005_Spy_0497* | 24.590 | -0.759 | 0.369 | -2.056 | 0.072290469 |
| *M5005_Spy_0842* | 365.460 | -0.759 | 0.229 | -3.314 | 0.002467842 |
| *lgt* | 517.612 | -0.758 | 0.186 | -4.077 | 0.000163462 |
| *M5005_Spy_1373* | 1118.823 | -0.758 | 0.215 | -3.519 | 0.001238795 |
| *salY* | 55.028 | 0.758 | 0.296 | 2.563 | 0.021859848 |
| *M5005_Spy_0647* | 1579.128 | 0.756 | 0.240 | 3.145 | 0.004184351 |
| *M5005_Spy_0932* | 555.325 | 0.756 | 0.233 | 3.247 | 0.003042197 |
| *M5005_Spy_0197* | 55.661 | 0.754 | 0.273 | 2.766 | 0.01265547 |
| *ntpC* | 60.553 | 0.752 | 0.297 | 2.530 | 0.023764491 |
| *M5005_Spy_1383* | 124.335 | 0.750 | 0.263 | 2.851 | 0.009920362 |
| *comYC* | 1.122 | -0.750 | 0.644 | -1.164 | NA |
| *M5005_Spy_0272* | 452.861 | 0.749 | 0.167 | 4.491 | 2.96E-05 |
| *M5005_Spy_0277* | 180.035 | -0.748 | 0.265 | -2.825 | 0.010684473 |
| *M5005_Spy_1525* | 487.903 | 0.748 | 0.194 | 3.862 | 0.000365635 |
| *murE* | 262.657 | -0.746 | 0.237 | -3.154 | 0.004063676 |
| *M5005_Spy_0589* | 27.305 | -0.746 | 0.357 | -2.091 | 0.067193618 |
| *rpmE2* | 1204.973 | -0.745 | 0.311 | -2.394 | 0.033449788 |
| *punA* | 507.588 | -0.744 | 0.259 | -2.876 | 0.009279499 |
| *Gene or locus* | *Normalized count base mean* | *log2 fold change* | *Standard error* | *Wald test statistic* | *Benjamini-Hochberg adjusted P value* |
| *M5005_Spy_0616* | 4.170 | 0.742 | 0.641 | 1.157 | 0.337616166 |
| *atpG* | 817.815 | -0.741 | 0.164 | -4.521 | 2.64E-05 |
| *M5005_Spy_0434* | 148.079 | -0.740 | 0.308 | -2.402 | 0.032850794 |
| *M5005_Spy_0717* | 828.541 | -0.740 | 0.207 | -3.572 | 0.001040296 |
| *M5005_Spy_0980* | 242.967 | 0.738 | 0.168 | 4.384 | 4.70E-05 |
| *M5005_Spy_0760* | 265.728 | -0.737 | 0.299 | -2.469 | 0.027931522 |
| *M5005_Spy_1353* | 153.753 | 0.737 | 0.273 | 2.702 | 0.015051363 |
| *M5005_Spy_0523* | 1.697 | 0.736 | 0.705 | 1.044 | NA |
| *M5005_Spy_1413* | 245.481 | -0.733 | 0.257 | -2.854 | 0.009843774 |
| *csp* | 198.827 | -0.731 | 0.314 | -2.330 | 0.038896434 |
| *M5005_Spy_1316* | 56.439 | -0.730 | 0.350 | -2.086 | 0.067851571 |
| *M5005_Spy_0134* | 331.794 | -0.728 | 0.185 | -3.944 | 0.000269107 |
| *mga* | 936.215 | 0.728 | 0.176 | 4.143 | 0.000125882 |
| *M5005_Spy_0022* | 21.218 | -0.726 | 0.431 | -1.685 | 0.146966635 |
| *dppC* | 270.186 | 0.725 | 0.166 | 4.376 | 4.83E-05 |
| *M5005_Spy_1594* | 69.002 | -0.721 | 0.343 | -2.104 | 0.065405736 |
| *scpB* | 394.420 | 0.719 | 0.167 | 4.318 | 6.18E-05 |
| *M5005_Spy_0214* | 60.307 | 0.719 | 0.423 | 1.702 | 0.142372551 |
| *M5005_Spy_1263* | 11.317 | -0.717 | 0.465 | -1.542 | 0.187852182 |
| *M5005_Spy_1730* | 247.968 | -0.716 | 0.180 | -3.985 | 0.000230936 |
| *leuS* | 236.852 | -0.715 | 0.181 | -3.950 | 0.000263577 |
| *M5005_Spy_1513* | 746.779 | -0.715 | 0.225 | -3.178 | 0.003774528 |
| *nusA* | 463.603 | -0.714 | 0.192 | -3.720 | 0.000614729 |
| *M5005_Spy_0672* | 939.990 | -0.714 | 0.166 | -4.302 | 6.56E-05 |
| *manN* | 242.223 | 0.713 | 0.172 | 4.142 | 0.000125911 |
| *M5005_Spy_0859* | 166.083 | 0.713 | 0.193 | 3.694 | 0.00067422 |
| *M5005_Spy_0870* | 287.868 | 0.712 | 0.190 | 3.744 | 0.000563758 |
| *M5005_Spy_0956* | 236.127 | 0.711 | 0.214 | 3.331 | 0.00234434 |
| *M5005_Spy_0212* | 45.124 | -0.711 | 0.406 | -1.752 | 0.129874172 |
| *hutG* | 92.995 | -0.710 | 0.223 | -3.186 | 0.00368106 |
| *guaB* | 963.923 | 0.710 | 0.160 | 4.450 | 3.53E-05 |
| *pcrA* | 1128.117 | 0.709 | 0.162 | 4.381 | 4.73E-05 |
| *mecA* | 329.308 | -0.708 | 0.162 | -4.374 | 4.85E-05 |
| *M5005_Spy_0275* | 354.141 | 0.708 | 0.219 | 3.238 | 0.003126502 |
| *mur1.2* | 149.343 | -0.707 | 0.320 | -2.211 | 0.051419761 |
| *M5005_Spy_1792* | 197.514 | -0.701 | 0.212 | -3.309 | 0.002511402 |
| *M5005_Spy_1279* | 197.274 | 0.700 | 0.181 | 3.867 | 0.000360255 |
| *accA* | 1543.663 | 0.699 | 0.164 | 4.268 | 7.53E-05 |
| *cfa* | 27.528 | -0.698 | 0.343 | -2.034 | 0.075316112 |
| *nupC* | 746.662 | -0.697 | 0.301 | -2.319 | 0.039953532 |
| *dnaA* | 559.997 | -0.696 | 0.179 | -3.882 | 0.000342768 |
| *M5005_Spy_0974* | 2941.329 | -0.695 | 0.278 | -2.499 | 0.025808732 |
| *M5005_Spy_0762* | 620.614 | 0.695 | 0.204 | 3.407 | 0.001818543 |
| *Gene or locus* | *Normalized count base mean* | *log2 fold change* | *Standard error* | *Wald test statistic* | *Benjamini-Hochberg adjusted P value* |
| *M5005_Spy_1744* | 43.146 | 0.694 | 0.347 | 1.998 | 0.080743292 |
| *rocA* | 284.884 | 0.694 | 0.220 | 3.147 | 0.004161429 |
| *gloA* | 162.651 | -0.694 | 0.182 | -3.804 | 0.000452734 |
| *M5005_Spy_1643* | 0.548 | -0.692 | 0.564 | -1.227 | NA |
| *comEC* | 10.815 | 0.691 | 0.483 | 1.429 | 0.225367981 |
| *M5005_Spy_1286* | 683.160 | 0.690 | 0.237 | 2.907 | 0.008504207 |
| *prsA.2* | 518.790 | -0.686 | 0.143 | -4.790 | 7.79E-06 |
| *pyrF* | 247.858 | 0.685 | 0.239 | 2.863 | 0.009604902 |
| *spxA* | 1923.279 | -0.683 | 0.177 | -3.861 | 0.000366693 |
| *M5005_Spy_0329* | 67.123 | -0.683 | 0.296 | -2.305 | 0.041173851 |
| *M5005_Spy_1072* | 2331.044 | 0.680 | 0.198 | 3.430 | 0.001684665 |
| *M5005_Spy_0931* | 66.341 | 0.679 | 0.346 | 1.960 | 0.087015726 |
| *M5005_Spy_1364* | 404.511 | -0.678 | 0.160 | -4.244 | 8.29E-05 |
| *lsp* | 51.827 | -0.676 | 0.285 | -2.367 | 0.035437249 |
| *M5005_Spy_0168* | 1.022 | 0.675 | 0.678 | 0.995 | NA |
| *M5005_Spy_0920* | 68.825 | 0.674 | 0.293 | 2.298 | 0.041881044 |
| *M5005_Spy_0972* | 7387.086 | -0.674 | 0.195 | -3.455 | 0.001546796 |
| *M5005_Spy_1393* | 196.327 | -0.673 | 0.170 | -3.955 | 0.000258477 |
| *M5005_Spy_0266* | 174.393 | 0.673 | 0.218 | 3.084 | 0.005046045 |
| *rgg* | 15.365 | -0.673 | 0.444 | -1.516 | 0.196260305 |
| *murG* | 1466.375 | 0.671 | 0.126 | 5.314 | 5.94E-07 |
| *M5005_Spy_1609* | 738.645 | 0.671 | 0.183 | 3.666 | 0.000745988 |
| *M5005_Spy_0962* | 289.301 | 0.668 | 0.217 | 3.078 | 0.005126022 |
| *scaR* | 140.809 | -0.668 | 0.214 | -3.119 | 0.004530351 |
| *aroB* | 154.348 | -0.668 | 0.180 | -3.719 | 0.000615746 |
| *citX* | 51.251 | 0.666 | 0.316 | 2.106 | 0.065316295 |
| *M5005_Spy_0016* | 0.511 | 0.663 | 0.576 | 1.151 | NA |
| *M5005_Spy_0535* | 8.860 | -0.662 | 0.510 | -1.299 | 0.275227109 |
| *M5005_Spy_1287* | 301.986 | 0.661 | 0.195 | 3.399 | 0.001866116 |
| *M5005_Spy_1335* | 2745.116 | 0.661 | 0.226 | 2.929 | 0.007996246 |
| *M5005_Spy_0302* | 290.388 | -0.661 | 0.185 | -3.571 | 0.00104264 |
| *M5005_Spy_1860* | 170.801 | -0.661 | 0.285 | -2.318 | 0.039983684 |
| *M5005_Spy_1579* | 356.636 | -0.659 | 0.230 | -2.866 | 0.009520826 |
| *fhuC* | 135.583 | 0.656 | 0.207 | 3.166 | 0.003919442 |
| *hflX* | 336.774 | 0.656 | 0.148 | 4.422 | 3.95E-05 |
| *M5005_Spy_0432* | 8.846 | 0.655 | 0.543 | 1.207 | 0.316838986 |
| *lytR* | 1131.122 | 0.654 | 0.223 | 2.936 | 0.00784366 |
| *M5005_Spy_1690* | 63.977 | -0.654 | 0.325 | -2.008 | 0.079100949 |
| *M5005_Spy_0305* | 103.425 | -0.652 | 0.309 | -2.114 | 0.064244585 |
| *M5005_Spy_0210* | 2.331 | -0.652 | 0.695 | -0.938 | 0.452681243 |
| *M5005_Spy_0242* | 341.713 | -0.651 | 0.313 | -2.081 | 0.068525463 |
| *sagB* | 193.611 | -0.650 | 0.354 | -1.838 | 0.10999419 |
| *hasC.2* | 458.715 | -0.650 | 0.157 | -4.150 | 0.000122667 |
| *Gene or locus* | *Normalized count base mean* | *log2 fold change* | *Standard error* | *Wald test statistic* | *Benjamini-Hochberg adjusted P value* |
| *ksgA* | 115.772 | -0.650 | 0.212 | -3.059 | 0.005440731 |
| *M5005_Spy_0963* | 352.418 | -0.648 | 0.328 | -1.979 | 0.083656666 |
| *aspS* | 592.047 | -0.646 | 0.162 | -3.981 | 0.000233755 |
| *divIB* | 1251.013 | 0.645 | 0.156 | 4.144 | 0.000125613 |
| *xseB* | 92.993 | 0.645 | 0.238 | 2.706 | 0.01486089 |
| *M5005_Spy_0572* | 439.765 | -0.645 | 0.305 | -2.111 | 0.064714992 |
| *pheT* | 394.201 | -0.643 | 0.210 | -3.058 | 0.005459947 |
| *truB* | 545.718 | 0.642 | 0.176 | 3.641 | 0.000811584 |
| *M5005_Spy_0964* | 285.758 | -0.642 | 0.399 | -1.608 | 0.167699571 |
| *M5005_Spy_1099* | 601.127 | 0.642 | 0.160 | 4.024 | 0.000201047 |
| *M5005_Spy_0654* | 86.836 | -0.639 | 0.235 | -2.717 | 0.014462267 |
| *M5005_Spy_1546* | 842.434 | -0.639 | 0.197 | -3.248 | 0.003040836 |
| *metK2* | 252.373 | -0.638 | 0.169 | -3.787 | 0.00048203 |
| *trmB* | 273.035 | -0.638 | 0.200 | -3.199 | 0.003538316 |
| *tatD* | 74.151 | -0.638 | 0.272 | -2.344 | 0.037536217 |
| *hutU* | 3.524 | -0.637 | 0.687 | -0.927 | 0.458423497 |
| *M5005_Spy_1301* | 169.993 | -0.637 | 0.237 | -2.684 | 0.015721651 |
| *M5005_Spy_0166* | 0.476 | 0.636 | 0.571 | 1.113 | NA |
| *drrA* | 62.134 | -0.635 | 0.258 | -2.464 | 0.028139084 |
| *M5005_Spy_0923* | 110.745 | -0.635 | 0.242 | -2.625 | 0.018534192 |
| *hyl* | 120.196 | 0.634 | 0.331 | 1.915 | 0.094692164 |
| *M5005_Spy_1821* | 44.937 | 0.634 | 0.284 | 2.231 | 0.048987813 |
| *M5005_Spy_0122* | 314.487 | -0.633 | 0.149 | -4.252 | 8.03E-05 |
| *M5005_Spy_0140* | 2227.943 | 0.631 | 0.222 | 2.845 | 0.010092324 |
| *M5005_Spy_0465* | 187.238 | 0.630 | 0.213 | 2.955 | 0.007418444 |
| *M5005_Spy_0477* | 86.403 | 0.630 | 0.245 | 2.575 | 0.021209806 |
| *M5005_Spy_0732* | 995.240 | 0.629 | 0.171 | 3.685 | 0.000697375 |
| *M5005_Spy_0291* | 234.221 | -0.627 | 0.188 | -3.337 | 0.002299842 |
| *M5005_Spy_1663* | 6.393 | 0.626 | 0.567 | 1.106 | 0.362921569 |
| *mur1.1* | 56.900 | 0.626 | 0.278 | 2.253 | 0.046560206 |
| *groES* | 1204.242 | -0.626 | 0.191 | -3.272 | 0.002827821 |
| *ska* | 923.939 | 0.626 | 0.290 | 2.155 | 0.058757357 |
| *M5005_Spy_0373* | 44.594 | 0.625 | 0.300 | 2.085 | 0.067911325 |
| *lemA* | 675.651 | -0.625 | 0.162 | -3.865 | 0.000361659 |
| *lacR.1* | 155.588 | -0.624 | 0.326 | -1.911 | 0.095275776 |
| *ptsB* | 775.196 | 0.623 | 0.184 | 3.389 | 0.00193304 |
| *M5005_Spy_0501* | 79.849 | -0.621 | 0.286 | -2.175 | 0.056086954 |
| *nusG* | 101.415 | -0.619 | 0.229 | -2.699 | 0.015100382 |
| *M5005_Spy_0731* | 63.066 | -0.618 | 0.272 | -2.269 | 0.044973583 |
| *M5005_Spy_1562* | 67.184 | -0.617 | 0.240 | -2.568 | 0.021568035 |
| *metB* | 19.178 | -0.613 | 0.387 | -1.587 | 0.173995706 |
| *msrA.2* | 326.951 | 0.613 | 0.231 | 2.651 | 0.017284527 |
| *M5005_Spy_0176* | 8.086 | -0.610 | 0.613 | -0.996 | 0.419652348 |
| *Gene or locus* | *Normalized count base mean* | *log2 fold change* | *Standard error* | *Wald test statistic* | *Benjamini-Hochberg adjusted P value* |
| *comEB* | 158.154 | -0.609 | 0.179 | -3.394 | 0.001895713 |
| *srtT* | 31.405 | 0.609 | 0.448 | 1.360 | 0.251263911 |
| *M5005_Spy_1104* | 396.565 | -0.608 | 0.295 | -2.060 | 0.071677052 |
| *M5005_Spy_1541* | 16.871 | 0.608 | 0.476 | 1.277 | 0.284686102 |
| *M5005_Spy_0756* | 4.280 | 0.607 | 0.630 | 0.963 | 0.438058115 |
| *murZ* | 326.592 | 0.607 | 0.157 | 3.866 | 0.000361429 |
| *M5005_Spy_0335* | 278.809 | -0.606 | 0.206 | -2.940 | 0.007752272 |
| *ntpI* | 121.467 | 0.605 | 0.305 | 1.982 | 0.083115261 |
| *M5005_Spy_0681* | 775.424 | -0.604 | 0.183 | -3.297 | 0.002603833 |
| *purR* | 502.540 | -0.604 | 0.158 | -3.820 | 0.000425391 |
| *atpC* | 1019.557 | 0.603 | 0.243 | 2.484 | 0.026828361 |
| *M5005_Spy_1256* | 72.301 | -0.603 | 0.244 | -2.467 | 0.027962085 |
| *M5005_Spy_1495* | 1421.785 | 0.602 | 0.179 | 3.366 | 0.002094486 |
| *rofA* | 943.595 | -0.602 | 0.198 | -3.032 | 0.005931054 |
| *M5005_Spy_1276* | 135.295 | 0.601 | 0.295 | 2.036 | 0.07501507 |
| *apt* | 503.849 | 0.601 | 0.139 | 4.306 | 6.47E-05 |
| *M5005_Spy_0195* | 39.458 | 0.600 | 0.351 | 1.709 | 0.140975005 |
| *sipC* | 366.524 | 0.600 | 0.212 | 2.833 | 0.010470862 |
| *M5005_Spy_0514* | 193.091 | -0.599 | 0.209 | -2.862 | 0.009604902 |
| *parB* | 1037.750 | -0.598 | 0.307 | -1.950 | 0.088718082 |
| *M5005_Spy_0371* | 609.970 | -0.597 | 0.162 | -3.681 | 0.000704609 |
| *M5005_Spy_0877* | 522.809 | -0.595 | 0.379 | -1.571 | 0.179121025 |
| *M5005_Spy_1297* | 149.670 | -0.591 | 0.331 | -1.788 | 0.121129297 |
| *valS* | 448.942 | -0.589 | 0.162 | -3.649 | 0.000792273 |
| *M5005_Spy_0332* | 70.766 | -0.589 | 0.239 | -2.461 | 0.028332507 |
| *M5005_Spy_1754* | 214.534 | -0.588 | 0.186 | -3.161 | 0.003982329 |
| *inlA* | 44.984 | 0.588 | 0.289 | 2.032 | 0.075511447 |
| *mnmA* | 1557.961 | -0.587 | 0.220 | -2.662 | 0.016766199 |
| *fhs.1* | 390.481 | 0.586 | 0.189 | 3.094 | 0.004902421 |
| *phaB* | 1071.987 | -0.584 | 0.152 | -3.849 | 0.000382637 |
| *M5005_Spy_0745* | 372.880 | 0.581 | 0.145 | 4.018 | 0.000205052 |
| *nrdR* | 89.290 | -0.581 | 0.340 | -1.706 | 0.141432244 |
| *oppC* | 710.239 | 0.580 | 0.202 | 2.876 | 0.009283948 |
| *M5005_Spy_0525* | 24.214 | -0.580 | 0.422 | -1.374 | 0.246315687 |
| *fhuB* | 26.013 | 0.577 | 0.374 | 1.545 | 0.187300029 |
| *copZ* | 29.383 | 0.577 | 0.360 | 1.601 | 0.169590089 |
| *rpoB* | 2483.894 | 0.577 | 0.182 | 3.176 | 0.003797222 |
| *M5005_Spy_1401* | 306.462 | -0.576 | 0.384 | -1.501 | 0.201110005 |
| *rpsG* | 769.300 | -0.575 | 0.208 | -2.762 | 0.012772686 |
| *M5005_Spy_0363* | 542.485 | -0.575 | 0.184 | -3.125 | 0.004451364 |
| *M5005_Spy_0473* | 36.276 | 0.573 | 0.332 | 1.723 | 0.137087379 |
| *M5005_Spy_0968* | 24.146 | -0.572 | 0.463 | -1.235 | 0.303843904 |
| *ftsA* | 3197.015 | -0.572 | 0.231 | -2.478 | 0.027250782 |
| *Gene or locus* | *Normalized count base mean* | *log2 fold change* | *Standard error* | *Wald test statistic* | *Benjamini-Hochberg adjusted P value* |
| *M5005_Spy_1093* | 56.206 | -0.572 | 0.376 | -1.520 | 0.195292083 |
| *M5005_Spy_0918* | 123.775 | 0.571 | 0.241 | 2.370 | 0.035258623 |
| *glcK* | 235.031 | -0.570 | 0.166 | -3.433 | 0.001669768 |
| *glr* | 212.893 | -0.570 | 0.335 | -1.705 | 0.141727085 |
| *M5005_Spy_0221* | 318.541 | -0.569 | 0.205 | -2.773 | 0.012375305 |
| *M5005_Spy_1470* | 327.904 | -0.568 | 0.194 | -2.924 | 0.008096215 |
| *tdk2* | 141.181 | -0.567 | 0.207 | -2.734 | 0.013778551 |
| *M5005_Spy_1403* | 0.733 | -0.566 | 0.633 | -0.895 | NA |
| *M5005_Spy_1811* | 985.775 | 0.566 | 0.169 | 3.341 | 0.002270903 |
| *pflD* | 132.495 | -0.565 | 0.296 | -1.907 | 0.095968717 |
| *M5005_Spy_1850* | 356.924 | 0.565 | 0.193 | 2.933 | 0.007891724 |
| *M5005_Spy_0261* | 1187.718 | 0.565 | 0.186 | 3.030 | 0.005963625 |
| *uvrC* | 1021.549 | 0.565 | 0.190 | 2.968 | 0.007159891 |
| *mutS* | 385.187 | 0.563 | 0.206 | 2.737 | 0.013706815 |
| *ptsC* | 628.076 | 0.562 | 0.263 | 2.138 | 0.061026003 |
| *proB* | 2222.339 | 0.561 | 0.140 | 3.999 | 0.000219712 |
| *cpsX* | 45.178 | -0.560 | 0.357 | -1.567 | 0.180158313 |
| *M5005_Spy_1678* | 113.212 | -0.559 | 0.217 | -2.573 | 0.02125644 |
| *rgpFc* | 1342.239 | 0.556 | 0.166 | 3.348 | 0.002212271 |
| *M5005_Spy_0904* | 15.919 | 0.556 | 0.436 | 1.276 | 0.284969381 |
| *atpF* | 721.069 | -0.555 | 0.206 | -2.701 | 0.015061556 |
| *M5005_Spy_1784* | 692.605 | -0.553 | 0.374 | -1.478 | 0.208148109 |
| *potC* | 222.892 | 0.553 | 0.170 | 3.250 | 0.003022077 |
| *rbgA* | 126.465 | -0.551 | 0.232 | -2.373 | 0.035088384 |
| *prsA* | 5907.352 | -0.548 | 0.152 | -3.616 | 0.000885029 |
| *M5005_Spy_0403* | 21.015 | 0.547 | 0.396 | 1.381 | 0.243920063 |
| *M5005_Spy_1601* | 1148.895 | -0.547 | 0.169 | -3.243 | 0.003073575 |
| *rgpAc* | 347.387 | -0.547 | 0.272 | -2.011 | 0.078822589 |
| *recR* | 97.068 | -0.547 | 0.265 | -2.064 | 0.071167314 |
| *srtR* | 171.376 | -0.546 | 0.208 | -2.631 | 0.018251102 |
| *agaV* | 12.008 | 0.546 | 0.557 | 0.981 | 0.427662423 |
| *M5005_Spy_0333* | 35.277 | -0.546 | 0.321 | -1.699 | 0.142988132 |
| *M5005_Spy_1071* | 1522.053 | 0.546 | 0.128 | 4.263 | 7.68E-05 |
| *M5005_Spy_0264* | 318.383 | -0.544 | 0.238 | -2.289 | 0.042853002 |
| *M5005_Spy_1281* | 36.400 | -0.544 | 0.353 | -1.542 | 0.18790776 |
| *dppB* | 125.931 | 0.543 | 0.240 | 2.260 | 0.045733755 |
| *fhuA* | 17.020 | -0.542 | 0.476 | -1.140 | 0.346292018 |
| *birA* | 104.281 | -0.542 | 0.214 | -2.530 | 0.023764491 |
| *M5005_Spy_0294* | 1.053 | -0.541 | 0.679 | -0.796 | NA |
| *glnQ.2* | 180.681 | 0.540 | 0.263 | 2.052 | 0.072844192 |
| *M5005_Spy_0690* | 113.460 | -0.540 | 0.300 | -1.796 | 0.119360668 |
| *M5005_Spy_0100* | 237.558 | 0.540 | 0.287 | 1.878 | 0.101727292 |
| *asnA* | 12.334 | -0.539 | 0.555 | -0.972 | 0.432689097 |
| *Gene or locus* | *Normalized count base mean* | *log2 fold change* | *Standard error* | *Wald test statistic* | *Benjamini-Hochberg adjusted P value* |
| *ahrC.2* | 56.075 | -0.539 | 0.272 | -1.986 | 0.082567124 |
| *M5005_Spy_0601* | 258.303 | -0.539 | 0.179 | -3.011 | 0.006293881 |
| *M5005_Spy_0470* | 184.057 | -0.537 | 0.221 | -2.425 | 0.031086029 |
| *M5005_Spy_1837* | 1244.716 | -0.535 | 0.191 | -2.800 | 0.01147728 |
| *nox* | 831.883 | -0.534 | 0.243 | -2.197 | 0.053133932 |
| *gatB* | 544.865 | 0.532 | 0.201 | 2.645 | 0.017518369 |
| *M5005_Spy_0443* | 68.580 | 0.532 | 0.263 | 2.021 | 0.076916647 |
| *M5005_Spy_1347* | 41.765 | 0.530 | 0.287 | 1.847 | 0.10819661 |
| *purN* | 12.515 | -0.527 | 0.504 | -1.046 | 0.393067566 |
| *M5005_Spy_1524* | 357.749 | 0.526 | 0.172 | 3.059 | 0.005440731 |
| *M5005_Spy_0394* | 328.002 | -0.524 | 0.207 | -2.533 | 0.023655928 |
| *nadE* | 1072.985 | -0.523 | 0.180 | -2.898 | 0.008748859 |
| *M5005_Spy_0193* | 231.239 | 0.522 | 0.248 | 2.105 | 0.065405736 |
| *pepO* | 1897.366 | -0.522 | 0.157 | -3.325 | 0.00237834 |
| *dnaG* | 723.381 | -0.521 | 0.232 | -2.251 | 0.046661612 |
| *ruvA* | 152.418 | 0.521 | 0.243 | 2.146 | 0.059884926 |
| *M5005_Spy_0169* | 21.463 | 0.520 | 0.417 | 1.245 | 0.299604213 |
| *apbA* | 779.742 | -0.520 | 0.219 | -2.371 | 0.035231793 |
| *sagE* | 104.481 | -0.519 | 0.399 | -1.301 | 0.274570823 |
| *M5005_Spy_1288* | 467.898 | 0.519 | 0.190 | 2.735 | 0.013760367 |
| *M5005_Spy_0462* | 951.633 | 0.518 | 0.139 | 3.739 | 0.000575315 |
| *M5005_Spy_0679* | 344.166 | -0.518 | 0.161 | -3.212 | 0.003393929 |
| *smeZ* | 77.671 | -0.517 | 0.292 | -1.774 | 0.124680067 |
| *hsdM* | 166.518 | -0.517 | 0.214 | -2.421 | 0.031428691 |
| *lacZ* | 102.585 | 0.517 | 0.264 | 1.955 | 0.087923225 |
| *M5005_Spy_1564* | 172.867 | -0.514 | 0.268 | -1.919 | 0.093735274 |
| *M5005_Spy_1343* | 65.175 | -0.513 | 0.312 | -1.648 | 0.156498121 |
| *M5005_Spy_0259* | 2.286 | 0.510 | 0.698 | 0.731 | 0.570569799 |
| *M5005_Spy_1399* | 1130.991 | 0.510 | 0.164 | 3.098 | 0.004836742 |
| *M5005_Spy_1848* | 953.509 | -0.510 | 0.219 | -2.324 | 0.039459092 |
| *M5005_Spy_0080* | 13.062 | -0.509 | 0.446 | -1.143 | 0.344791312 |
| *dacA1* | 276.036 | 0.509 | 0.164 | 3.108 | 0.004702829 |
| *vlg* | 584.860 | 0.509 | 0.132 | 3.841 | 0.000393625 |
| *M5005_Spy_0181* | 1.329 | 0.509 | 0.703 | 0.724 | NA |
| *M5005_Spy_1593* | 511.608 | -0.509 | 0.292 | -1.743 | 0.131980634 |
| *atoE* | 5.526 | 0.508 | 0.589 | 0.861 | 0.499101228 |
| *M5005_Spy_0267* | 191.896 | -0.507 | 0.194 | -2.614 | 0.019080646 |
| *M5005_Spy_1517* | 67.970 | -0.507 | 0.238 | -2.130 | 0.06212771 |
| *M5005_Spy_0622* | 911.067 | 0.506 | 0.209 | 2.424 | 0.031133896 |
| *divIVAS* | 1997.464 | 0.506 | 0.188 | 2.691 | 0.015490183 |
| *M5005_Spy_0442* | 33.538 | -0.505 | 0.402 | -1.257 | 0.293928472 |
| *atoD.2* | 3.780 | -0.505 | 0.640 | -0.788 | 0.539924975 |
| *adh2* | 1006.717 | 0.503 | 0.175 | 2.882 | 0.009144778 |
| *Gene or locus* | *Normalized count base mean* | *log2 fold change* | *Standard error* | *Wald test statistic* | *Benjamini-Hochberg adjusted P value* |
| *M5005_Spy_0314* | 81.810 | -0.503 | 0.284 | -1.772 | 0.124889585 |
| *M5005_Spy_0559* | 12.182 | -0.503 | 0.555 | -0.906 | 0.470554068 |
| *M5005_Spy_0975* | 4450.881 | -0.503 | 0.181 | -2.778 | 0.012221332 |
| *M5005_Spy_1131* | 102.467 | 0.503 | 0.245 | 2.050 | 0.073057442 |
| *M5005_Spy_0416* | 68.780 | 0.502 | 0.269 | 1.865 | 0.104526408 |
| *M5005_Spy_0315* | 505.807 | 0.502 | 0.199 | 2.526 | 0.023996759 |
| *scrB* | 41.808 | -0.499 | 0.316 | -1.580 | 0.176029945 |
| *M5005_Spy_1135* | 94.930 | 0.499 | 0.244 | 2.042 | 0.074125381 |
| *M5005_Spy_0177* | 21.579 | -0.498 | 0.493 | -1.009 | 0.413094801 |
| *M5005_Spy_1615* | 1050.552 | 0.497 | 0.207 | 2.398 | 0.033139981 |
| *citF* | 84.404 | 0.496 | 0.295 | 1.682 | 0.147572305 |
| *obgE* | 794.030 | 0.496 | 0.201 | 2.464 | 0.028167639 |
| *M5005_Spy_1294* | 25.177 | -0.495 | 0.350 | -1.413 | 0.231043961 |
| *M5005_Spy_0425* | 913.688 | -0.495 | 0.214 | -2.313 | 0.040438297 |
| *opuAA* | 117.470 | 0.494 | 0.327 | 1.513 | 0.197123255 |
| *M5005_Spy_0219* | 55.931 | -0.492 | 0.319 | -1.540 | 0.18830475 |
| *M5005_Spy_1790* | 660.546 | 0.492 | 0.143 | 3.437 | 0.001648035 |
| *srtG* | 25.827 | 0.491 | 0.358 | 1.371 | 0.247703814 |
| *M5005_Spy_0032* | 471.047 | -0.489 | 0.208 | -2.344 | 0.037536217 |
| *rimM* | 623.492 | 0.488 | 0.165 | 2.955 | 0.007418444 |
| *ntpA* | 100.144 | 0.488 | 0.319 | 1.529 | 0.192048918 |
| *aspC* | 632.278 | -0.488 | 0.277 | -1.759 | 0.128312488 |
| *ffh* | 1203.744 | -0.488 | 0.169 | -2.890 | 0.008946935 |
| *ylxM* | 205.182 | -0.487 | 0.269 | -1.809 | 0.1164237 |
| *smf* | 3.029 | -0.486 | 0.678 | -0.717 | 0.578668828 |
| *tilS* | 1381.630 | -0.486 | 0.224 | -2.171 | 0.056549913 |
| *pepF* | 192.579 | -0.486 | 0.236 | -2.059 | 0.071784875 |
| *M5005_Spy_0738* | 304.166 | 0.483 | 0.185 | 2.615 | 0.019015125 |
| *M5005_Spy_0617* | 762.740 | -0.480 | 0.317 | -1.516 | 0.196389176 |
| *sunL* | 414.357 | -0.479 | 0.249 | -1.924 | 0.093114752 |
| *argR1* | 289.873 | 0.478 | 0.268 | 1.787 | 0.121224185 |
| *M5005_Spy_0515* | 87.030 | -0.478 | 0.338 | -1.416 | 0.23008935 |
| *M5005_Spy_0237* | 447.304 | 0.477 | 0.148 | 3.220 | 0.003320483 |
| *M5005_Spy_1091* | 1.313 | -0.477 | 0.655 | -0.728 | NA |
| *M5005_Spy_1317* | 159.574 | -0.476 | 0.234 | -2.030 | 0.075720884 |
| *M5005_Spy_0171* | 3.464 | -0.474 | 0.651 | -0.728 | 0.57215829 |
| *cadD* | 11.899 | -0.474 | 0.574 | -0.826 | 0.517997498 |
| *tgt* | 226.032 | -0.474 | 0.173 | -2.730 | 0.013919799 |
| *M5005_Spy_0788* | 55.217 | 0.471 | 0.367 | 1.284 | 0.281316957 |
| *malG* | 80.761 | 0.471 | 0.302 | 1.560 | 0.182420245 |
| *M5005_Spy_1278* | 199.450 | -0.471 | 0.195 | -2.412 | 0.032093625 |
| *M5005_Spy_1245* | 3596.949 | 0.469 | 0.144 | 3.257 | 0.002955657 |
| *M5005_Spy_1685* | 276.747 | 0.469 | 0.217 | 2.157 | 0.058533762 |
| *Gene or locus* | *Normalized count base mean* | *log2 fold change* | *Standard error* | *Wald test statistic* | *Benjamini-Hochberg adjusted P value* |
| *M5005_Spy_1861* | 563.568 | -0.468 | 0.184 | -2.542 | 0.023109477 |
| *M5005_Spy_1659* | 152.727 | -0.467 | 0.194 | -2.411 | 0.032106982 |
| *M5005_Spy_0383* | 58.373 | -0.467 | 0.247 | -1.891 | 0.098890521 |
| *hemN* | 233.466 | -0.466 | 0.308 | -1.514 | 0.196867844 |
| *M5005_Spy_0346* | 5.536 | -0.465 | 0.634 | -0.733 | 0.56964677 |
| *dinG* | 250.266 | -0.464 | 0.241 | -1.926 | 0.092716855 |
| *comYB* | 3.910 | 0.464 | 0.646 | 0.718 | 0.578424965 |
| *norA* | 1129.027 | 0.464 | 0.219 | 2.117 | 0.064011947 |
| *M5005_Spy_0036* | 465.809 | 0.463 | 0.191 | 2.427 | 0.030964226 |
| *rplX* | 2520.353 | 0.462 | 0.243 | 1.898 | 0.097851813 |
| *dpfB* | 95.628 | -0.462 | 0.292 | -1.583 | 0.175127472 |
| *M5005_Spy_0837* | 307.264 | 0.461 | 0.156 | 2.965 | 0.007219389 |
| *pyrE* | 140.504 | 0.459 | 0.258 | 1.780 | 0.123065773 |
| *M5005_Spy_1166* | 76.723 | -0.456 | 0.268 | -1.701 | 0.142676018 |
| *ccpA* | 313.466 | -0.455 | 0.245 | -1.858 | 0.105897691 |
| *ebsA* | 392.798 | -0.455 | 0.225 | -2.021 | 0.076916647 |
| *fhuD* | 45.856 | 0.455 | 0.274 | 1.660 | 0.153466866 |
| *fabK* | 3149.546 | 0.454 | 0.222 | 2.047 | 0.073377442 |
| *M5005_Spy_0888* | 0.762 | 0.454 | 0.663 | 0.686 | NA |
| *nadD* | 512.706 | 0.454 | 0.202 | 2.251 | 0.046686385 |
| *M5005_Spy_0012* | 1985.042 | -0.454 | 0.276 | -1.644 | 0.156963785 |
| *hylA* | 310.063 | -0.454 | 0.219 | -2.072 | 0.069860624 |
| *thiD* | 353.416 | -0.452 | 0.251 | -1.805 | 0.117212543 |
| *M5005_Spy_0814* | 51.865 | -0.451 | 0.275 | -1.644 | 0.156963785 |
| *M5005_Spy_1523* | 261.926 | 0.450 | 0.197 | 2.278 | 0.043924531 |
| *M5005_Spy_0299* | 101.141 | -0.450 | 0.283 | -1.591 | 0.172609525 |
| *fhuG* | 43.153 | 0.449 | 0.316 | 1.420 | 0.228370675 |
| *sagC* | 282.905 | -0.447 | 0.312 | -1.435 | 0.223598288 |
| *M5005_Spy_1521* | 502.370 | 0.444 | 0.186 | 2.386 | 0.033991505 |
| *M5005_Spy_1826* | 49.829 | 0.444 | 0.324 | 1.369 | 0.248406059 |
| *M5005_Spy_0629* | 144.710 | 0.444 | 0.248 | 1.789 | 0.121033425 |
| *M5005_Spy_0912* | 1.708 | 0.443 | 0.707 | 0.627 | NA |
| *covS* | 578.504 | -0.443 | 0.211 | -2.105 | 0.065405736 |
| *M5005_Spy_0886* | 2.533 | 0.443 | 0.684 | 0.647 | 0.615231657 |
| *ddl* | 306.763 | -0.443 | 0.218 | -2.031 | 0.075559135 |
| *M5005_Spy_0880* | 152.229 | 0.442 | 0.222 | 1.997 | 0.080792982 |
| *M5005_Spy_0397* | 13.712 | -0.442 | 0.472 | -0.936 | 0.453736891 |
| *dnaE* | 934.989 | 0.441 | 0.134 | 3.298 | 0.002596887 |
| *dnaB* | 268.460 | -0.441 | 0.227 | -1.939 | 0.090505622 |
| *M5005_Spy_0137* | 1173.161 | -0.440 | 0.146 | -3.014 | 0.006245696 |
| *M5005_Spy_0550* | 75.712 | 0.439 | 0.242 | 1.811 | 0.116022304 |
| *M5005_Spy_0852* | 257.239 | 0.439 | 0.209 | 2.097 | 0.066316888 |
| *pepT* | 1280.613 | -0.437 | 0.165 | -2.647 | 0.017485655 |
| *Gene or locus* | *Normalized count base mean* | *log2 fold change* | *Standard error* | *Wald test statistic* | *Benjamini-Hochberg adjusted P value* |
| *M5005_Spy_0471* | 664.789 | 0.437 | 0.134 | 3.254 | 0.002983306 |
| *oadA1* | 124.498 | 0.436 | 0.218 | 1.999 | 0.080652797 |
| *M5005_Spy_0095* | 3.717 | -0.435 | 0.668 | -0.651 | 0.613643151 |
| *M5005_Spy_1758* | 228.140 | -0.435 | 0.184 | -2.367 | 0.035447883 |
| *M5005_Spy_1271* | 2101.490 | -0.432 | 0.248 | -1.739 | 0.132946185 |
| *M5005_Spy_1691* | 37.512 | 0.431 | 0.469 | 0.920 | 0.463255063 |
| *M5005_Spy_1394* | 284.816 | -0.430 | 0.159 | -2.712 | 0.01465332 |
| *coaE* | 221.548 | 0.430 | 0.180 | 2.385 | 0.034066355 |
| *folC.2* | 311.979 | 0.427 | 0.165 | 2.592 | 0.020212488 |
| *M5005_Spy_1779* | 106.276 | 0.425 | 0.339 | 1.255 | 0.294625082 |
| *M5005_Spy_0234* | 0.843 | -0.424 | 0.686 | -0.618 | NA |
| *M5005_Spy_0969* | 0.345 | 0.423 | 0.492 | 0.860 | NA |
| *acoL* | 945.999 | -0.423 | 0.280 | -1.508 | 0.19848335 |
| *M5005_Spy_0392* | 1.864 | -0.422 | 0.703 | -0.601 | NA |
| *M5005_Spy_0853* | 45.643 | -0.422 | 0.440 | -0.958 | 0.441067255 |
| *M5005_Spy_0240* | 472.534 | 0.421 | 0.143 | 2.940 | 0.007752272 |
| *fasB* | 128.267 | -0.421 | 0.319 | -1.317 | 0.268114829 |
| *M5005_Spy_1721* | 1.819 | -0.420 | 0.708 | -0.594 | NA |
| *M5005_Spy_0707* | 442.255 | -0.418 | 0.231 | -1.812 | 0.116022304 |
| *M5005_Spy_1667* | 2.917 | -0.418 | 0.669 | -0.624 | 0.627954496 |
| *lppC* | 2442.088 | -0.418 | 0.186 | -2.242 | 0.047675284 |
| *M5005_Spy_1264* | 105.338 | -0.413 | 0.299 | -1.381 | 0.243920063 |
| *M5005_Spy_1598* | 73.285 | -0.413 | 0.275 | -1.500 | 0.201382457 |
| *M5005_Spy_1376* | 103.062 | 0.413 | 0.506 | 0.817 | 0.522909133 |
| *radA* | 136.569 | -0.413 | 0.228 | -1.812 | 0.116022304 |
| *fasA* | 203.551 | 0.412 | 0.196 | 2.102 | 0.065681707 |
| *murA* | 723.011 | -0.412 | 0.212 | -1.942 | 0.089947842 |
| *pcp* | 141.417 | -0.411 | 0.244 | -1.682 | 0.147572305 |
| *M5005_Spy_0591* | 119.203 | -0.407 | 0.208 | -1.951 | 0.088549232 |
| *M5005_Spy_0401* | 0.297 | 0.406 | 0.485 | 0.837 | NA |
| *M5005_Spy_0510* | 251.535 | -0.402 | 0.166 | -2.417 | 0.031655806 |
| *M5005_Spy_0750* | 379.792 | -0.402 | 0.164 | -2.449 | 0.029263568 |
| *M5005_Spy_0119* | 7.020 | 0.399 | 0.557 | 0.716 | 0.578897933 |
| *tag* | 84.029 | 0.398 | 0.240 | 1.656 | 0.154340385 |
| *M5005_Spy_1570* | 426.443 | -0.396 | 0.182 | -2.176 | 0.055967961 |
| *M5005_Spy_1272* | 3579.011 | 0.395 | 0.213 | 1.860 | 0.105459848 |
| *fps* | 414.100 | 0.395 | 0.246 | 1.611 | 0.167104153 |
| *ihk* | 589.054 | 0.395 | 0.160 | 2.468 | 0.027959188 |
| *M5005_Spy_1649* | 98.773 | -0.394 | 0.331 | -1.189 | 0.323389832 |
| *M5005_Spy_0454* | 0.256 | 0.393 | 0.480 | 0.819 | NA |
| *M5005_Spy_0686* | 188.195 | -0.393 | 0.170 | -2.317 | 0.039983684 |
| *accB* | 694.371 | -0.392 | 0.326 | -1.203 | 0.318353314 |
| *M5005_Spy_0201* | 74.370 | -0.391 | 0.244 | -1.607 | 0.167949631 |
| *Gene or locus* | *Normalized count base mean* | *log2 fold change* | *Standard error* | *Wald test statistic* | *Benjamini-Hochberg adjusted P value* |
| *bcaT* | 435.301 | -0.391 | 0.182 | -2.150 | 0.059385413 |
| *atpA* | 2071.071 | -0.391 | 0.242 | -1.614 | 0.166349308 |
| *pacL* | 1313.023 | 0.391 | 0.189 | 2.064 | NA |
| *acoB* | 611.935 | -0.390 | 0.208 | -1.871 | 0.103310173 |
| *M5005_Spy_1572* | 75.599 | 0.389 | 0.257 | 1.509 | 0.198373544 |
| *M5005_Spy_1595* | 1043.075 | -0.388 | 0.139 | -2.795 | 0.011634755 |
| *M5005_Spy_0841* | 141.437 | -0.388 | 0.221 | -1.756 | 0.128926522 |
| *trpG* | 59.686 | -0.387 | 0.334 | -1.160 | 0.336946486 |
| *M5005_Spy_0209* | 367.506 | -0.386 | 0.212 | -1.817 | 0.11497709 |
| *M5005_Spy_1161* | 274.117 | -0.386 | 0.199 | -1.941 | 0.090254371 |
| *M5005_Spy_0571* | 2217.364 | 0.385 | 0.357 | 1.080 | 0.377189977 |
| *M5005_Spy_1745* | 6.502 | 0.384 | 0.565 | 0.680 | 0.597257805 |
| *M5005_Spy_1280* | 270.804 | 0.383 | 0.196 | 1.949 | 0.088726758 |
| *M5005_Spy_0334* | 278.127 | -0.381 | 0.205 | -1.861 | 0.105338858 |
| *M5005_Spy_0658* | 805.634 | -0.381 | 0.183 | -2.078 | 0.068927671 |
| *M5005_Spy_0520* | 22.873 | -0.379 | 0.540 | -0.702 | 0.585365795 |
| *speB* | 512.085 | -0.379 | 0.294 | -1.288 | 0.279943248 |
| *M5005_Spy_1791* | 831.138 | -0.378 | 0.150 | -2.516 | 0.024652672 |
| *M5005_Spy_1661* | 6.629 | 0.377 | 0.595 | 0.634 | 0.622927817 |
| *mutR* | 238.188 | 0.377 | 0.242 | 1.558 | 0.182813147 |
| *M5005_Spy_1518* | 134.560 | 0.377 | 0.225 | 1.676 | 0.149132228 |
| *nth* | 164.065 | 0.376 | 0.206 | 1.825 | 0.11310992 |
| *M5005_Spy_0387* | 114.123 | -0.376 | 0.313 | -1.200 | 0.319161545 |
| *M5005_Spy_1226* | 303.790 | 0.376 | 0.183 | 2.051 | 0.072910656 |
| *M5005_Spy_0482* | 28.741 | 0.375 | 0.351 | 1.068 | 0.381729306 |
| *rnc* | 259.003 | 0.373 | 0.249 | 1.498 | 0.202188817 |
| *M5005_Spy_0558* | 4.633 | -0.373 | 0.605 | -0.616 | 0.633220562 |
| *mutT* | 165.711 | -0.372 | 0.261 | -1.422 | 0.227985857 |
| *M5005_Spy_0889* | 1.935 | -0.372 | 0.704 | -0.528 | NA |
| *M5005_Spy_0754* | 2.135 | -0.371 | 0.708 | -0.524 | NA |
| *oppA* | 2972.449 | -0.370 | 0.139 | -2.675 | 0.016160227 |
| *M5005_Spy_1660* | 655.930 | -0.370 | 0.220 | -1.679 | 0.148168821 |
| *M5005_Spy_0208* | 622.547 | -0.370 | 0.187 | -1.977 | 0.083906554 |
| *M5005_Spy_0429* | 33.602 | 0.369 | 0.312 | 1.184 | 0.326055838 |
| *M5005_Spy_1775* | 1.945 | -0.369 | 0.705 | -0.523 | NA |
| *M5005_Spy_0125* | 12.897 | 0.368 | 0.469 | 0.785 | 0.541591086 |
| *fruB* | 3350.390 | 0.366 | 0.225 | 1.627 | 0.16233863 |
| *folQ* | 468.566 | 0.366 | 0.187 | 1.953 | 0.088149805 |
| *M5005_Spy_1854* | 104.382 | -0.364 | 0.245 | -1.485 | 0.206402712 |
| *uvrA* | 1447.615 | 0.363 | 0.152 | 2.397 | 0.03318304 |
| *M5005_Spy_0173* | 216.334 | 0.363 | 0.192 | 1.892 | 0.09879637 |
| *mvaD* | 182.387 | 0.361 | 0.178 | 2.025 | 0.076538038 |
| *M5005_Spy_0089* | 0.694 | 0.360 | 0.620 | 0.580 | NA |
| *Gene or locus* | *Normalized count base mean* | *log2 fold change* | *Standard error* | *Wald test statistic* | *Benjamini-Hochberg adjusted P value* |
| *mtsA* | 5561.212 | -0.360 | 0.207 | -1.737 | 0.133318156 |
| *mutL* | 839.105 | 0.359 | 0.179 | 2.008 | 0.079100949 |
| *cmk* | 326.413 | -0.359 | 0.170 | -2.116 | 0.064057127 |
| *hasB* | 3454.981 | 0.358 | 0.213 | 1.679 | 0.148168821 |
| *M5005_Spy_1670* | 115.654 | -0.358 | 0.215 | -1.665 | 0.151925256 |
| *atpD* | 1345.190 | -0.357 | 0.171 | -2.089 | 0.067478846 |
| *M5005_Spy_1530* | 1591.733 | 0.354 | 0.140 | 2.526 | 0.023996759 |
| *kgdA* | 15.267 | 0.351 | 0.433 | 0.811 | 0.526285513 |
| *malP* | 24.299 | -0.351 | 0.512 | -0.686 | 0.59420971 |
| *cpsFQ* | 771.339 | -0.351 | 0.165 | -2.129 | 0.062281298 |
| *M5005_Spy_0982* | 365.563 | -0.350 | 0.206 | -1.693 | 0.144711411 |
| *rplI* | 289.469 | -0.349 | 0.182 | -1.922 | 0.09325498 |
| *xerD* | 135.004 | 0.349 | 0.240 | 1.453 | 0.217077272 |
| *M5005_Spy_1322* | 1.208 | 0.348 | 0.649 | 0.536 | NA |
| *recA* | 517.370 | -0.346 | 0.170 | -2.039 | 0.074482458 |
| *M5005_Spy_0749* | 73.773 | 0.346 | 0.331 | 1.046 | 0.393067566 |
| *carB* | 1431.789 | 0.346 | 0.264 | 1.310 | 0.270458632 |
| *M5005_Spy_1142* | 6.810 | -0.346 | 0.544 | -0.635 | 0.622829924 |
| *cysM* | 372.211 | -0.345 | 0.151 | -2.291 | 0.042693171 |
| *M5005_Spy_0849* | 125.910 | -0.344 | 0.211 | -1.633 | 0.1603617 |
| *M5005_Spy_0010* | 650.010 | 0.344 | 0.163 | 2.116 | 0.064057127 |
| *dppA* | 2623.861 | 0.344 | 0.236 | 1.454 | 0.217077272 |
| *isp2* | 2068.094 | 0.343 | 0.219 | 1.568 | 0.179730409 |
| *cbf* | 230.003 | -0.343 | 0.232 | -1.477 | 0.208486762 |
| *M5005_Spy_1574* | 71.874 | 0.343 | 0.306 | 1.120 | 0.356009772 |
| *braB* | 77.156 | 0.342 | 0.316 | 1.082 | 0.376514502 |
| *M5005_Spy_1728* | 577.621 | 0.341 | 0.166 | 2.053 | 0.072743854 |
| *M5005_Spy_1812* | 697.918 | 0.340 | 0.176 | 1.936 | 0.090923561 |
| *ciaH* | 809.991 | 0.340 | 0.250 | 1.359 | 0.251676558 |
| *M5005_Spy_0092* | 2.624 | -0.339 | 0.679 | -0.500 | 0.709559611 |
| *yaaA* | 111.357 | 0.338 | 0.256 | 1.323 | 0.26588675 |
| *M5005_Spy_0404* | 0.508 | -0.338 | 0.589 | -0.574 | NA |
| *M5005_Spy_1740* | 221.676 | -0.338 | 0.182 | -1.860 | 0.105459848 |
| *M5005_Spy_0769* | 305.969 | 0.337 | 0.194 | 1.739 | 0.132956391 |
| *cysE* | 398.762 | 0.337 | 0.203 | 1.658 | 0.15380217 |
| *M5005_Spy_1750* | 7.609 | 0.336 | 0.551 | 0.611 | 0.636770106 |
| *M5005_Spy_0301* | 529.484 | -0.335 | 0.243 | -1.380 | 0.243920063 |
| *irr* | 514.202 | 0.335 | 0.164 | 2.046 | 0.073423509 |
| *arcB* | 1460.628 | -0.335 | 0.195 | -1.717 | 0.138461791 |
| *M5005_Spy_0225* | 177.987 | -0.334 | 0.320 | -1.046 | 0.393116234 |
| *M5005_Spy_0973* | 16550.742 | -0.332 | 0.193 | -1.721 | 0.137508809 |
| *M5005_Spy_1697* | 126.856 | -0.332 | 0.303 | -1.096 | 0.368240482 |
| *M5005_Spy_0489* | 65.969 | 0.332 | 0.298 | 1.114 | 0.358814569 |
| *Gene or locus* | *Normalized count base mean* | *log2 fold change* | *Standard error* | *Wald test statistic* | *Benjamini-Hochberg adjusted P value* |
| *M5005_Spy_1823* | 132.980 | -0.331 | 0.275 | -1.203 | 0.318353314 |
| *M5005_Spy_0774* | 104.924 | -0.331 | 0.254 | -1.302 | 0.274067432 |
| *smpB* | 192.280 | 0.330 | 0.218 | 1.517 | 0.196260305 |
| *murC* | 1098.803 | -0.330 | 0.198 | -1.667 | 0.151294412 |
| *M5005_Spy_1644* | 0.700 | 0.330 | 0.620 | 0.532 | NA |
| *M5005_Spy_0544* | 50.615 | -0.330 | 0.298 | -1.107 | 0.362324316 |
| *M5005_Spy_0491* | 259.210 | 0.329 | 0.163 | 2.023 | 0.076712554 |
| *M5005_Spy_0366* | 697.901 | -0.329 | 0.158 | -2.078 | 0.06887445 |
| *M5005_Spy_1476* | 327.163 | -0.328 | 0.234 | -1.401 | 0.23564901 |
| *M5005_Spy_0503* | 123.689 | -0.328 | 0.223 | -1.468 | 0.211486285 |
| *uvrB* | 887.303 | 0.328 | 0.172 | 1.908 | 0.095745673 |
| *M5005_Spy_1502* | 361.338 | 0.327 | 0.163 | 2.005 | 0.079591604 |
| *M5005_Spy_0917* | 297.462 | -0.327 | 0.274 | -1.194 | 0.32162286 |
| *apbE* | 29.202 | -0.327 | 0.394 | -0.830 | 0.515350304 |
| *M5005_Spy_1262* | 340.591 | 0.326 | 0.159 | 2.047 | 0.073377442 |
| *M5005_Spy_0260* | 550.372 | 0.326 | 0.184 | 1.771 | 0.125107038 |
| *M5005_Spy_1560* | 419.756 | 0.325 | 0.194 | 1.674 | 0.149465125 |
| *fruA* | 2394.721 | 0.325 | 0.171 | 1.897 | 0.09797098 |
| *M5005_Spy_0104* | 256.451 | -0.325 | 0.186 | -1.748 | 0.130820332 |
| *ackA* | 1195.467 | 0.325 | 0.218 | 1.487 | 0.205616277 |
| *rplF* | 731.954 | 0.324 | 0.171 | 1.893 | 0.09879637 |
| *M5005_Spy_0775* | 20.947 | -0.324 | 0.459 | -0.705 | 0.585272645 |
| *shp* | 230.618 | 0.324 | 0.203 | 1.597 | 0.17090917 |
| *gcp* | 486.706 | 0.324 | 0.201 | 1.612 | 0.166935301 |
| *queA* | 120.659 | -0.323 | 0.301 | -1.074 | 0.378591561 |
| *M5005_Spy_0461* | 96.089 | -0.321 | 0.218 | -1.474 | 0.209292226 |
| *dltA* | 1518.885 | 0.320 | 0.161 | 1.989 | 0.081975118 |
| *M5005_Spy_1505* | 36.170 | 0.320 | 0.399 | 0.801 | 0.532298403 |
| *nifS1* | 403.521 | -0.320 | 0.235 | -1.359 | 0.251676558 |
| *cdsA* | 220.092 | -0.319 | 0.182 | -1.758 | 0.128630114 |
| *M5005_Spy_0798* | 0.514 | -0.319 | 0.584 | -0.545 | NA |
| *sdhA* | 155.894 | -0.318 | 0.197 | -1.614 | 0.166349308 |
| *M5005_Spy_1328* | 194.538 | -0.318 | 0.184 | -1.722 | 0.137346209 |
| *dfp* | 359.653 | 0.317 | 0.237 | 1.341 | 0.258763255 |
| *M5005_Spy_1522* | 259.961 | 0.317 | 0.172 | 1.844 | 0.108778634 |
| *ciaR* | 1362.325 | 0.317 | 0.232 | 1.364 | 0.250097653 |
| *M5005_Spy_0512* | 226.909 | -0.317 | 0.219 | -1.449 | 0.218803966 |
| *M5005_Spy_1363* | 169.134 | -0.316 | 0.221 | -1.426 | 0.226386136 |
| *M5005_Spy_1552* | 194.501 | -0.315 | 0.260 | -1.210 | 0.315743797 |
| *atpE* | 1544.311 | -0.314 | 0.252 | -1.244 | 0.299934126 |
| *M5005_Spy_1679* | 208.283 | 0.313 | 0.217 | 1.438 | 0.222788124 |
| *M5005_Spy_0815* | 155.723 | -0.312 | 0.179 | -1.748 | 0.130820332 |
| *ftsX* | 676.849 | 0.311 | 0.254 | 1.227 | 0.307497574 |
| *Gene or locus* | *Normalized count base mean* | *log2 fold change* | *Standard error* | *Wald test statistic* | *Benjamini-Hochberg adjusted P value* |
| *M5005_Spy_1515* | 689.992 | -0.311 | 0.172 | -1.812 | 0.116022304 |
| *glyQ* | 168.114 | -0.311 | 0.250 | -1.242 | 0.300707238 |
| *spi* | 142.922 | -0.308 | 0.227 | -1.356 | 0.252783473 |
| *M5005_Spy_1620* | 88.578 | -0.307 | 0.267 | -1.153 | 0.339673756 |
| *M5005_Spy_1820* | 24.176 | 0.306 | 0.363 | 0.844 | 0.508299947 |
| *glyA* | 230.200 | 0.306 | 0.211 | 1.447 | 0.219317061 |
| *M5005_Spy_1391* | 592.311 | -0.304 | 0.251 | -1.209 | 0.315964731 |
| *pgmA* | 980.270 | -0.304 | 0.153 | -1.983 | 0.082937186 |
| *infB* | 2677.427 | -0.304 | 0.156 | -1.949 | 0.088726758 |
| *vicR* | 468.076 | -0.303 | 0.196 | -1.545 | 0.187401069 |
| *gltX* | 818.716 | -0.303 | 0.162 | -1.866 | 0.104368432 |
| *M5005_Spy_1389* | 425.872 | 0.302 | 0.163 | 1.856 | 0.106256777 |
| *potB* | 127.460 | 0.302 | 0.197 | 1.533 | 0.190746598 |
| *purH* | 17.799 | -0.302 | 0.412 | -0.733 | 0.569590715 |
| *M5005_Spy_0090* | 0.870 | -0.302 | 0.624 | -0.483 | NA |
| *M5005_Spy_1308* | 25.399 | 0.301 | 0.366 | 0.823 | 0.520231005 |
| *M5005_Spy_0292* | 1810.461 | -0.299 | 0.214 | -1.401 | 0.23564901 |
| *M5005_Spy_0787* | 26.823 | -0.299 | 0.426 | -0.703 | 0.585365795 |
| *M5005_Spy_1726* | 391.655 | 0.299 | 0.166 | 1.801 | 0.118151809 |
| *M5005_Spy_0479* | 2.098 | 0.299 | 0.704 | 0.424 | NA |
| *M5005_Spy_0414* | 383.050 | 0.297 | 0.192 | 1.546 | 0.187047094 |
| *M5005_Spy_1310* | 19.175 | 0.297 | 0.412 | 0.721 | 0.576457866 |
| *M5005_Spy_0431* | 0.201 | -0.294 | 0.406 | -0.724 | NA |
| *M5005_Spy_1386* | 750.298 | -0.293 | 0.213 | -1.379 | 0.244319066 |
| *ppnK* | 634.919 | 0.293 | 0.138 | 2.120 | 0.06358795 |
| *M5005_Spy_1510* | 1.202 | -0.292 | 0.648 | -0.451 | NA |
| *M5005_Spy_1832* | 3.664 | 0.292 | 0.640 | 0.455 | 0.733934682 |
| *M5005_Spy_0034* | 118.437 | 0.291 | 0.297 | 0.980 | 0.428340931 |
| *capA* | 604.359 | 0.291 | 0.197 | 1.480 | 0.207797929 |
| *M5005_Spy_0813* | 119.337 | -0.290 | 0.247 | -1.173 | 0.330745411 |
| *rplA* | 633.287 | 0.289 | 0.224 | 1.288 | 0.279943248 |
| *dpiA* | 8.421 | -0.286 | 0.506 | -0.566 | 0.664186678 |
| *M5005_Spy_0189* | 2.767 | 0.284 | 0.677 | 0.420 | 0.753163809 |
| *M5005_Spy_0037* | 792.937 | 0.283 | 0.162 | 1.748 | 0.130820332 |
| *M5005_Spy_0592* | 231.350 | -0.283 | 0.246 | -1.151 | 0.3404563 |
| *manL* | 462.087 | -0.283 | 0.213 | -1.325 | 0.264821342 |
| *ychF* | 1626.894 | -0.282 | 0.141 | -1.994 | 0.081205436 |
| *citG* | 48.237 | -0.281 | 0.298 | -0.941 | 0.451063685 |
| *M5005_Spy_1352* | 683.379 | -0.280 | 0.270 | -1.038 | 0.397127564 |
| *M5005_Spy_1794* | 502.892 | -0.280 | 0.174 | -1.604 | 0.168781009 |
| *M5005_Spy_1125* | 52.254 | 0.279 | 0.310 | 0.901 | 0.473503095 |
| *M5005_Spy_0540* | 862.791 | -0.279 | 0.154 | -1.811 | 0.116022304 |
| *gid* | 321.958 | 0.277 | 0.193 | 1.433 | 0.223777265 |
| *Gene or locus* | *Normalized count base mean* | *log2 fold change* | *Standard error* | *Wald test statistic* | *Benjamini-Hochberg adjusted P value* |
| *speG* | 184.121 | -0.277 | 0.231 | -1.200 | 0.319161545 |
| *malR* | 165.204 | -0.275 | 0.234 | -1.179 | 0.328020625 |
| *M5005_Spy_1247* | 2186.240 | -0.275 | 0.140 | -1.967 | 0.085615078 |
| *M5005_Spy_1086* | 20.448 | -0.274 | 0.415 | -0.660 | 0.609861382 |
| *M5005_Spy_0492* | 0.183 | -0.274 | 0.394 | -0.695 | NA |
| *comX1.1* | 0.183 | -0.274 | 0.394 | -0.695 | NA |
| *M5005_Spy_1115* | 17.454 | 0.274 | 0.419 | 0.653 | 0.61273831 |
| *prfB* | 689.507 | 0.272 | 0.189 | 1.438 | 0.222788124 |
| *M5005_Spy_0929* | 223.760 | 0.271 | 0.299 | 0.906 | 0.470838078 |
| *M5005_Spy_1074* | 220.663 | 0.271 | 0.262 | 1.035 | 0.398611335 |
| *M5005_Spy_1096* | 55.365 | -0.271 | 0.350 | -0.774 | 0.547600253 |
| *M5005_Spy_1722* | 38.600 | 0.269 | 0.301 | 0.894 | 0.478095542 |
| *M5005_Spy_0739* | 427.983 | 0.268 | 0.193 | 1.395 | 0.238149151 |
| *sagD* | 621.473 | -0.268 | 0.313 | -0.858 | 0.500944938 |
| *M5005_Spy_0268* | 34.719 | 0.268 | 0.317 | 0.843 | 0.508538721 |
| *M5005_Spy_1475* | 195.040 | 0.267 | 0.236 | 1.132 | 0.350318274 |
| *M5005_Spy_1248* | 1814.003 | -0.267 | 0.179 | -1.495 | 0.202869635 |
| *M5005_Spy_0174* | 17.900 | -0.267 | 0.449 | -0.596 | 0.646605503 |
| *M5005_Spy_0901* | 11.256 | 0.267 | 0.497 | 0.537 | 0.684246911 |
| *M5005_Spy_1772* | 1.785 | -0.266 | 0.686 | -0.388 | NA |
| *rpe* | 207.776 | -0.266 | 0.179 | -1.482 | 0.207113495 |
| *M5005_Spy_1849* | 208.771 | -0.265 | 0.215 | -1.234 | 0.304313903 |
| *tlpA* | 6.321 | -0.265 | 0.587 | -0.451 | 0.735425533 |
| *glpF.2* | 256.891 | 0.265 | 0.234 | 1.130 | 0.350933689 |
| *M5005_Spy_1309* | 6.473 | -0.263 | 0.613 | -0.429 | 0.747743088 |
| *map* | 1258.434 | -0.263 | 0.130 | -2.026 | 0.076438473 |
| *M5005_Spy_0666* | 5.389 | -0.262 | 0.623 | -0.421 | 0.752470309 |
| *malD* | 1132.839 | -0.262 | 0.399 | -0.656 | 0.612165253 |
| *M5005_Spy_1362* | 259.229 | -0.261 | 0.167 | -1.568 | 0.179730409 |
| *M5005_Spy_1748* | 144.672 | 0.261 | 0.243 | 1.073 | 0.378907764 |
| *ung* | 74.079 | -0.261 | 0.275 | -0.947 | 0.447812604 |
| *M5005_Spy_1520* | 0.581 | 0.260 | 0.627 | 0.415 | NA |
| *epf* | 34.998 | 0.260 | 0.311 | 0.836 | 0.511986209 |
| *M5005_Spy_0393* | 240.441 | -0.259 | 0.197 | -1.316 | 0.268213211 |
| *rplP* | 2765.111 | 0.259 | 0.177 | 1.464 | 0.213061722 |
| *M5005_Spy_0685* | 295.137 | 0.259 | 0.180 | 1.434 | 0.223598288 |
| *M5005_Spy_0541* | 222.956 | -0.257 | 0.222 | -1.160 | 0.336946486 |
| *M5005_Spy_0543* | 304.458 | 0.256 | 0.279 | 0.917 | 0.463859758 |
| *M5005_Spy_0480* | 235.986 | -0.255 | 0.160 | -1.595 | 0.171472726 |
| *snf* | 479.508 | 0.255 | 0.154 | 1.656 | 0.154340385 |
| *M5005_Spy_0726* | 108.211 | 0.254 | 0.238 | 1.064 | 0.383567854 |
| *M5005_Spy_1734* | 44.472 | -0.254 | 0.305 | -0.832 | 0.514387903 |
| *M5005_Spy_1847* | 223.580 | -0.253 | 0.245 | -1.030 | 0.401223154 |
| *Gene or locus* | *Normalized count base mean* | *log2 fold change* | *Standard error* | *Wald test statistic* | *Benjamini-Hochberg adjusted P value* |
| *M5005_Spy_1549* | 227.022 | 0.252 | 0.176 | 1.433 | 0.223777265 |
| *gor* | 284.395 | -0.251 | 0.168 | -1.493 | 0.203541391 |
| *gabD* | 690.652 | 0.249 | 0.221 | 1.128 | 0.35190753 |
| *dyr* | 374.508 | -0.248 | 0.205 | -1.211 | 0.31561573 |
| *oadA2* | 18.230 | -0.248 | 0.392 | -0.632 | 0.623667149 |
| *purE* | 9.106 | 0.247 | 0.508 | 0.487 | 0.715996894 |
| *miaA* | 118.232 | -0.247 | 0.285 | -0.866 | 0.49661201 |
| *rplT* | 336.792 | 0.247 | 0.207 | 1.191 | 0.322698439 |
| *pgk* | 1279.055 | 0.246 | 0.127 | 1.933 | 0.09150994 |
| *nga* | 5639.401 | -0.245 | 0.166 | -1.477 | 0.208486762 |
| *papS* | 790.658 | 0.245 | 0.146 | 1.682 | 0.147572305 |
| *rexB* | 1157.593 | 0.244 | 0.230 | 1.063 | 0.383728096 |
| *M5005_Spy_0767* | 196.508 | 0.244 | 0.188 | 1.298 | 0.275237028 |
| *M5005_Spy_0667* | 136.976 | 0.243 | 0.328 | 0.741 | 0.565843956 |
| *frr* | 598.552 | -0.242 | 0.202 | -1.199 | 0.319265066 |
| *M5005_Spy_0958* | 35.446 | -0.239 | 0.341 | -0.701 | 0.585620922 |
| *M5005_Spy_0911* | 203.961 | -0.239 | 0.179 | -1.335 | 0.260814585 |
| *M5005_Spy_1583* | 391.171 | -0.239 | 0.211 | -1.130 | 0.35100505 |
| *M5005_Spy_0941* | 794.800 | -0.238 | 0.148 | -1.604 | 0.168781009 |
| *scrK* | 24.152 | 0.237 | 0.380 | 0.625 | 0.627660251 |
| *ftsH* | 7246.740 | 0.237 | 0.283 | 0.837 | 0.511986209 |
| *fpg* | 173.630 | 0.236 | 0.239 | 0.987 | 0.424336598 |
| *atoD.1* | 47.968 | -0.235 | 0.354 | -0.664 | 0.607381977 |
| *M5005_Spy_1608* | 875.580 | 0.235 | 0.233 | 1.010 | 0.412262155 |
| *cpsFO* | 1336.773 | -0.234 | 0.175 | -1.342 | 0.258262597 |
| *M5005_Spy_0799* | 0.172 | 0.232 | 0.386 | 0.601 | NA |
| *ftsE* | 419.233 | 0.231 | 0.232 | 0.996 | 0.419652348 |
| *recJ* | 353.611 | 0.231 | 0.191 | 1.210 | 0.315743797 |
| *M5005_Spy_0215* | 30.871 | 0.230 | 0.438 | 0.526 | 0.690891356 |
| *M5005_Spy_1387* | 1077.689 | -0.230 | 0.196 | -1.173 | 0.330745411 |
| *M5005_Spy_0593* | 269.687 | 0.228 | 0.262 | 0.872 | 0.492915499 |
| *adk* | 980.552 | -0.227 | 0.179 | -1.270 | 0.28767851 |
| *hlyX* | 1132.687 | -0.226 | 0.292 | -0.776 | 0.546116735 |
| *cysS* | 232.794 | -0.225 | 0.175 | -1.286 | 0.280451481 |
| *M5005_Spy_1862* | 321.175 | 0.224 | 0.224 | 0.999 | 0.418322115 |
| *M5005_Spy_0358* | 283.734 | -0.223 | 0.207 | -1.081 | 0.376524208 |
| *M5005_Spy_0993* | 332.974 | -0.223 | 0.189 | -1.181 | 0.327337885 |
| *M5005_Spy_0218* | 59.744 | -0.223 | 0.389 | -0.574 | 0.660617473 |
| *secE* | 442.571 | -0.220 | 0.225 | -0.979 | 0.428340931 |
| *M5005_Spy_1291* | 624.497 | 0.220 | 0.187 | 1.177 | 0.329132609 |
| *malA* | 1361.449 | -0.220 | 0.386 | -0.570 | 0.662045956 |
| *M5005_Spy_0490* | 64.877 | -0.220 | 0.237 | -0.928 | 0.458364517 |
| *M5005_Spy_0665* | 2.345 | -0.220 | 0.707 | -0.311 | 0.819847078 |
| *Gene or locus* | *Normalized count base mean* | *log2 fold change* | *Standard error* | *Wald test statistic* | *Benjamini-Hochberg adjusted P value* |
| *M5005_Spy_0459* | 1.026 | 0.219 | 0.673 | 0.326 | NA |
| *M5005_Spy_0768* | 297.536 | 0.219 | 0.203 | 1.079 | 0.377220508 |
| *scpA* | 9144.072 | 0.219 | 0.215 | 1.017 | 0.408359481 |
| *efp* | 1509.346 | -0.219 | 0.222 | -0.985 | 0.425549392 |
| *lrp* | 40.385 | 0.218 | 0.314 | 0.694 | 0.590025405 |
| *M5005_Spy_0257* | 3.908 | -0.218 | 0.657 | -0.332 | 0.807725675 |
| *M5005_Spy_1306* | 54.432 | 0.218 | 0.260 | 0.836 | 0.511986209 |
| *pdxK* | 40.999 | 0.216 | 0.303 | 0.713 | 0.580345287 |
| *hasA* | 3083.159 | 0.216 | 0.161 | 1.345 | 0.257523623 |
| *M5005_Spy_0402* | 38.611 | 0.216 | 0.292 | 0.739 | 0.5662985 |
| *M5005_Spy_1668* | 0.575 | 0.215 | 0.598 | 0.360 | NA |
| *M5005_Spy_1128* | 0.204 | -0.214 | 0.355 | -0.603 | NA |
| *M5005_Spy_1645* | 0.204 | -0.214 | 0.355 | -0.603 | NA |
| *xpt* | 44.404 | -0.214 | 0.285 | -0.750 | 0.561637627 |
| *M5005_Spy_0411* | 1170.597 | 0.212 | 0.197 | 1.075 | 0.378435004 |
| *comFA* | 14.615 | 0.212 | 0.429 | 0.493 | 0.712525483 |
| *M5005_Spy_0102* | 1.326 | -0.212 | 0.704 | -0.300 | NA |
| *M5005_Spy_0777* | 4557.596 | -0.211 | 0.128 | -1.646 | 0.156823212 |
| *salA* | 28.045 | -0.209 | 0.464 | -0.450 | 0.735425533 |
| *M5005_Spy_0354* | 505.083 | 0.209 | 0.194 | 1.075 | 0.378435004 |
| *murD* | 870.350 | 0.208 | 0.172 | 1.206 | 0.316959508 |
| *M5005_Spy_0196* | 66.459 | -0.207 | 0.267 | -0.776 | 0.546116735 |
| *lacG* | 235.390 | 0.207 | 0.173 | 1.196 | 0.320697294 |
| *M5005_Spy_0554* | 2207.730 | -0.206 | 0.138 | -1.490 | 0.204570912 |
| *M5005_Spy_1307* | 8.795 | -0.206 | 0.567 | -0.363 | 0.788321154 |
| *ligA* | 1090.019 | 0.204 | 0.184 | 1.111 | 0.360647781 |
| *M5005_Spy_0123* | 112.929 | 0.204 | 0.290 | 0.703 | 0.585365795 |
| *M5005_Spy_0724* | 182.725 | 0.202 | 0.238 | 0.850 | 0.505298124 |
| *M5005_Spy_1733* | 13.267 | 0.199 | 0.506 | 0.394 | 0.769263036 |
| *M5005_Spy_0109* | 4744.216 | -0.198 | 0.167 | -1.191 | 0.322698439 |
| *M5005_Spy_0121* | 3.190 | -0.198 | 0.662 | -0.300 | 0.827278311 |
| *arcA* | 4224.326 | -0.198 | 0.184 | -1.077 | 0.377895297 |
| *atoA* | 65.822 | 0.198 | 0.277 | 0.715 | 0.57937496 |
| *M5005_Spy_1366* | 1424.890 | -0.197 | 0.215 | -0.919 | 0.463783915 |
| *dgk* | 72.856 | -0.196 | 0.245 | -0.800 | 0.532298403 |
| *atpB* | 5301.491 | -0.196 | 0.216 | -0.909 | 0.469108786 |
| *M5005_Spy_0265* | 97.859 | -0.196 | 0.245 | -0.801 | 0.532298403 |
| *M5005_Spy_0865* | 124.526 | -0.196 | 0.239 | -0.819 | 0.522138872 |
| *ruvB* | 921.947 | -0.195 | 0.148 | -1.318 | 0.267877755 |
| *M5005_Spy_1787* | 8.167 | 0.193 | 0.555 | 0.349 | 0.797397648 |
| *M5005_Spy_0326* | 339.302 | -0.193 | 0.246 | -0.785 | 0.541591086 |
| *fabZ* | 675.337 | 0.191 | 0.200 | 0.957 | 0.441394284 |
| *M5005_Spy_0646* | 3919.989 | 0.191 | 0.178 | 1.075 | 0.378435353 |
| *Gene or locus* | *Normalized count base mean* | *log2 fold change* | *Standard error* | *Wald test statistic* | *Benjamini-Hochberg adjusted P value* |
| *M5005_Spy_0985* | 188.695 | -0.191 | 0.278 | -0.687 | 0.594209005 |
| *eftLSL.B* | 3610.044 | 0.190 | 0.141 | 1.347 | 0.256568686 |
| *agaD* | 19.934 | -0.189 | 0.500 | -0.378 | 0.780353112 |
| *M5005_Spy_0518* | 6.066 | -0.189 | 0.618 | -0.306 | 0.823646541 |
| *M5005_Spy_0198* | 42.728 | -0.189 | 0.282 | -0.669 | 0.604819209 |
| *pyrR* | 202.180 | 0.187 | 0.309 | 0.604 | 0.641079044 |
| *relA* | 1179.077 | 0.187 | 0.242 | 0.772 | 0.548306604 |
| *M5005_Spy_1150* | 87.488 | -0.186 | 0.249 | -0.745 | 0.563377843 |
| *folE* | 1047.736 | 0.185 | 0.160 | 1.157 | 0.337616166 |
| *M5005_Spy_0353* | 16.283 | -0.185 | 0.422 | -0.437 | 0.743523634 |
| *M5005_Spy_1588* | 27.924 | -0.184 | 0.373 | -0.494 | 0.712525483 |
| *M5005_Spy_1819* | 63.858 | -0.184 | 0.266 | -0.693 | 0.590455043 |
| *M5005_Spy_0289* | 1021.531 | -0.183 | 0.164 | -1.119 | 0.356429967 |
| *folC.1* | 1355.679 | -0.183 | 0.134 | -1.364 | 0.250219303 |
| *M5005_Spy_1400* | 195.048 | 0.182 | 0.182 | 1.003 | 0.416130648 |
| *nifU* | 433.247 | -0.182 | 0.169 | -1.079 | 0.377220508 |
| *M5005_Spy_0967* | 50.411 | 0.182 | 0.278 | 0.654 | 0.612713086 |
| *radC* | 3.910 | 0.181 | 0.644 | 0.281 | 0.837938696 |
| *M5005_Spy_1729* | 710.713 | 0.181 | 0.135 | 1.338 | 0.259579752 |
| *M5005_Spy_1305* | 23.356 | 0.180 | 0.379 | 0.475 | 0.722355525 |
| *proV* | 186.219 | 0.180 | 0.212 | 0.848 | 0.506285258 |
| *M5005_Spy_1614* | 356.708 | 0.179 | 0.212 | 0.846 | 0.507455737 |
| *M5005_Spy_0460* | 0.575 | 0.178 | 0.528 | 0.338 | NA |
| *M5005_Spy_0706* | 806.372 | 0.177 | 0.187 | 0.944 | 0.449131006 |
| *deaD2* | 279.774 | -0.176 | 0.178 | -0.993 | 0.420998276 |
| *M5005_Spy_0430* | 28.857 | 0.176 | 0.376 | 0.467 | 0.725556045 |
| *M5005_Spy_1265* | 142.786 | 0.175 | 0.234 | 0.747 | 0.562619176 |
| *M5005_Spy_0142* | 70.384 | -0.174 | 0.283 | -0.617 | 0.632910573 |
| *idnO* | 26.094 | -0.173 | 0.382 | -0.453 | 0.734446727 |
| *bglA* | 34.135 | -0.172 | 0.381 | -0.450 | 0.735425533 |
| *M5005_Spy_0419* | 84.704 | 0.171 | 0.225 | 0.762 | 0.554508075 |
| *alr* | 495.900 | 0.171 | 0.170 | 1.006 | 0.414596941 |
| *M5005_Spy_0469* | 65.835 | -0.171 | 0.269 | -0.638 | 0.621646502 |
| *M5005_Spy_0779* | 492.549 | -0.171 | 0.218 | -0.782 | 0.543144736 |
| *hsdS* | 287.600 | 0.169 | 0.166 | 1.022 | 0.40540317 |
| *M5005_Spy_1674* | 618.911 | 0.169 | 0.164 | 1.034 | 0.398895 |
| *M5005_Spy_0789* | 88.360 | 0.169 | 0.306 | 0.552 | 0.673995976 |
| *sagH* | 423.303 | 0.166 | 0.285 | 0.583 | 0.653984048 |
| *M5005_Spy_0475* | 84.407 | -0.166 | 0.238 | -0.700 | 0.586260895 |
| *ftsK* | 663.348 | 0.166 | 0.251 | 0.662 | 0.608993625 |
| *M5005_Spy_1079* | 22.379 | -0.165 | 0.445 | -0.370 | 0.783721027 |
| *M5005_Spy_1717* | 40.807 | 0.162 | 0.465 | 0.348 | 0.79750106 |
| *argR2* | 192.279 | -0.162 | 0.188 | -0.861 | 0.499101228 |
| *Gene or locus* | *Normalized count base mean* | *log2 fold change* | *Standard error* | *Wald test statistic* | *Benjamini-Hochberg adjusted P value* |
| *agaS* | 61.369 | -0.161 | 0.246 | -0.654 | 0.612713086 |
| *folP* | 654.607 | 0.160 | 0.196 | 0.820 | 0.521545063 |
| *rpiA* | 713.889 | -0.159 | 0.254 | -0.627 | 0.626823405 |
| *lctO* | 751.353 | -0.158 | 0.654 | -0.241 | 0.860170884 |
| *M5005_Spy_1776* | 4.511 | 0.157 | 0.644 | 0.243 | 0.860170884 |
| *M5005_Spy_0526* | 23.933 | -0.156 | 0.383 | -0.408 | 0.760693567 |
| *clpX* | 1197.964 | 0.156 | 0.195 | 0.801 | 0.532298403 |
| *M5005_Spy_0190* | 17.033 | 0.155 | 0.427 | 0.363 | 0.788321154 |
| *M5005_Spy_0114* | 9.513 | 0.155 | 0.526 | 0.294 | 0.830190712 |
| *M5005_Spy_1760* | 2.157 | -0.155 | 0.694 | -0.223 | NA |
| *ftsL* | 111.960 | -0.153 | 0.336 | -0.455 | 0.733934682 |
| *M5005_Spy_0391* | 281.271 | 0.153 | 0.209 | 0.733 | 0.569590715 |
| *M5005_Spy_0295* | 1064.038 | -0.153 | 0.234 | -0.653 | 0.61273831 |
| *M5005_Spy_0924* | 193.951 | 0.153 | 0.206 | 0.742 | 0.56524498 |
| *M5005_Spy_0866* | 104.973 | 0.152 | 0.273 | 0.558 | 0.670263269 |
| *M5005_Spy_1225* | 955.122 | 0.152 | 0.132 | 1.155 | 0.33864865 |
| *rpoC* | 1374.135 | -0.151 | 0.163 | -0.930 | 0.457710275 |
| *amyA* | 8870.082 | 0.149 | 0.421 | 0.354 | 0.795309973 |
| *M5005_Spy_1839* | 214.544 | 0.149 | 0.234 | 0.638 | 0.621646502 |
| *M5005_Spy_1834* | 70.046 | 0.148 | 0.262 | 0.566 | 0.664195227 |
| *parE* | 1504.258 | 0.148 | 0.195 | 0.758 | 0.556339415 |
| *sagF* | 366.956 | 0.147 | 0.296 | 0.497 | 0.711054164 |
| *potA* | 606.261 | -0.147 | 0.141 | -1.043 | 0.394655631 |
| *accC* | 4328.270 | 0.147 | 0.193 | 0.757 | 0.556621482 |
| *acpP.2* | 13.944 | -0.146 | 0.441 | -0.331 | 0.807725675 |
| *rpmG* | 1740.385 | 0.146 | 0.245 | 0.596 | 0.64646575 |
| *nadC* | 6.237 | -0.145 | 0.564 | -0.257 | 0.851627583 |
| *M5005_Spy_0764* | 9.598 | -0.144 | 0.513 | -0.281 | 0.837938696 |
| *M5005_Spy_0957* | 208.611 | 0.144 | 0.203 | 0.707 | 0.584224037 |
| *mvaK2* | 171.950 | 0.143 | 0.285 | 0.503 | 0.707588045 |
| *M5005_Spy_0879* | 393.469 | 0.143 | 0.195 | 0.734 | 0.569590715 |
| *M5005_Spy_0896* | 8.466 | 0.143 | 0.618 | 0.231 | 0.866648156 |
| *oppF* | 566.989 | 0.143 | 0.239 | 0.598 | 0.64564798 |
| *hisS* | 435.544 | -0.141 | 0.161 | -0.878 | 0.488459622 |
| *M5005_Spy_0921* | 298.790 | -0.141 | 0.175 | -0.804 | 0.530838817 |
| *lacD.2* | 461.366 | -0.141 | 0.215 | -0.656 | 0.612165253 |
| *parC* | 791.862 | 0.140 | 0.163 | 0.861 | 0.499101228 |
| *engA* | 513.373 | -0.140 | 0.184 | -0.759 | 0.555931575 |
| *lacC.2* | 413.815 | 0.140 | 0.337 | 0.415 | 0.756193117 |
| *M5005_Spy_1319* | 382.707 | -0.139 | 0.182 | -0.765 | 0.553585551 |
| *M5005_Spy_0155* | 215.475 | -0.139 | 0.175 | -0.795 | 0.535505957 |
| *M5005_Spy_1371* | 261.303 | -0.139 | 0.198 | -0.704 | 0.585365795 |
| *satD* | 54.176 | 0.139 | 0.293 | 0.474 | 0.722355525 |
| *Gene or locus* | *Normalized count base mean* | *log2 fold change* | *Standard error* | *Wald test statistic* | *Benjamini-Hochberg adjusted P value* |
| *cbiO* | 706.164 | 0.138 | 0.233 | 0.595 | 0.646900987 |
| *M5005_Spy_1407* | 2020.171 | 0.138 | 0.218 | 0.634 | 0.622912995 |
| *M5005_Spy_0183* | 15.566 | -0.138 | 0.417 | -0.332 | 0.807725675 |
| *M5005_Spy_1759* | 2.147 | -0.137 | 0.698 | -0.197 | NA |
| *rpsP* | 286.129 | -0.137 | 0.216 | -0.636 | 0.622681736 |
| *M5005_Spy_0887* | 0.480 | 0.136 | 0.582 | 0.234 | NA |
| *M5005_Spy_0802* | 0.131 | 0.136 | 0.301 | 0.453 | NA |
| *M5005_Spy_1713* | 0.131 | 0.136 | 0.301 | 0.453 | NA |
| *M5005_Spy_1736* | 0.131 | 0.136 | 0.301 | 0.453 | NA |
| *rnhB* | 1536.185 | 0.136 | 0.286 | 0.474 | 0.722355525 |
| *M5005_Spy_0839* | 929.709 | -0.136 | 0.229 | -0.593 | 0.647603191 |
| *deaD* | 1069.397 | -0.135 | 0.255 | -0.532 | 0.687818203 |
| *M5005_Spy_1795* | 215.443 | -0.134 | 0.244 | -0.550 | 0.674663648 |
| *M5005_Spy_1477* | 120.514 | 0.134 | 0.206 | 0.650 | 0.614021546 |
| *atoB* | 70.135 | 0.133 | 0.301 | 0.441 | 0.741210721 |
| *M5005_Spy_1709* | 997.996 | 0.133 | 0.170 | 0.781 | 0.543144736 |
| *perR* | 414.622 | 0.132 | 0.248 | 0.531 | 0.687984801 |
| *mutS2* | 866.569 | 0.132 | 0.143 | 0.918 | 0.463859758 |
| *thyA* | 234.364 | -0.130 | 0.264 | -0.493 | 0.712525483 |
| *M5005_Spy_0644* | 3970.336 | 0.130 | 0.154 | 0.848 | 0.506549774 |
| *murB* | 391.789 | -0.129 | 0.181 | -0.713 | 0.580345287 |
| *M5005_Spy_0818* | 540.836 | 0.128 | 0.193 | 0.665 | 0.607381977 |
| *srtE* | 11.852 | -0.128 | 0.571 | -0.225 | 0.86915497 |
| *rpsH* | 1094.493 | 0.128 | 0.185 | 0.694 | 0.590249802 |
| *rgpCc* | 322.832 | 0.128 | 0.299 | 0.428 | 0.748268719 |
| *M5005_Spy_0478* | 98.466 | -0.128 | 0.239 | -0.534 | 0.686053648 |
| *deoC* | 649.867 | -0.127 | 0.388 | -0.328 | NA |
| *M5005_Spy_1825* | 23.267 | -0.126 | 0.360 | -0.350 | 0.796727175 |
| *arcC* | 1427.066 | -0.126 | 0.221 | -0.570 | 0.662045956 |
| *M5005_Spy_1061* | 50.558 | 0.125 | 0.270 | 0.464 | 0.727412002 |
| *M5005_Spy_1118* | 1643.991 | -0.123 | 0.283 | -0.433 | 0.745361942 |
| *M5005_Spy_1323* | 2.145 | -0.122 | 0.702 | -0.174 | NA |
| *sagI* | 649.234 | 0.122 | 0.327 | 0.371 | 0.783721027 |
| *M5005_Spy_1098* | 152.004 | 0.121 | 0.187 | 0.647 | 0.615231657 |
| *M5005_Spy_1235* | 3256.276 | -0.120 | 0.186 | -0.647 | NA |
| *msmK* | 149.617 | -0.120 | 0.286 | -0.421 | 0.752470309 |
| *M5005_Spy_1503* | 575.763 | -0.119 | 0.150 | -0.792 | 0.537156302 |
| *M5005_Spy_0942* | 4230.028 | -0.118 | 0.217 | -0.544 | 0.67944042 |
| *M5005_Spy_0360* | 279.171 | -0.117 | 0.162 | -0.724 | 0.574417642 |
| *M5005_Spy_1114* | 28.978 | 0.117 | 0.332 | 0.352 | 0.796100351 |
| *glmM* | 768.729 | 0.116 | 0.170 | 0.679 | 0.598180199 |
| *gldA* | 80.641 | 0.114 | 0.285 | 0.399 | 0.766414154 |
| *M5005_Spy_1648* | 20.487 | -0.112 | 0.466 | -0.241 | 0.860170884 |
| *Gene or locus* | *Normalized count base mean* | *log2 fold change* | *Standard error* | *Wald test statistic* | *Benjamini-Hochberg adjusted P value* |
| *rgpEc* | 413.603 | 0.112 | 0.232 | 0.484 | 0.717738635 |
| *aroF* | 729.530 | -0.111 | 0.161 | -0.692 | 0.590496638 |
| *M5005_Spy_1312* | 0.621 | -0.111 | 0.600 | -0.185 | NA |
| *ftsW* | 553.039 | 0.111 | 0.153 | 0.725 | 0.574417642 |
| *dpiB* | 30.346 | 0.110 | 0.340 | 0.323 | 0.812397024 |
| *dacA2* | 169.942 | -0.110 | 0.231 | -0.474 | 0.722355525 |
| *M5005_Spy_1410* | 723.850 | 0.109 | 0.160 | 0.686 | 0.59420971 |
| *pbp1b* | 1029.593 | 0.109 | 0.188 | 0.580 | 0.656448825 |
| *M5005_Spy_0693* | 495.407 | -0.108 | 0.171 | -0.632 | 0.623667149 |
| *M5005_Spy_0312* | 95.334 | 0.108 | 0.246 | 0.439 | 0.74284704 |
| *tmk* | 196.888 | -0.108 | 0.183 | -0.587 | 0.651556854 |
| *M5005_Spy_0184* | 2.025 | 0.106 | 0.706 | 0.150 | NA |
| *nrdH* | 117.130 | 0.106 | 0.202 | 0.525 | 0.691121833 |
| *yvqE* | 343.936 | 0.106 | 0.150 | 0.703 | 0.585365795 |
| *fus* | 4798.626 | 0.105 | 0.199 | 0.529 | 0.688536844 |
| *rgpBc* | 244.996 | -0.104 | 0.281 | -0.370 | 0.783721027 |
| *M5005_Spy_1130* | 80.305 | 0.104 | 0.252 | 0.412 | 0.758010555 |
| *citD* | 23.501 | 0.103 | 0.401 | 0.258 | 0.851448603 |
| *M5005_Spy_0926* | 1649.192 | -0.103 | 0.135 | -0.762 | 0.554508075 |
| *M5005_Spy_1710* | 793.474 | 0.103 | 0.180 | 0.573 | 0.661137197 |
| *pepD* | 146.544 | 0.103 | 0.209 | 0.492 | 0.712538254 |
| *M5005_Spy_1224* | 169.092 | 0.102 | 0.215 | 0.475 | 0.722355525 |
| *fhs.2* | 2.488 | 0.102 | 0.681 | 0.149 | 0.91560047 |
| *ntpD* | 43.156 | -0.101 | 0.367 | -0.275 | 0.841719983 |
| *epuA* | 92.500 | -0.101 | 0.267 | -0.376 | 0.780353112 |
| *M5005_Spy_0800* | 0.438 | 0.100 | 0.568 | 0.175 | NA |
| *fabF* | 2374.569 | 0.099 | 0.224 | 0.443 | 0.740038539 |
| *plsX* | 1670.692 | -0.099 | 0.146 | -0.677 | 0.598755428 |
| *M5005_Spy_1126* | 2.872 | -0.099 | 0.707 | -0.140 | 0.921102476 |
| *dnaI* | 295.565 | -0.098 | 0.210 | -0.467 | 0.725556045 |
| *M5005_Spy_0499* | 26.429 | -0.097 | 0.403 | -0.241 | 0.860170884 |
| *M5005_Spy_0236* | 233.225 | 0.097 | 0.197 | 0.492 | 0.712538254 |
| *M5005_Spy_1478* | 261.059 | -0.096 | 0.184 | -0.525 | 0.690891356 |
| *cadC* | 38.631 | -0.096 | 0.296 | -0.323 | 0.812397024 |
| *M5005_Spy_0773* | 0.326 | -0.095 | 0.513 | -0.185 | NA |
| *M5005_Spy_1509* | 0.326 | -0.095 | 0.513 | -0.185 | NA |
| *comYD* | 0.326 | -0.095 | 0.513 | -0.185 | NA |
| *prgA* | 3901.953 | 0.093 | 0.154 | 0.607 | 0.639065698 |
| *M5005_Spy_0816* | 41.092 | 0.093 | 0.291 | 0.319 | 0.814567655 |
| *M5005_Spy_1342* | 2823.501 | -0.091 | 0.190 | -0.481 | 0.719554636 |
| *mipB* | 158.015 | -0.091 | 0.240 | -0.377 | 0.780353112 |
| *scrA* | 116.128 | 0.090 | 0.257 | 0.350 | 0.796727175 |
| *fabG* | 1250.007 | -0.088 | 0.227 | -0.390 | 0.771801073 |
| *Gene or locus* | *Normalized count base mean* | *log2 fold change* | *Standard error* | *Wald test statistic* | *Benjamini-Hochberg adjusted P value* |
| *M5005_Spy_0379* | 241.301 | 0.087 | 0.309 | 0.283 | 0.837314208 |
| *pulA* | 61.192 | -0.087 | 0.320 | -0.271 | 0.844041628 |
| *ftsZ* | 2559.127 | -0.085 | 0.182 | -0.467 | 0.725556045 |
| *M5005_Spy_1747* | 170.158 | -0.085 | 0.194 | -0.435 | 0.744805606 |
| *lmb* | 180.875 | -0.084 | 0.200 | -0.419 | 0.753163809 |
| *M5005_Spy_1299* | 0.328 | -0.083 | 0.474 | -0.176 | NA |
| *M5005_Spy_0246* | 384.735 | -0.082 | 0.189 | -0.432 | 0.745952756 |
| *M5005_Spy_1084* | 30.324 | 0.082 | 0.359 | 0.227 | 0.868713886 |
| *M5005_Spy_0761* | 141.175 | 0.081 | 0.192 | 0.423 | 0.751505048 |
| *M5005_Spy_0838* | 116.432 | -0.079 | 0.206 | -0.382 | 0.777332532 |
| *M5005_Spy_0898* | 20.529 | 0.079 | 0.397 | 0.198 | 0.886504828 |
| *dnaX* | 397.763 | -0.078 | 0.163 | -0.481 | 0.719554636 |
| *M5005_Spy_0843* | 64.147 | 0.078 | 0.256 | 0.304 | 0.824062429 |
| *M5005_Spy_0156* | 79.749 | 0.077 | 0.356 | 0.218 | 0.873315048 |
| *M5005_Spy_1156* | 771.058 | 0.077 | 0.178 | 0.433 | 0.745361942 |
| *holB* | 1820.161 | 0.076 | 0.137 | 0.556 | 0.670860788 |
| *hutH* | 5.268 | -0.076 | 0.623 | -0.121 | 0.931000944 |
| *M5005_Spy_0455* | 2.462 | 0.075 | 0.688 | 0.109 | 0.93689568 |
| *nrdE.1* | 127.657 | -0.075 | 0.260 | -0.288 | 0.834281037 |
| *aroE* | 114.077 | 0.074 | 0.201 | 0.370 | 0.783721027 |
| *recO* | 130.703 | -0.074 | 0.188 | -0.395 | 0.768521687 |
| *M5005_Spy_0103* | 219.137 | 0.074 | 0.190 | 0.390 | 0.771801073 |
| *fasC* | 215.194 | -0.074 | 0.238 | -0.311 | 0.819847078 |
| *mac* | 1083.850 | -0.074 | 0.158 | -0.468 | 0.725556045 |
| *greA* | 269.743 | -0.072 | 0.258 | -0.277 | 0.840809176 |
| *M5005_Spy_0507* | 0.332 | -0.072 | 0.475 | -0.151 | NA |
| *M5005_Spy_0674* | 816.364 | 0.071 | 0.177 | 0.399 | 0.766279343 |
| *sagG* | 316.339 | 0.070 | 0.263 | 0.268 | 0.846173495 |
| *M5005_Spy_0352* | 1071.056 | -0.070 | 0.267 | -0.261 | 0.849792407 |
| *M5005_Spy_0812* | 1.481 | -0.069 | 0.702 | -0.098 | NA |
| *amiC* | 332.365 | 0.069 | 0.220 | 0.314 | 0.818020222 |
| *rplE* | 790.911 | -0.069 | 0.170 | -0.406 | 0.76162689 |
| *M5005_Spy_0720* | 90.822 | 0.068 | 0.236 | 0.289 | 0.833858453 |
| *M5005_Spy_0113* | 7.671 | 0.067 | 0.530 | 0.127 | 0.927285457 |
| *M5005_Spy_0191* | 109.201 | 0.067 | 0.266 | 0.254 | 0.853661317 |
| *M5005_Spy_0235* | 143.036 | -0.067 | 0.255 | -0.264 | 0.848592179 |
| *rgpG* | 3.263 | 0.067 | 0.697 | 0.096 | 0.945987959 |
| *crgR* | 151.424 | 0.066 | 0.192 | 0.347 | 0.797881895 |
| *adcB* | 144.860 | -0.066 | 0.197 | -0.337 | 0.80418775 |
| *M5005_Spy_1313* | 85.917 | 0.066 | 0.327 | 0.202 | 0.884504812 |
| *M5005_Spy_0979* | 1.117 | -0.065 | 0.697 | -0.094 | NA |
| *M5005_Spy_1469* | 848.904 | -0.065 | 0.124 | -0.522 | 0.692751838 |
| *M5005_Spy_1331* | 1453.730 | -0.064 | 0.128 | -0.497 | 0.711063181 |
| *Gene or locus* | *Normalized count base mean* | *log2 fold change* | *Standard error* | *Wald test statistic* | *Benjamini-Hochberg adjusted P value* |
| *M5005_Spy_1129* | 84.347 | 0.063 | 0.238 | 0.262 | 0.848729763 |
| *M5005_Spy_1657* | 17.900 | -0.062 | 0.417 | -0.149 | 0.91560047 |
| *malF* | 134.334 | 0.062 | 0.233 | 0.265 | 0.848145953 |
| *lepA* | 890.326 | 0.061 | 0.176 | 0.346 | 0.79805611 |
| *M5005_Spy_0895* | 18.970 | 0.060 | 0.402 | 0.150 | 0.91560047 |
| *M5005_Spy_0254* | 0.307 | -0.060 | 0.450 | -0.134 | NA |
| *M5005_Spy_1246* | 270.968 | 0.060 | 0.271 | 0.221 | 0.871810965 |
| *M5005_Spy_1537* | 52.052 | 0.060 | 0.264 | 0.227 | 0.868713886 |
| *M5005_Spy_0023* | 34.707 | -0.059 | 0.376 | -0.158 | 0.910643682 |
| *prfC* | 2014.336 | 0.059 | 0.173 | 0.340 | 0.802444012 |
| *M5005_Spy_1162* | 97.441 | -0.058 | 0.242 | -0.241 | 0.860170884 |
| *M5005_Spy_1580* | 115.136 | -0.058 | 0.220 | -0.263 | 0.848621632 |
| *M5005_Spy_0583* | 609.958 | 0.057 | 0.143 | 0.402 | 0.764333716 |
| *pepB* | 629.731 | -0.057 | 0.231 | -0.245 | 0.859826003 |
| *M5005_Spy_0522* | 78.000 | -0.056 | 0.490 | -0.114 | 0.935561725 |
| *M5005_Spy_1080* | 26.134 | -0.055 | 0.498 | -0.111 | 0.93689568 |
| *M5005_Spy_0984* | 354.709 | -0.055 | 0.230 | -0.240 | 0.860270601 |
| *bglA.2* | 17.975 | -0.054 | 0.415 | -0.131 | 0.925523642 |
| *M5005_Spy_1511* | 7.306 | 0.054 | 0.586 | 0.093 | 0.947885225 |
| *M5005_Spy_0223* | 304.595 | 0.052 | 0.255 | 0.202 | 0.884504812 |
| *fms* | 15.551 | -0.051 | 0.466 | -0.110 | 0.93689568 |
| *proS* | 940.350 | 0.050 | 0.157 | 0.319 | 0.814567655 |
| *purD* | 40.752 | -0.049 | 0.302 | -0.162 | 0.909454392 |
| *mvaK1* | 170.724 | 0.048 | 0.246 | 0.197 | 0.886878454 |
| *nanH* | 49.037 | -0.045 | 0.317 | -0.143 | 0.919702215 |
| *truA* | 1020.626 | -0.043 | 0.240 | -0.178 | 0.899560012 |
| *M5005_Spy_0418* | 119.774 | -0.042 | 0.211 | -0.200 | 0.885479786 |
| *M5005_Spy_0557* | 14.658 | -0.042 | 0.499 | -0.084 | 0.953480945 |
| *M5005_Spy_1651* | 581.886 | 0.041 | 0.150 | 0.273 | 0.843116739 |
| *xseA* | 443.293 | -0.040 | 0.249 | -0.162 | 0.909454392 |
| *M5005_Spy_1664* | 6.909 | -0.040 | 0.559 | -0.072 | 0.960094539 |
| *lmrP* | 226.480 | -0.040 | 0.232 | -0.172 | 0.903201474 |
| *M5005_Spy_1350* | 700.321 | 0.040 | 0.190 | 0.208 | 0.880618043 |
| *M5005_Spy_1501* | 187.893 | -0.039 | 0.179 | -0.219 | 0.873181575 |
| *M5005_Spy_0160* | 349.776 | -0.039 | 0.158 | -0.246 | 0.859258909 |
| *M5005_Spy_0108* | 3960.446 | 0.039 | 0.170 | 0.228 | 0.868713886 |
| *M5005_Spy_0381* | 252.871 | -0.039 | 0.268 | -0.144 | 0.919325171 |
| *polA* | 760.835 | -0.038 | 0.251 | -0.153 | 0.914341642 |
| *M5005_Spy_0983* | 203.751 | -0.037 | 0.233 | -0.158 | 0.910643682 |
| *oppB* | 392.666 | 0.036 | 0.204 | 0.177 | 0.899560012 |
| *trcF* | 1024.873 | 0.036 | 0.222 | 0.161 | 0.909454392 |
| *M5005_Spy_0649* | 33.212 | 0.035 | 0.308 | 0.115 | 0.935561725 |
| *gatA* | 357.340 | 0.034 | 0.152 | 0.226 | 0.868767639 |
| *Gene or locus* | *Normalized count base mean* | *log2 fold change* | *Standard error* | *Wald test statistic* | *Benjamini-Hochberg adjusted P value* |
| *M5005_Spy_0539* | 650.763 | 0.034 | 0.184 | 0.186 | 0.894792641 |
| *M5005_Spy_0341* | 5938.969 | 0.033 | 0.311 | 0.105 | 0.940202992 |
| *rplM* | 901.625 | 0.032 | 0.182 | 0.177 | 0.899560012 |
| *pbp1A* | 2429.085 | 0.031 | 0.176 | 0.178 | 0.899560012 |
| *M5005_Spy_1290* | 136.708 | 0.031 | 0.232 | 0.133 | 0.925288039 |
| *M5005_Spy_1796* | 1275.498 | 0.031 | 0.187 | 0.164 | 0.908461125 |
| *gyrA* | 1331.200 | -0.029 | 0.151 | -0.195 | 0.888036854 |
| *M5005_Spy_1671* | 152.534 | 0.029 | 0.220 | 0.134 | 0.925288039 |
| *pyrC* | 294.965 | 0.029 | 0.179 | 0.160 | 0.909664981 |
| *citE* | 38.585 | -0.029 | 0.379 | -0.075 | 0.957778052 |
| *M5005_Spy_1699* | 153.849 | -0.028 | 0.256 | -0.110 | 0.93689568 |
| *M5005_Spy_1266* | 869.787 | -0.027 | 0.147 | -0.185 | 0.895306437 |
| *spyA* | 391.922 | 0.027 | 0.202 | 0.132 | 0.925288039 |
| *M5005_Spy_0819* | 0.262 | 0.026 | 0.134 | 0.196 | NA |
| *M5005_Spy_0406* | 2.008 | 0.024 | 0.698 | 0.035 | NA |
| *prfA* | 253.648 | -0.024 | 0.183 | -0.130 | 0.925673416 |
| *ddh* | 3.252 | 0.023 | 0.699 | 0.033 | 0.985944595 |
| *M5005_Spy_0304* | 361.072 | -0.023 | 0.280 | -0.083 | 0.953480945 |
| *M5005_Spy_0167* | 1.156 | -0.021 | 0.692 | -0.030 | NA |
| *purB* | 157.270 | 0.019 | 0.237 | 0.081 | 0.954743812 |
| *nifS3* | 365.598 | -0.019 | 0.153 | -0.125 | 0.928565997 |
| *hasC* | 1806.156 | -0.018 | 0.188 | -0.097 | 0.945987959 |
| *M5005_Spy_0014* | 282.122 | 0.018 | 0.163 | 0.110 | 0.93689568 |
| *M5005_Spy_0933* | 297.812 | -0.018 | 0.223 | -0.080 | 0.954901834 |
| *rpsI* | 218.567 | 0.016 | 0.173 | 0.092 | 0.947885225 |
| *M5005_Spy_0737* | 220.983 | -0.015 | 0.248 | -0.062 | 0.965432506 |
| *M5005_Spy_1311* | 5.481 | 0.015 | 0.589 | 0.025 | 0.98872227 |
| *M5005_Spy_0410* | 595.173 | -0.013 | 0.172 | -0.077 | 0.957329649 |
| *M5005_Spy_1102* | 534.688 | 0.013 | 0.148 | 0.088 | 0.950817368 |
| *M5005_Spy_0213* | 116.995 | -0.013 | 0.526 | -0.024 | 0.98872227 |
| *nrdF* | 786.376 | 0.012 | 0.191 | 0.065 | 0.964178642 |
| *aroD* | 509.711 | -0.012 | 0.184 | -0.067 | 0.963118643 |
| *accD* | 603.518 | 0.012 | 0.200 | 0.061 | 0.965580948 |
| *M5005_Spy_1773* | 1.100 | 0.012 | 0.683 | 0.017 | NA |
| *M5005_Spy_0247* | 1576.114 | -0.011 | 0.277 | -0.039 | 0.981874012 |
| *murF* | 396.445 | -0.011 | 0.152 | -0.071 | 0.960314646 |
| *nrdI* | 25.486 | 0.010 | 0.361 | 0.027 | 0.98872227 |
| *M5005_Spy_0338* | 19.100 | -0.009 | 0.488 | -0.019 | 0.991467718 |
| *M5005_Spy_0811* | 4.891 | -0.009 | 0.640 | -0.015 | 0.994680169 |
| *M5005_Spy_0226* | 463.537 | -0.009 | 0.151 | -0.062 | 0.965432506 |
| *M5005_Spy_1656* | 45.639 | -0.009 | 0.364 | -0.025 | 0.98872227 |
| *M5005_Spy_1581* | 42.775 | 0.009 | 0.328 | 0.027 | 0.98872227 |
| *murM* | 435.651 | -0.008 | 0.142 | -0.054 | 0.970381624 |
| *Gene or locus* | *Normalized count base mean* | *log2 fold change* | *Standard error* | *Wald test statistic* | *Benjamini-Hochberg adjusted P value* |
| *M5005_Spy_1653* | 240.434 | 0.007 | 0.271 | 0.027 | 0.98872227 |
| *M5005_Spy_0710* | 140.567 | 0.006 | 0.214 | 0.027 | 0.98872227 |
| *coaD* | 90.885 | 0.005 | 0.229 | 0.021 | 0.990464142 |
| *M5005_Spy_0645* | 1196.186 | 0.004 | 0.159 | 0.025 | 0.98872227 |
| *M5005_Spy_1654* | 188.493 | -0.004 | 0.285 | -0.013 | 0.995068212 |
| *polC* | 1345.221 | -0.003 | 0.218 | -0.014 | 0.994961459 |
| *M5005_Spy_1300* | 61.665 | 0.003 | 0.269 | 0.011 | 0.995068212 |
| *M5005_Spy_1268* | 367.775 | 0.002 | 0.197 | 0.012 | 0.995068212 |
| *mtsC* | 310.253 | -0.002 | 0.203 | -0.011 | 0.995068212 |
| *M5005_Spy_0934* | 194.985 | -0.002 | 0.279 | -0.007 | 0.997436568 |
| *M5005_Spy_1359* | 933.401 | 0.001 | 0.203 | 0.006 | 0.997436568 |
| *artQ* | 48.387 | 0.001 | 0.285 | 0.004 | 0.998025109 |
| *ileS* | 660.041 | 0.001 | 0.235 | 0.004 | 0.998025109 |
| *ntpE* | 21.914 | -0.001 | 0.374 | -0.003 | 0.998573391 |
| *fabD* | 1231.094 | 0.000 | 0.243 | -0.002 | 0.998786984 |
| *M5005_Spy_0015* | 0.000 | NA | NA | NA | NA |
| *M5005_Spy_0072* | 0.000 | NA | NA | NA | NA |
| *M5005_Spy_0145* | 0.000 | NA | NA | NA | NA |
| *M5005_Spy_0172* | 0.000 | NA | NA | NA | NA |
| *M5005_Spy_0187* | 0.000 | NA | NA | NA | NA |
| *M5005_Spy_0188* | 0.000 | NA | NA | NA | NA |
| *M5005_Spy_0255* | 0.000 | NA | NA | NA | NA |
| *M5005_Spy_0258* | 0.000 | NA | NA | NA | NA |
| *M5005_Spy_0297* | 0.000 | NA | NA | NA | NA |
| *M5005_Spy_0298* | 0.000 | NA | NA | NA | NA |
| *M5005_Spy_0350* | 0.000 | NA | NA | NA | NA |
| *M5005_Spy_0376* | 0.000 | NA | NA | NA | NA |
| *M5005_Spy_0395* | 0.000 | NA | NA | NA | NA |
| *M5005_Spy_0396* | 0.000 | NA | NA | NA | NA |
| *M5005_Spy_0405* | 0.000 | NA | NA | NA | NA |
| *M5005_Spy_0481* | 0.000 | NA | NA | NA | NA |
| *M5005_Spy_0650* | 0.000 | NA | NA | NA | NA |
| *M5005_Spy_0675* | 0.000 | NA | NA | NA | NA |
| *M5005_Spy_0797* | 0.000 | NA | NA | NA | NA |
| *M5005_Spy_0801* | 0.000 | NA | NA | NA | NA |
| *M5005_Spy_1090* | 0.000 | NA | NA | NA | NA |
| *M5005_Spy_1143* | 0.000 | NA | NA | NA | NA |
| *M5005_Spy_1324* | 0.000 | NA | NA | NA | NA |
| *M5005_Spy_1536* | 0.000 | NA | NA | NA | NA |
| *M5005_Spy_1605* | 0.000 | NA | NA | NA | NA |
| *M5005_Spy_1619* | 0.000 | NA | NA | NA | NA |
| *M5005_Spy_1642* | 0.000 | NA | NA | NA | NA |
| *M5005_Spy_1665* | 0.000 | NA | NA | NA | NA |
| *Gene or locus* | *Normalized count base mean* | *log2 fold change* | *Standard error* | *Wald test statistic* | *Benjamini-Hochberg adjusted P value* |
| *M5005_Spy_1712* | 0.000 | NA | NA | NA | NA |
| *M5005_Spy_1739* | 0.000 | NA | NA | NA | NA |
| *M5005_Spy_1766* | 0.000 | NA | NA | NA | NA |
| *M5005_Spy_1767* | 0.000 | NA | NA | NA | NA |
| *M5005_Spy_1833* | 0.000 | NA | NA | NA | NA |
| *comX.2* | 0.000 | NA | NA | NA | NA |
