# Supplementary material for: A Two-Component Regulatory System Impacts Extracellular Membrane-Derived Vesicle Production in Group A Streptococcus
Source: mBio. 2016 Nov 1;7(6):e00207-16. doi: 10.1128/mBio.00207-16 (PMC5090034; doi:10.1128/mBio.00207-16)
Supplement: Table S4 — Strains and primers used in this study. [file mbo005163043st4.docx]

| **Table S4 Strains and primers used in this study** | | | |  |
| --- | --- | --- | --- | --- |
| **Strain** | ***emm* type** | **Description** | | **Source** |
| ISS3348 | 1 | Clinical isolate; 30-bp deletion in *covS* | | G. Teti, University of Messina, Italy |
| SF370 | 1 | Reference strain; wild type *covRS* sequence | | ATCC-700294 |
| 3348Δ*hasA* | 1 | ISS3348 with complete deletion of *hasA* coding sequence | | This study |
| 370Δ*hasA* | 1 | SF370 with in-frame *hasA* deletion by allelic replacement | | W. Smith, Newcastle University, UK |
| 370Δ*speB* | 1 | SF370 with complete deletion of *speB* coding sequence | | This study |
| 5448 | 1 | Clinical STSS/NF isolate; wild type *covRS* sequence | | M. Walker, University of Queensland, Australia |
| 5448AP | 1 | Animal passaged variant of 5448 with 1-bp insertion in *covS* | | M. Walker, University of Queensland, Australia |
| NS88.2 | 98,1 | Clinical bacteremia isolate; nonsense G581A mutation in *covS* | | M. Sanderson-Smith, University of Wollongong, Australia |
| 88.2*rep* | 98,1 | NS88.2 with *covS* G581A mutation repaired | | M. Sanderson-Smith, University of Wollongong, Australia |
| 88.2*covS* | 98,1 | 88.2*rep* with original *covS* G581A mutation restored | | M. Sanderson-Smith, University of Wollongong, Australia |
| **Primer** | **Sequence (5' - 3')** | | |  |
| ***covRS qPCR primers*** | | |  |  |
| proS F | GCCTTGGCAGAAGTTGAGAC | |  |  |
| proS R | GCAAAGCAACAACAGGTTCA | |  |  |
| hasA F | AGCGTGCTGCTCAATCATTA | | |  |
| hasA R | CATCCCCAATGCTAACAGGT | | |  |
| speB F | CAAATCAACCGTGGCGACTT | | |  |
| speB R | AGAAGTTACGTCCGTCAGCA | | |  |
| slo F | GACCTATCCAGCAGCCCTTC | | |  |
| slo R | ACCGTTGCTTTGTCTCCCAT | | |  |
| grab F | CTGCGGTTGTTAAAGCGGAT | | |  |
| grab R | GCGATTGGAGTTGCTGCTTT | | |  |
| ***MV qPCR primers*** | | | |  |
| adcR F | TCAGGCGGCAGTAACTAAGG | |  |  |
| adcr R | TGATGGGTATGTTCTGACGCAA | |  |  |
| rexA F | TGCACAGAAAGTAGTAAGTCGCT | | |  |
| rexA R | ACACCGGTGCCTCTTTTTGA | | |  |
| slo F | AACCACAACGACAAATGAGCA | | |  |
| slo R | AGTGGCATTTCTTTGGGAGCA | | |  |
| nagB F | GCTTGGGATTGGGCGTAATG | |  |  |
| nagB R | GCTTGTTTTGGCACATCCTCA | |  |  |
| sagA F | CTACTAGTGTAGCTGAAACAACTCA | |  |  |
| sagA R | ACTTCCGCTACCACCTTGAGA | |  |  |
| amyB F | TGCTGGTGGAGATTTAAAGGGA | | |  |
| amyB R | TCCAAATTGTGGATCAATAGCGT | | |  |
| pfl F | CGATGGTCGTCACCGTGTAA | | |  |
| pfl R | TTCTGGCAGTTGGTCAGTCC | | |  |
| **Primer** | **Sequence (5' - 3')** | | |  |
| nrdG F | GTTGATGGTGAAGGGGTTCG | | |  |
| nrdG R | GCTGTGCCAAATCCGTCATT | | |  |
| salR F | GGATTGGAGATAGCAAAAGAACTCA | | |  |
| salR R | ATCTACAAAACCGTATGCTCCTA | | |  |
| artP F | CAGCCTTGAGCTTGTTGGAC | | |  |
| artP R | ATCCATAGCCAGTCCACGC | | |  |
| ***Cloning primers*** | | | |  |
| OLEC114 | TAACTTGGATCCTAGTTGATGTCAAAAATACGT | | | |
| OLEC115 | TAAACAGGTACCTTTTTTTATACCTCTTTCAAAATAAG | | | |
| OLEC116 | TAAGTTGGTACCTATGGAAATGCATTTCGTTAGAAC | | | |
| OLEC117 | AATCAAGAATTCGACTTTGTTGGCATATTGTGTCTC | | | |
| OLEC2712 | TTGCAATTAGTTCTGGGCTAC | | |  |
| OLEC2713 | TATAATGTATGCTATACGAACGGTAAATTACACCTCTTTCTTTTTTAATTTCC | | | |
| OLEC2714 | TATAGCATACATTATACGAACGGTAAATAATATGTGCATCGAGTAGTTAGAGA | | | |
| OLEC2715 | CCCGCTCTTCTAAGACGTT | | |  |
| OLEC2716 | CGGGATCCCGTGGGCTACCATATTCTGACA | | | |
| OLEC2717 | CGGAATTCCGTGCTTAGCAATATAGGACTTGC | | | |
| OLEC1931 | TACCGTTCGTATAGCATACATTATACGAAGTTATTGGAGCCACCCGCAGTTCG | | | |
|  | AAAAAT | | |  |
| OLEC1932 | TACCGTTCGTATAATGTATGCTATACGAAGTTATTTATTTCCTCCCGTTAAAT | | | |
|  | AATAGATAACTATTAAA | | |  |
